# Beyond factor H: The impact of genetic-risk variants for age-related macular degeneration on circulating factor-H-like 1 and factor-H-related protein concentrations

**DOI:** 10.1016/j.ajhg.2021.05.015

**Published:** 2021-07-13

**Authors:** Valentina Cipriani, Anna Tierney, John R. Griffiths, Verena Zuber, Panagiotis I. Sergouniotis, John R.W. Yates, Anthony T. Moore, Paul N. Bishop, Simon J. Clark, Richard D. Unwin

**Affiliations:** 1 William Harvey Research Institute, Queen Mary University of London, London, EC1M 6BQ, United Kingdom; 2 UCL Institute of Ophthalmology, University College London, London, EC1V 9EL, United Kingdom; 3 Moorfields Eye Hospital National Health Service Foundation Trust, London, EC1V 2PD, United Kingdom; 4 UCL Genetics Institute, University College London, London, WC1E 6BT, United Kingdom; 5 Division of Cardiovascular Sciences, School of Medical Sciences, Faculty of Biology, Medicine, and Health, The University of Manchester, Manchester, M13 9NY, United Kingdom; 6 Department of Epidemiology and Biostatistics, Imperial College London, London, W2 1PG, United Kingdom; 7 Division of Evolution and Genomic Sciences, School of Biological Sciences, Faculty of Biology, Medicine, and Health, University of Manchester, Manchester, M13 9PT, United Kingdom; 8 Manchester Centre for Genomic Medicine, Saint Mary’s Hospital, Manchester University National Health Service Foundation Trust, Manchester, M13 9WL, United Kingdom; 9 Department of Medical Genetics, University of Cambridge, Cambridge, CB2 0QQ, United Kingdom; 10 Ophthalmology Department, University of California San Francisco, San Francisco, CA 94143-0730, USA; 11 Manchester Royal Eye Hospital, Manchester University NHS Foundation Trust, Manchester Academic Health Science Centre, Manchester, M13 9WL, United Kingdom; 12 University Eye Clinic, Department for Ophthalmology, Eberhard Karls University of Tübingen, Tübingen, Baden-Württemberg, 72076, Germany; 13 Institute for Ophthalmic Research, Eberhard Karls University of Tübingen, Tübingen, Baden-Württemberg, 72076, Germany; 14 Lydia Becker Institute of Immunology and Inflammation, Faculty of Biology, Medicine, and Health, University of Manchester, Manchester, M13 9PT, UK; 15 Stoller Biomarker Discovery Centre and Division of Cancer Sciences, School of Medical Sciences, Faculty of Biology, Medicine, and Health, The University of Manchester, Manchester, M13 9NQ, United Kingdom

**Keywords:** age-related macular degeneration, complement factor H, factor H-related, mass spectrometry, Mendelian randomization

## Abstract

Age-related macular degeneration (AMD) is a leading cause of vision loss; there is strong genetic susceptibility at the complement factor H (*CFH*) locus. This locus encodes a series of complement regulators: factor H (FH), a splice variant factor-H-like 1 (FHL-1), and five factor-H-related proteins (FHR-1 to FHR-5), all involved in the regulation of complement factor C3b turnover. Little is known about how AMD-associated variants at this locus might influence FHL-1 and FHR protein concentrations. We have used a bespoke targeted mass-spectrometry assay to measure the circulating concentrations of all seven complement regulators and demonstrated elevated concentrations in 352 advanced AMD-affected individuals for all FHR proteins (FHR-1, p = 2.4 × 10^−10^; FHR-2, p = 6.0 × 10^−10^; FHR-3, p = 1.5 × 10^−5^; FHR-4, p = 1.3 × 10^−3^; FHR-5, p = 1.9 × 10^−4^) and FHL-1 (p = 4.9 × 10^−4^) when these individuals were compared to 252 controls, whereas no difference was seen for FH (p = 0.94). Genome-wide association analyses in controls revealed genome-wide-significant signals at the *CFH* locus for all five FHR proteins, and univariate Mendelian-randomization analyses strongly supported the association of FHR-1, FHR-2, FHR-4, and FHR-5 with AMD susceptibility. These findings provide a strong biochemical explanation for how genetically driven alterations in circulating FHR proteins could be major drivers of AMD and highlight the need for research into FHR protein modulation as a viable therapeutic avenue for AMD.

## Introduction

Age-related macular degeneration (AMD) is a major cause of sight loss and is estimated to affect about 290 million people by 2040.^1^ A total of 34 different genetic loci (including 45 common and seven rare genetic variants) have been reported to be strongly associated with AMD risk;^2^ many of these are linked to genes of the complement system, particularly those encoded on chromosomal region 1q31.3 at the “regulators of complement activation” (RCA) locus.^3^ The RCA locus contains a gene cluster that regulates the alternative pathway of complement, including complement factor H (*CFH*) and five complement-factor-H-related (CFHR) genes. *CFH* encodes full-length factor H (FH) and a truncated splice variant, factor-H-like protein 1 (FHL-1), whereas the CFHR genes encode five FHR proteins, from FHR-1 to FHR-5. FH, FHL-1, and FHR-1 to FHR-5 are synthesized primarily in the liver (Figure S1), although there is evidence for local synthesis within the eye of FH and FHL-1.^4^ FH and FHL-1 are cofactors for factor I, which cleaves and inactivates the central C3b protein in the complement pathway and ensures that activation is kept in check. Although the functions of FHR proteins are less well understood, there is increasing evidence that they compete with the actions of FH and FHL-1 and thereby slow the rate of C3b breakdown and stimulate complement activation.^5^ AMD is a condition that primarily affects the choroid, Bruch’s membrane, and retinal pigment epithelium underlying the neurosensory retina, and there is strong evidence that complement over-activation in this complex has a central role in the condition.^6^^,^^7^

*CFH* has undoubtedly been implicated in AMD susceptibility since the ground-breaking discovery of common risk-associated single-nucleotide polymorphisms, including coding variant rs1061170 (p.Tyr402His), within the gene;^8^, ^9^, ^10^, ^11^ this has been corroborated by many studies that identified rare, highly penetrant, AMD-risk-associated coding variants in *CFH*.^12^, ^13^, ^14^, ^15^ Downstream of *CFH*, a common deletion of *CFHR1* and *CFHR3* and a rare deletion encompassing *CFHR1* and *CFHR4* are associated with decreased risk of AMD.^16^, ^17^, ^18^, ^19^, ^20^, ^21^, ^22^ However, as with other complex traits, the majority of the AMD-associated variants at the RCA locus, and indeed overall, are non-coding (i.e., six out of the eight independent association signals established on chromosomal region 1q.31.3 by the recent largest genome-wide association study (GWAS) of AMD are intronic or intergenic)^2^ and are likely to manifest their effects on disease risk through genetic regulatory mechanisms.^23^ Recently, it has been found that increased circulating FHR-4 is strongly associated with increased risk of AMD.^24^ These observations led us to investigate whether the circulating concentrations of other FHR proteins are associated with AMD. The FHR proteins share high sequence homology (see Figure 3 of Clark and Bishop^25^ for an explanatory diagram), whereas FHL-1 is a splice variant expressed from the same *CFH* gene as FH. This makes it challenging to develop antibody-based assays that can specifically measure concentrations of all seven gene products from this region in blood samples.

Here, we have developed a liquid-chromatography-selected reaction-monitoring mass-spectrometry (LC-SRM-MS)-based assay that could simultaneously measure circulating concentrations of FH, FHL-1, and FHR-1 to FHR-5. We used this assay to interrogate samples from a case-control study of AMD. We subsequently discovered that raised concentrations of FHL-1 and all five FHR proteins are strongly associated with AMD risk, and we used Mendelian randomization to assess the effects of these raised concentrations on AMD.

## Material and methods

### Study samples

The Cambridge AMD study is a case-control study with subjects recruited from the southeast and northwest of England between 2002 and 2006.^26^^,^^27^ All affected subjects analyzed had advanced AMD, i.e., choroidal neovascularization (CNV) and/or geographic atrophy (GA). Controls were spouses, partners, or friends of index AMD individuals. Blood samples were obtained at the time of interview; EDTA and lithium-heparin plasma samples were used for DNA extraction and for FH, FHL-1, and FHR1–5 measurements, respectively. Participants were excluded if they had greater than 6 diopters of myopic refractive error or evidence of other inflammatory or retinovascular disease (such as retinal vessel occlusion, diabetic retinopathy, or chorioretinitis) that could contribute to the development of or confound the diagnosis of AMD. All participants described their ancestry as white on a recruitment questionnaire and were confirmed to be of European descent in the genetic analyses. Participants were examined by an ophthalmologist and underwent color stereoscopic fundus photography of the macular region. Images were graded at the Reading Centre, Moorfields Eye Hospital, London, via the International Classification of Age-related Maculopathy and Macular Degeneration.^28^ All participants provided written informed consent for clinical examination, epidemiological data collection, and blood sampling for biochemical and genetic analyses. Ethical approval was obtained from the NRES Committee East Midlands, Derby and adhered to the tenets of the Declaration of Helsinki.

### Preparation of peptide standards

High-purity heavy-labeled synthetic standards, with S-carboxymethylated (CAM) cysteine residues (denoted by a lowercase c), were obtained (Cambridge Research Biochemicals, Cambridge, UK) and diluted to 1 μg/μL with 50:50 acetonitrile:water + 0.1% v/v formic acid prior to storage at −80°C. Peptide sequences were VTY**K**cFE (FH), NGWSPTP**R**cIRVSFTL (FHL-1), ATFcD**F**PKINHGILYDEE (FHR-1), AMFcD**F**PKINHGILYDEE (FHR-2), VAcHPG**Y**GLP**K**AQTTVTcTE (FHR-3), **Y**QcQSYYE (FHR-4), and **R**GWSTPPIcSFT**K**GE (FHR-5). The residue in bold type contained an isotopically heavy amino acid, with mass increases K(+8), R(+10), F(+10), and Y(+10), respectively. A mixed, concentrated standard mixture was subsequently generated with peptides at a final concentration, in ng/μL, of 47.6 (FH), 0.95 (FHL-1), 7.14 (FHR-1), 19 (FHR-2), and 4.76 (FHR-3, FHR-4, and FHR-5). This concentrated standard was stored at −80°C in 5 μL aliquots until use.

### Preparation of plasma samples for LC-SRM-MS

Frozen plasma samples were thawed to room temperature, vortexed for 5 min, and then centrifuged at 13,300 g for 30 min. A 5 μL aliquot was transferred to a 1.5 mL LoBind Eppendorf tube for processing. 90 μL of 50 mM ammonium bicarbonate (pH 7.8), 2 μL ProteaseMAX (Promega) solution (1% w/v in 50 mM ammonium bicarbonate) and 1 μL 500 mM dithiothreitol prepared in 50 mM ammonium bicarbonate was added. This was vortexed briefly, given a pulse spin, and incubated at 56°C for 25 min. After cooling to room temperature, 3 μL 500 mM iodoacetamide (prepared in 50 mM ammonium bicarbonate) was added, sample vortexed briefly, given a pulse spin, and incubated at room temperature in the dark for 15 min.

For protein digestion, 43 μL 50 mM ammonium bicarbonate (pH 7.8), 1 μL ProteaseMAX solution (1% w/v in 50 mM ammonium bicarbonate), and 5 μL 1 μg/μL endoproteinase Glu-C (Roche) were added, and the tube was vortexed and given a pulse spin before being incubated with shaking (400 rpm) for 16 h at 25°C.

We prepared standard peptides for spiking by adding 195 μL 50:50 acetonitrile:water to a 5 μL aliquot of the concentrated standard mixture. 2 μL was added to each digested sample along with 6 μL 10% v/v TFA, and samples were vortexed briefly so they were mixed, then pulse spun. This provided final standard concentrations of 500 nM (FH), 5 nM (FHL-1), 32.75 nM (FHR-1), 86.75 nM (FHR-2), 21.68 nM (FHR-3), 41.5 nM (FHR-4), and 27.5 nM (FHR-5). Samples were then dried in a centrifugal evaporator (Eppendorf) at 45°C. The dried peptides were reconstituted in 50 μL 0.1% v/v TFA and vortexed so that any residue would be dissolved before centrifugation at 13,300 g for 30 min so that any insoluble or particulate material would settle. Taking care to leave behind any precipitated material, we transferred approximately 48 μL to a LC autosampler vial for subsequent analysis by LC-MS/MS.

### LC-SRM-MS analysis of plasma digests

SRM analyses of plasma digests were performed on a 6495 triple quadrupole mass spectrometer with electrospray ion source (Agilent) (source parameters are in Table S1) coupled to an Agilent Infinity 1200 Series liquid chromatography system. Samples were injected directly (4 μL) onto a C18 column (250 mm × 2.1 mm I.D., Thermo Scientific Acclaim 120, 3 μm particle size) maintained at a temperature of 50°C. Peptides were eluted with gradient-chromatography buffer A (water + 0.1% formic acid) and buffer B (acetonitrile + 0.1% formic acid) at a flow rate of 250 μL/min at an initial composition of 5% buffer B. The following gradient was used (time, %B): 0 min, 5% B; 2 min, 5% B; 3 min, 12% B; 12 min, 15% B; 15 min, 20% B; 30 min, 25% B; 31 min, 90% B; 39 min, 90% B; 40 min, 5% B; and 49 min, 5% B. Optimized SRM settings were determined through the use of SIS solutions and are given in Table S2.

So that the source region would be protected from unwanted contaminants, a switching valve located between the column and source was diverted to the waste position at points in the chromatogram when the analyte peptides were not eluting. This allowed for six windows (two of the peptides, FHR-2 and FHL-1, eluted within the same window) of acquisition, of approximately 1 min each, to be acquired with the column on-line to the mass spectrometer.

FH, FHL-1, and the five FHR protein concentrations were determined in plasma samples from the Cambridge AMD cohort.^26^^,^^27^ Samples were randomized into batches such that each batch contained a mixture of experimental and quality-control samples. Alongside 20 experimental samples, each batch contained (1) full technical duplicates on a commercial standard human serum sample, (2) a full technical replicate of one of the samples in the batch, and (3) a full technical replicate of the “duplicated” sample from the previous batch. These allowed for sample-batch quality control and assessment of batch-to-batch variability.

### SRM data extraction and analysis

SRM data were processed via a dedicated project in Skyline (v19.1.0.193).^29^ We visually checked retention times and heavy peptide peak areas for all samples to ensure correct peak allocations and integrations. We extracted peak-area data from Skyline into an Excel workbook, where we compared peak areas between heavy and light transitions for each peptide. We calculated the on-column loading of endogenous peptide by using the largest signal as a quantifier and the other two transitions as qualifier signals to confirm specificity and agreement in quantitation. The on-column loading of endogenous peptide was converted to a concentration per unit volume for each plasma sample on the basis of the injection of an equivalent of 0.8 μL of plasma for each sample.

### Factor H measurement by ELISA

Human factor H ELISAs (Abcam) were performed on a subset of samples from the full cohort as per the manufacturer’s instructions. Plasma samples were serially diluted to 1:50,000 prior to use. A mix of affected individuals and controls were selected with an even distribution across the full SRM FH result spectrum. Endpoint results were read at 450 nm with a SpectraMax M5 plate reader (Molecular Devices).

### Association analysis of circulating protein concentrations with AMD

We transformed protein concentrations to ensure normality of the distribution (by using the square-root function for FH and FHR-2; FHR-3 and FHR-4; and the log function for FHL-1 and FHR-5; FHR-1 was normally distributed) when we used linear-regression models. We assessed the association of advanced AMD with concentrations of FH, FHL-1, and each of the five FHR proteins via Wald tests by using linear-regression models adjusted for sex, age, and the first two genetic principal components (as estimated within the International AMD Genomics Consortium [IAMDGC] study).^2^ We also reported the association of protein concentrations with advanced AMD via odds ratio (OR) expressed as a per-one-standard-deviation (SD) change of log levels by using logistic-regression models adjusted for sex, age, and the first two genetic principal components. These statistical analyses were conducted with Stata software version 14.2 (StataCorp).

### Genotype data and genome-wide association analyses

All individuals included in this study had been previously genotyped with a custom-modified Illumina HumanCoreExome array at the Centre for Inherited Disease Research (CIDR, Baltimore, Maryland, USA) and analyzed within the IAMDGC GWAS (43,566 subjects; 16,144 individuals with advanced AMD and 17,832 controls of European ancestry in the primary analysis dataset).^2^ Quality-control and genotype imputation based on the 1000 Genomes Project^30^ reference panel were performed by the IAMDGC as described previously.^2^ We carried out GWASs of concentrations of FH, FHL-1, and all five FHR proteins (we transformed concentrations as above to ensure normality) in controls only by using linear-regression models adjusted for sex, age, and the first two genetic principal components and variants with minor-allele frequency (MAF)^3^ ≥ 1% (and imputation quality, R^2^,≥ 0.3, if imputed). The GWASs were carried out with the EPACTS software (version 3.3.2), and Wald tests were performed on the variant genotypes coded as 0, 1, and 2 according to the number of minor alleles for the directly typed variants or allele dosages for the imputed variants. Manhattan and Q-Q plots were generated with the *qqman* R package (version 0.1.4). Regional plots of association were generated with LocusZoom.org. Finally, linkage disequilibrium (LD) measures (*R*^2^ and *D'*) were calculated with LDlink (version 5.0) on the basis of the European (EUR) population genotype data originated from phase 3 (version 5) of the 1000 Genomes Project.^30^

### Mendelian-randomization analysis

We used a Mendelian-randomization approach to test whether genetically proxied FHR protein concentrations are associated with risk of AMD. We used p value clumping to select independent genetic variants associated with the exposure (a protein at a time) at genome-wide significance level (p < 5 × 10^−8^) as instrumental variables (IVs) (function *ld_clump* of R package *ieugwasr*, version 0.1.5; LD cut-offs R^2^ < 0.001 and R^2^ < 0.01, and the default 1000 Genomes Project EUR population reference,^30^ n = 489, were used for estimating LD among genetic variants). We evaluated the strength of each IV by using R^2^ as the proportion of the variance of the protein explained by the genetic variant (function *get_r_from_pn* from R package *TwoSampleMR*, version 0.5.5). We also repeated the IV selection by using the GCTA-COJO^31^ approach (with default settings). As a reference sample to estimate LD among genetic variants, we used the available individual-level genotype data from the entire control set in the Cambridge AMD study,^2^^,^^26^^,^^27^ n = 419, and thus ensured that the same set of variants analyzed in the GWASs of FHR protein concentrations were also used for estimating LD. It is common in Mendelian-randomization analyses of unmatched case-control studies to estimate the association of the IV with the exposure within the controls only; the justification behind this approach is that the distributions of the exposure in the general population and the control group are similar when the disease prevalence is low and that the association between the outcome and the expected exposure value conditioned on the IV is unconfounded under the IV assumptions.^32^ Therefore, we carried out the IV selection by using the GWAS findings on the Cambridge AMD study^26^^,^^27^ controls for whom we measured protein concentrations (n = 252).

If a single IV was available, we used the ratio-of-coefficients method, also known as the Wald method, to estimate the effect of genetically proxied protein concentrations on the disease risk.^33^ The Wald ratio for a single genetic variant as IV is defined as its genetic association with the outcome (i.e., risk of AMD) over the genetic association with the exposure (i.e., protein concentration). Using a one-sample approach, we derived the genetic association with the exposure from the GWASs of the available FHR protein concentrations in the Cambridge AMD study^26^^,^^27^ control individuals only (n = 252). The genetic associations with the risk of AMD were obtained from the summary GWAS estimates on the basis of a logistic-regression model with AMD status as the outcome observed in the Cambridge AMD study^26^^,^^27^ (419 controls and 845 affected individuals). If multiple IVs were available for a protein, we used the inverse-variance weighted (IVW) method under a fixed-effect model^33^ (function *mr_ivw* from R package *MendelianRandomization*, version 0.4.2). We assessed heterogeneity across the different single-IV estimates by using the Cochran’s Q and I^2^ statistics. Additionally, we calculated analogous Mendelian randomization estimates by using a two-sample approach whereby we measured the genetic association with the exposure from the FHR-concentration GWASs conducted on the Cambridge AMD study^26^^,^^27^ controls only (n = 252) and the genetic associations with the AMD risk observed in the IAMDGC GWAS^2^ (16,144 advanced AMD affected individuals and 17,832 control individuals of European ancestry).

## Results

### Development of an assay for FH, FHL-1, and FHR-1 to FHR-5

To facilitate the detection of the five FHR proteins, FH, and the splice variant FHL-1, we used mass spectrometry because this provides the necessary specificity to allow confident detection of and differentiation between similar proteins or proteoforms. Standard trypsin hydrolysis of FHL-1 yields a specific N-terminal peptide of only four amino acids, which is challenging for MS detection. Therefore, we developed an approach utilizing Endoproteinase Glu-C (V8 Protease) to produce not only distinct proteotypic peptides for all of the FHR proteins but also a unique proteotypic peptide representative of FHL-1. These peptides can thus be used for the simultaneous detection and relative quantification of all seven key regulatory proteins in a plasma sample in a single assay.

To confirm the specificity and quantitative ability of the assay, we established optimal SRM transitions on the basis of fragmentation of synthetic versions of each peptide of interest (Figure S2). Assay specificity was determined by analysis both of human plasma and of serum samples with and without synthetic peptides spiked in. Subsequently, serum samples containing stable isotope standards (SIS) peptides was analyzed. Figure S3 shows an overlay of endogenous and SIS peptides in human serum, confirming specificity.

**Figure 1:**
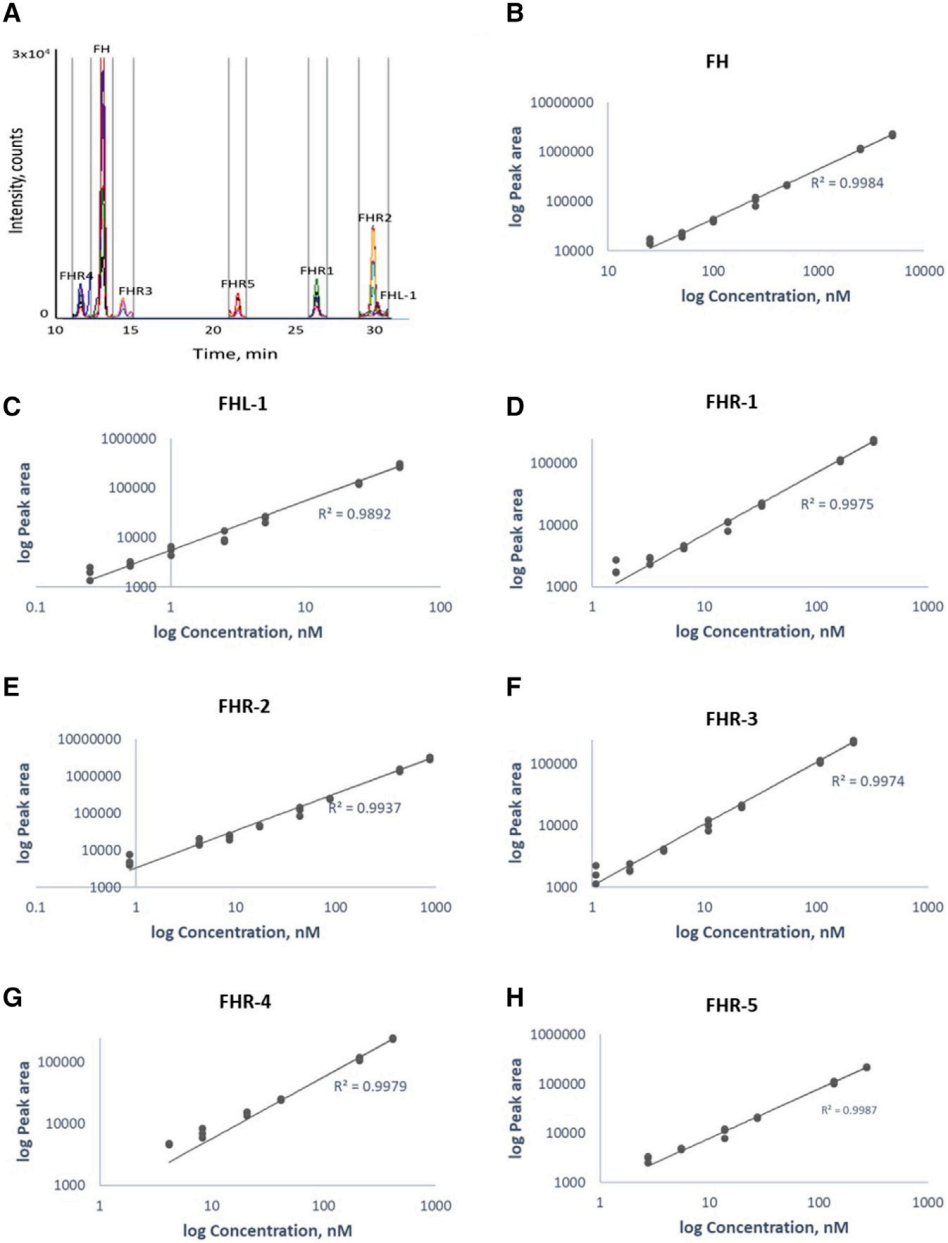
Development of a method for quantification of FH, FHL-1, and FHR-1 to FHR-5 (A) A total ion chromatogram from a typical LC-SRM-MS analysis of a plasma sample demonstrates low background, lack of interferences, and specific signals for each peptide. (B–H) Assay linearity of standards in a plasma matrix showing linearity over the sample concentration range for each analyte.

A typical chromatogram for the assay is shown in Figure 1A. Note that, to prevent dirtying and signal decay, we diverted flow away from the source when analytes were not eluting. We determined quantitative performance by generating standard curves with the SIS peptides spiked into a Glu-C plasma digest. The assay shows excellent linearity across the dilution range (Figures 1B–1H). This allowed determination of lower limits of quantitation, defined as plasma concentrations of FH = 25 nM, FHL-1 = 0.25 nM, FHR-1 = 2 nM, FHR-2 = 1 nM, FHR-3 = 1 nM, FHR-4 = 4 nM, and FHR-5 = 3 nM.

Assay reproducibility was confirmed across all batches of samples. Duplicate commercial samples included in all batches across the experiment (74 measurements over 37 batches) demonstrated %CV for each of the following proteins: FH = 13.4%, FHL-1 = 21.0%, FHR-1 = 18.3%, FHR-2 = 15.3%, FHR-3 = 14.4%, FHR-4 = 14.6%, and FHR-5 = 9.7% (Figure S4). Triplicate AMD samples analyzed across adjacent batches were highly reproducible; 91.8% of measurements resulted in %CV < 15% (Table S3). Measured concentration for each sample is provided in Table S4 and summarized in Table 1. To further validate our assay, we also measured FH concentrations by using a commercial ELISA and compared the results to our MS data; normalized values from both approaches were generally within ∼20% (Figure S5) of each other.

**Table 1:** Demographics of study samples and association analyses between AMD and circulating concentrations of FH, FHL-1, and FHR-1 through FHR-5

**Characteristics**	**Control individuals**	**AMD-affected individuals**		
N	252	352		
Age, yr (SD)	75.2 (7.9)	73.9 (8.3)		
Male (%)	39.3	45.7		
AMD phenotype				
CNV only		218		
GA only		73		
Mixed		61		

**Protein concentrations**	**nM (95% CI)**	**nM (95% CI)**	**Association with AMD, beta, SE, *P***^a^	**OR (95% CI)**^b^

Mean FH concentrations	737.3 (718.2–756.5)	736.5 (721.3–751.6)	0.005, 0.23, 0.982 (0.02, 0.23, 0.936)	1.01 (0.86–1.20)
Mean FHL-1 concentrations	10.4 (10.1–10.8)	11.3 (11.0–11.7)	0.08, 0.02, 1.4 × 10^−3^ (0.08, 0.02, 4.9 × 10^−4^)	1.35 (1.14–1.60)
Mean FHR-1 concentrations	31.2 (29.4–32.9)	38.4 (37.0–39.8)	7.22, 1.12, 2.1 × 10^−10^ (7.21, 1.12, 2.4 × 10^−10^)	1.81 (1.47–2.24)
Mean FHR-2 concentrations	45.3 (43.1–47.6)	55.3 (53.2–57.4)	0.71, 0.12. 1.9 × 10^−9^ (0.74, 0.12, 6.0 × 10^−10^)	1.66 (1.38–1.98)
Mean FHR-3 concentrations	24.1 (21.7–26.5)	28.9 (27.1–30.8)	0.55, 0.13, 4.4 × 10^−5^ (0.59, 0.13, 1.4 × 10^−5^)	1.54 (1.29–1.84)
Mean FHR-4 concentrations	46.1 (42.7–49.6)	53.8 (50.5–57.1)	0.53, 0.17, 2.1 × 10^−3^ (0.56, 0.17, 1.3 × 10^−3^)	1.27 (1.08–1.50)
Mean FHR-5 concentrations	25.5 (24.5–26.5)	27.9 (27.0–28.9)	0.09, 0.03, 1.9 × 10^−4^ (0.10, 0.03, 1.9 × 10^−4^)	1.38 (1.16–1.63)

Abbreviations are as follows: AMD = age-related macular degeneration; CNV = choroidal neovascularization; GA = geographic atrophy; SE = standard error; and CI = confidence interval.

a Wald tests using linear-regression models; adjusted p values for sex, age, and the first two genetic principal components as estimated in Fritsche et al.^2^ are displayed in parentheses

b Odds ratio (OR) of advanced disease expressed as the per-standard-deviation change of log levels in logistic-regression models adjusted for sex, age, and the first two genetic principal components.

### Circulating FHL-1 and FHR-1 to FHR-5 concentrations are higher in people with advanced AMD

**Figure 2:**
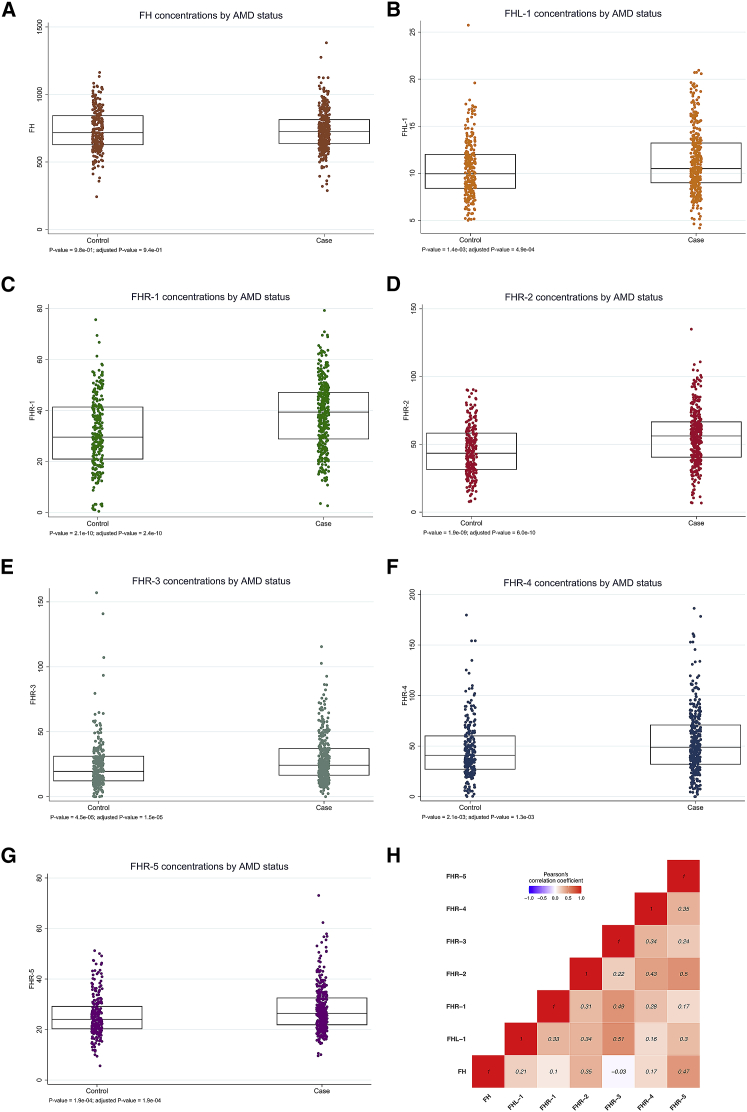
Circulating concentrations of FHL-1 and FHR-1 to FHR-5 are elevated in AMD-affected individuals Boxplots of FH (A), FHL-1 (B), and FHR-1 to FHR-5 (C–G) protein concentrations measured in plasma samples of 352 individuals with advanced AMD and 252 phenotyped control individuals from the Cambridge AMD study.^26^^,^^27^ Protein concentrations are expressed as nM. Individuals with AMD show statistically significant elevated concentrations of FHL-1 and FHR-1 through FHR-5 in comparison to controls, whereas no significant difference between individuals with AMD and controls was observed for FH concentrations. Unadjusted p values were obtained from Wald tests via linear-regression models and are presented together with p values adjusted for sex, age, and the first two genetic principal components (as estimated within the IAMDGC study^2^). (H) Pairwise Pearson’s correlation coefficients (r) between the seven protein concentrations for the 252 control samples. Some modest correlation is observed between FH and FHR-5, FHL-1 and FHR-3, FHR-1 and FHR-3, and FHR-2 and FHR-5.

Using our newly developed assay, we measured circulating concentrations of FH, FHL-1, and FHR-1 to FHR-5 in plasma samples of 352 individuals with advanced AMD and 252 phenotyped controls from the Cambridge AMD study^26^^,^^27^ (Table 1, Figure 2). Although no significant difference between AMD-affected individuals and controls (p value = 0.94) was observed for FH, AMD-affected individuals showed significantly higher concentrations of FHL-1 and all five FHR proteins than did control individuals (FHL-1, β = 0.08 and p = 4.9 × 10^−4^; FHR-1, β = 7.21 and p = 2.4 × 10^−10^; FHR-2, β = 0.74 and p = 6.0 × 10^−10^; FHR-3, β = 0.59 and p = 1.5 × 10^−5^; FHR-4, β = 0.56 and p = 1.3 × 10^−3^; FHR-5, β = 0.10 and p = 1.9 × 10^−4^) (Table 1, Figures 2A–2G). The adjusted ORs of advanced AMD for a 1 SD increase of log-transformed concentrations are also presented in Table 1. Correlation analysis in the control samples demonstrated little pairwise correlation between FHRs; there was only some modest correlation between FH and FHR-5, between FHL-1 and FHR-3, between FHR-1 and FHR-3, and between FHR-2 and FHR-5 (Figure 2H). In general, it does not follow that an individual with high concentrations of one FHR protein will have higher concentrations of the others. As such, determining the concentrations of all FHR proteins is most likely important.

### Genetic determinants of circulating concentrations of complement regulatory proteins overlap with the AMD-associated *CFH* locus

**Figure 3:**
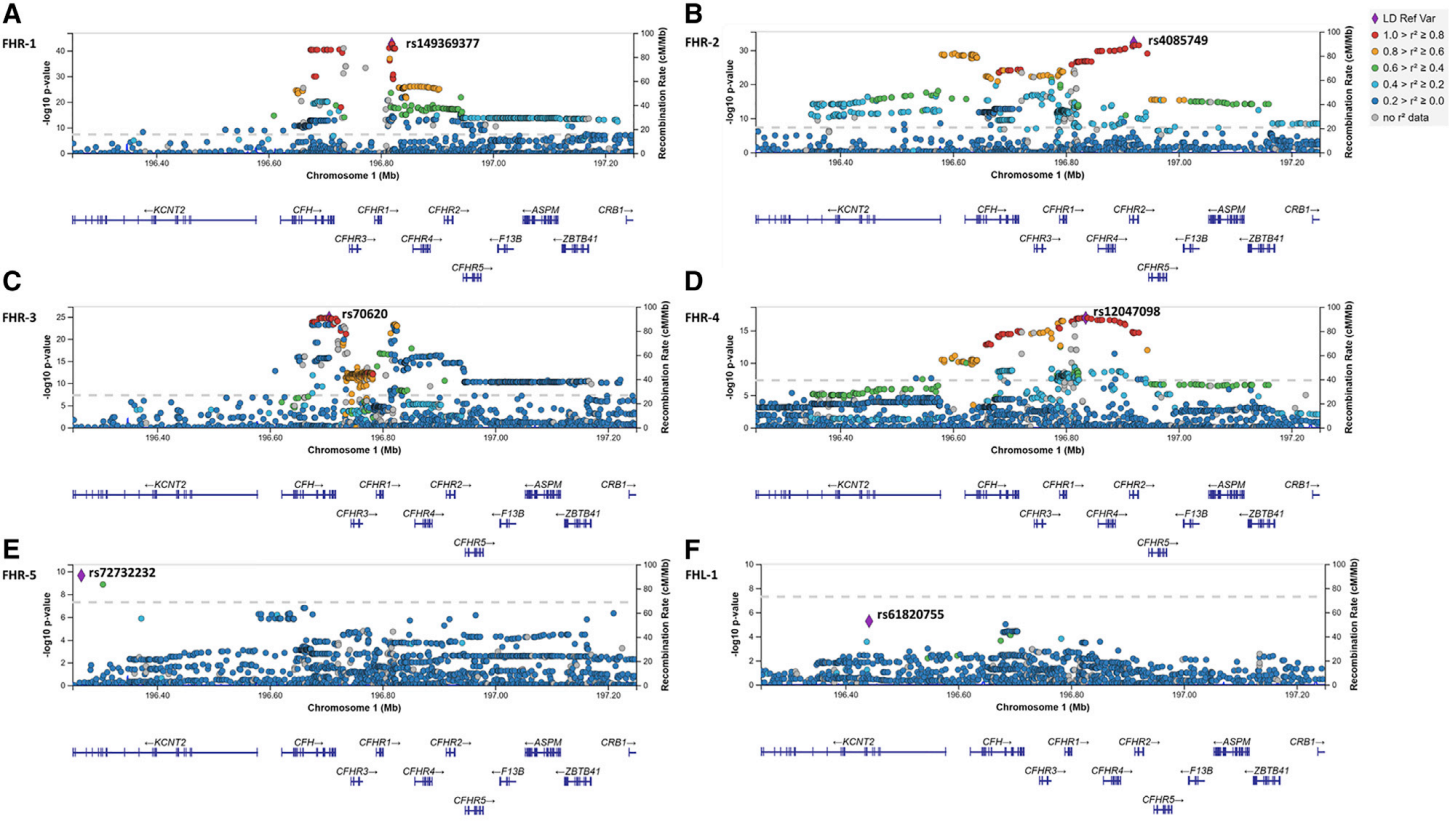
GWASs of circulating concentrations of FHR-1 through FHR-5 reveal a strong genome-wide-significant signal spanning the *CFH* locus Regional plots show the genome-wide-significant (p < 5 × 10^−8^) association signals from the GWASs of FHR-1 through FHR-5 concentrations (A–E) at the *CFH* locus on chromosomal region 1q31.3. (F) The equivalent *CFH* region for the GWAS of FHL-1 concentrations (no genome-wide-significant association regions were observed). The most associated variant is denoted by a purple diamond and is labeled by its rs number. The other surrounding variants are shown by circles colored to reflect the extent of linkage disequilibrium with the most associated variant (on the basis of the European [EUR] population genotype data originated from the 1000 Genomes Project, November 2014). A diagram of the genes within the relevant regions is depicted below each plot. Physical positions are based on NCBI RefSeq hg19 human genome reference assembly.

We performed genome-wide association analyses of the protein concentrations that were found to be elevated in individuals with advanced AMD (i.e., FHL-1 and FHR-1 to FHR-5). All GWASs of the concentrations of the five FHR proteins in 252 control individuals showed a genome-wide-significant (p < 5 × 10^−8^) peak at the *CFH* locus (Figure 3, Figure S6, Table S5). For FHR-1, FHR-2, FHR-4, and FHR-5, the *CFH* locus displayed the only observed genome-wide-significant peak for which, in the case of all the top signals, the direction of allelic effect on concentrations was concordant with the direction of effect on disease, as estimated in the IAMDGC GWAS study^2^ (Table 2). FHR-3 showed a more polygenic profile, with genome-wide-significant signals at rs113721756 on chromosome 10 (p = 1.7 × 10^−8^), rs111260777 on chromosome 11 (p = 1.5 × 10^−9^), rs117468955 on chromosome 12 (p = 3.0 × 10^−8^), rs4790395 on chromosome 17 (p = 3.6 × 10^−8^), rs117115124 on chromosome 19 (p = 2.5 × 10^−8^), and rs78606172 on chromosome 20 (p = 3.9 × 10^−11^), in addition to the *CFH* locus (Table 2). The strongest signal from the GWAS of FHL-1 concentrations was observed at rs200404865 on chromosome 13 (p = 9.6 × 10^−7^), and the strongest signal at the *CFH* locus was observed at intronic *KCNT2* variant rs61820755 (p = 5.3 × 10^−6^). We also observed a block of variants in high or complete LD with the top AMD-associated intronic *CFH* variant rs10922109 [1.1] from the IAMDGC GWAS^2^ as the forth signal (p = 3.7 × 10^−5^) (Figure 3, Figure S6, Table S5).

**Table 2:** Instrumental variables (IVs) of FHR protein concentrations and their corresponding genetic-association estimates for FHR protein concentrations and AMD

**Protein**	**Instrumental variable (IV) : dbSNP ID; (Chr: position)**^a^**; non-effect allele/effect allele**	***cis*/*trans* pQTL**	**IV strength (R**^**2**^**)**^b^	**Association with protein concentrations in 252 Cambridge controls**			**Association with AMD in the Cambridge AMD GWAS**^26^^,^^27^**(845 AMD-affected individuals and 419 control individuals)**			**Association with AMD in the IAMDGC GWAS**^2^**(16,144 AMD-affected individuals and 17,832 controls)**			
				**Beta**	**SE**	**p value**	**Beta**	**SE**	**p value**	**Beta**	**SE**	**p value**	**Minor-allele frequency**
FHR-1	rs149369377; (1: 196819479_A/G); *CFHR2* intronic	*cis*	0.53	−18.15	1.07	2.6 × 10^−43^	−0.76	0.13	1.9 × 10^−9^	−0.87	0.02	7.6 × 10^−295^	0.157
FHR-2	rs4085749; (1: 196920148_C/T); *CFHR2* synonymous	*cis*	0.44	−1.55	0.11	6.3 × 10^−33^	−0.78	0.11	2.0 × 10^−12^	−0.62	0.02	2.2 × 10^−184^	0.192
FHR-3	rs70620; (1:196704997_G/A); *CFH* intronic	*cis*	0.35	2.02	0.17	1.5 × 10^−25^	−0.06	0.12	0.581	−0.07	0.02	2.7 × 10^−3^	0.162
	rs78606172; (20: 62087676_G/A); *KCNQ2* intronic	*trans*	0.16	7.61	1.10	3.9 × 10^−11^	−0.20	0.56	0.721	0.14	0.11	0.210	0.010
	rs111260777; (11: 127117796_T/C); intergenic	*trans*	0.14	4.36	0.69	1.5 × 10^−9^	−0.04	0.47	0.924	−0.01	0.07	0.865	0.018
	rs113721756; (10: 116647277_C/T); *FAM160B1* intronic	*trans*	0.12	4.92	0.84	1.7 × 10^−8^	−0.76	0.54	0.161	0.12	0.09	0.187	0.012
	rs11711512; (19: 56030803_G/A); intergenic	*trans*	0.12	4.57	0.79	2.5 × 10^−8^	0.75	0.45	0.096	0.09	0.10	0.351	0.016
	rs117468955; (12: 92269900_A/G); intergenic	*trans*	0.12	4.72	0.82	3.0 × 10^−8^	0.06	0.53	0.913	−0.03	0.10	0.789	0.015
	rs4790395; (17: 2852632_C/T); *RAP1GAP2*	*trans*	0.11	−6.50	1.14	3.6 × 10^−8^	−0.04	0.78	0.955	0.16	0.13	0.206	0.031
FHR-4	rs12047098; (1: 196835106_T/C); *CFHR2* intronic	*cis*	0.25	−1.75	0.19	1.1 × 10^−17^	−0.86	0.12	8.9 × 10^−14^	−0.67	0.02	5.5 × 10^−198^	0.172
FHR-5	rs72732232; (1: 196265545_T/A); *KCNT2* intronic	*cis*	0.15	−0.52	0.08	2.2 × 10^−10^	−1.56	0.32	1.4 × 10^−6^	−0.86	0.07	3.2 × 10^−41^	0.026

Number of genetic variants associated with concentrations of a protein at genome-wide significance level (p < 5 × 10^−8^): FHR-1: 529 on chromosome 1; FHR-2: 553 on chromosome 1; FHR-3: 611 on chromosome 1, 1 on chromosome 10, 2 on chromosome 11, 1 on chromosome 12, 1 on chromosome 17, 3 on chromosome 19, 1 on chromosome 20; FHR-4: 253 on chromosome 1; FHR-5: 2 on chromosome 1. IVs were selected using the P value clumping method. P value clumping was performed with function *ld_clump* of R package *ieugwasr*, version 0.1.5; LD cut-offs R^2^ < 0.001 and the 1000 Genomes Project EUR population reference.^30^

AMD = Age-Related macular degeneration; GWAS = Genome-wide association study; IAMDGC = International Age-Related Macular Degeneration Genomics Consortium; pQTL = protein quantitative trait locus.

a Chromosomal position is given according to the NCBI RefSeq hg19 human genome reference assembly;

b The strength of each IV was evaluated using R^2^ as the proportion of the variance of the protein explained by the genetic variant(s) (function *get_r_from_pn* from R package *TwoSampleMR*, version 0.5.5).

The genome-wide-significant regions of the *CFH* locus as determined from the analyses of concentrations of FHR-1 through FHR-5 overlapped among the different concentrations but showed nominally different top signals (i.e., intergenic between *CFHR1* and *CFHR4* rs149369377 for FHR-1, with p = 2.6 × 10^−43^ and β = −18.2; synonymous *CFHR*2 rs4085749 for FHR-2 with p = 6.3 × 10^−33^ and β = −1.5; intronic *CFH* rs70620 for FHR-3 with p = 1.5 × 10^−25^ and β = 2.0; intergenic between *CFHR1* and *CFHR4* rs12047098 for FHR-4, with p = 1.1 × 10^−17^ and β = −1.7; and intronic *KCNT2* rs72732232 for FHR-5, with p = 2.2 × 10^−10^ and β = −0.5) (Table 2, Figure 3, Table S5). These top signals are not in high LD with each other, except for rs4085749 of FHR-2 and rs12047098 of FHR-4 (R^2^ = 0.83, D′ = 0.95) (Table S6).

Next, we assessed whether the GWAS top signals of FHR-1 through FHR-5 protein concentrations were in LD with any of the independently AMD-associated variants at the *CFH* locus reported by the IAMDGC GWAS,^2^ which also included the Cambridge samples analyzed in this study (i.e., intronic *CFH* rs10922109 [1.1]; intronic *CFH* rs570618 [1.2], proxy for p.Tyr402His; *CFH* R1210C, rs121913059 [1.3]; intergenic rs148553336 [1.4], 8 kb upstream of *CFH* and 35 kb downstream of *KCNT2*; intronic *KCNT2* rs187328863 [1.5]; intergenic rs61818925 [1.6], 14 kb downstream of *CFHR1* and 156 kb upstream of *CFHR4*; intronic *CFH* rs35292876 [1.7]; intronic *CFHR5* rs191281603 [1.8]; Table 3). The rare *CFH* variant rs121913059 (p.Arg1210Cys), [1.3]^13^ was present heterozygously in a single affected individual from the Cambridge study and was excluded from this analysis. The top signal for FHR-1 was in modest LD with the top AMD-associated variant 1.1 (R^2^ = 0.30) and low LD with the proxy for p.Tyr402His 1.2 (R^2^ = 0.12); the top signal for FHR-2 was in modest LD with 1.1 (R^2^ = 0.35) and 1.6 (R^2^ = 0.36) and in low LD with 1.2 (R^2^ = 0.16); similar results were obtained for the top signal of FHR-4 (R^2^ = 0.38, 0.42, and 0.16 with 1.1, 1.6, and 1.2, respectively); low LD was seen with 1.1, 1.2, and 1.6 (R^2^ = 0.16, 0.12, and 0.11, respectively) for the top signal of FHR-3, and the top signal of FHR-5 was in low to modest LD with 1.4 (R^2^ = 0.26) (Table S6).

**Table 3:** Single-variant association analyses for the eight established AMD independently associated variants at the *CFH* locus with concentrations of FH, FHL-1, and FHR-1 to FHR-5 in control individuals

				**Association with protein concentrations in 252 Cambridge AMD study**^26^^,^^27^**control individuals**^a^						
				**FH**	**FHL-1**	**FHR-1**	**FHR-2**	**FHR-3**	**FHR-4**	**FHR-5**
**IAMDGC**^2^**association** **signal number (direction**^b^**)**	**dbSNP ID (Chr: position)**^c^^**;**^**major/minor allele (imputation R**^**2**^**)**^d^	**IAMDGC OR (MAF in controls)**	**MAF, Cambridge controls**	**Beta (SE); p**	**Beta (SE); p**	**Beta (SE); p**	**Beta (SE); p**	**Beta (SE); p**	**Beta (SE); p**	**Beta (SE); p**
1.1 (−)	rs10922109 (1: 196704632); C/A (1.00)	0.38 (0.426)	0.422	0.49 (0.25), 0.056	−0.10 (0.02); 3.7 × 10^−5^	−10.67 (1.04); 7.8 × 10^−21^	−0.75 (0.11); 2.9 × 10^−11^	−1.20 (0.14); 1.7 × 10^−16^	−1.0 (0.17); 1.5 × 10^−9^	−0.04 (0.03); 0.184
1.2 (+)	rs570618 (1: 196657064); G/T (1.00)	2.38 (0.364)	0.357	0.27 (0.26); 0.296	0.05 (0.03); 0.046	8.19 (1.17); 2.0 × 10^−11^	0.85 (0.11); 1.8 × 10^−12^	0.16 (0.16); 0.304	0.62 (0.18); 6.8 × 10^−4^	0.10 (0.03); 7.8 × 10^−4^
1.3 (+)	rs121913059 (1: 196716375); C/T (genotyped)	20.28 (0.00014)	0	*no control carrier observed; not analyzed*						
1.4 (−)	rs148553336 (1: 196613173); T/C (genotyped)	0.29 (0.009)	0.017	−2.29 (1.02); 0.025	−0.09 (0.10); 0.353	0.06 (5.00); 0.990	−1.99 (0.48); 4.6 × 10^−5^	0.33 (0.62); 0.603	0.74 (0.72); 0.302	−0.55 (0.11); 6.3 × 10^−7^
1.5 (+)	rs187328863 (1: 196380158); C/T (0.83)	2.27 (0.028)	0.013	1.12 (1.34); 0.404	0.06 (0.13); 0.660	0.75 (6.52); 0.908	1.13 (0.64); 0.080	−0.51 (0.81); 0.536	0.61 (0.94); 0.515	0.01 (0.15); 0.956
1.6 (−)	rs61818925 (1: 196815450); G/T (0.87)	0.60 (0.385)	0.405	−0.42 (0.27); 0.124	0.001 (0.03); 0.962	0.99 (1.33); 0.459	−0.93 (0.12); 1.3 × 10^−13^	0.85 (0.16); 1.7 × 10^−7^	−1.12 (0.18); 1.5 × 10^−9^	−0.07 (0.03); 0.014
1.7 (+)	rs35292876 (1: 196706642); C/T (genotyped)	2.42 (0.009)	0.004	*MAF ≤ 1%; not analyzed*						
1.8 (+)	rs191281603 (1: 196958651); C/G (0.42)	1.07 (0.006)	0.008	*MAF ≤ 1%; not analyzed*						

Abbreviations are as follows: AMD = age-related macular degeneration; IAMDGC = International Age-Related Macular Degeneration Genomics Consortium; and MAF = minor-allele frequency.

a Wald tests using linear-regression models adjusted for sex, age, and the first two genetic principal components as estimated within the IAMDGC study.^2^

b Direction of association with AMD for the minor allele, as estimated in the IAMDGC study.^2^

c Chromosomal position is given according to the NCBI RefSeq hg19 human genome reference assembly.

d Imputation quality metric R^2^ as estimated in the IAMDGC study.^2^

**Figure 4:**
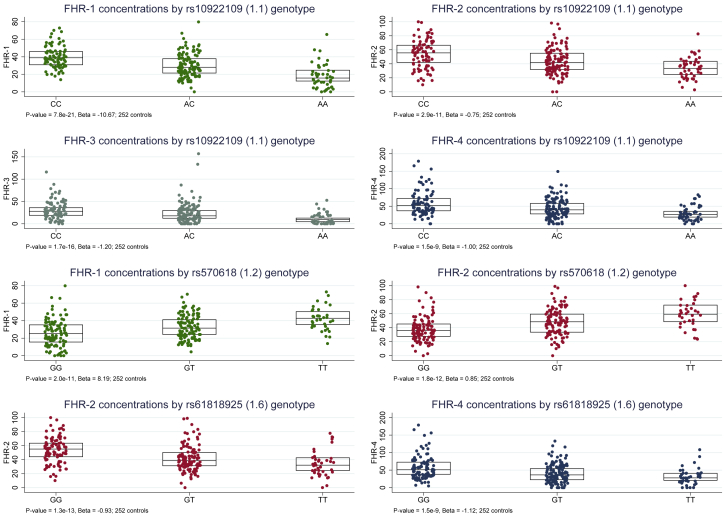
Established AMD-risk variants at the *CFH* locus are associated with circulating FHR-1, FHR-2, FHR-3, and FHR-4 concentrations in control individuals Boxplots of FHR concentrations by variant genotype for those *CFH* variants that were established as conferring AMD risk in the IAMDGC study^2^ and that showed genome-wide-significant (p < 5 × 10^−8^) associations in 252 controls from the Cambridge AMD study^26^^,^^27^ cohort (Table 3). p values and beta values from Wald tests using linear-regression models adjusted for sex, age, and the first two genetic principal components (as estimated within the IAMDGC study) are indicated in the note at the bottom of each plot.

Furthermore, genome-wide-significant associations were observed at the top IAMDGC variant rs10922109 (1.1) with p = 8.6 × 10^−21^, 2.9 × 10^−10^, 2.2 × 10^−16^, and 1.7 × 10^−9^ for FHR-1, FHR-2, FHR-3, and FHR-4, respectively; at the proxy for p.Tyr402His 1.2 with p = 2.0 × 10^−11^ and 1.8 × 10^−12^ for FHR-1 and FHR-2, respectively; and at the variant 1.6 with p = 1.8 × 10^−11^ and 2.4 × 10^−9^ for FHR-2 and FHR-4, respectively. For all these genetic associations, the direction of allelic effect on respective protein concentrations was concordant with that on disease as estimated in the IAMDGC GWAS^2^ (Table 3, Figure 4). Altogether, these GWAS findings support the hypothesis that the *CFH* locus AMD-risk variants increase disease risk through increase of FHR concentrations.

We also carried out a GWAS of the FH concentrations in controls and observed no genome-wide-significant associations; the strongest signal was at the *CFH* locus for intronic *CFHR2* rs114036234 (p = 4.7 × 10^−6^ and β = −3.0), and the direction of allelic effect on concentrations was concordant with that on disease as estimated in the IAMDGC GWAS study^2^ (p = 2.5 × 10^−9^ and β = −0.3) (Figure S6, Table S5).

### Mendelian-randomization estimates of the effects of circulating concentrations of complement regulatory proteins on susceptibility to AMD

**Figure 5:**
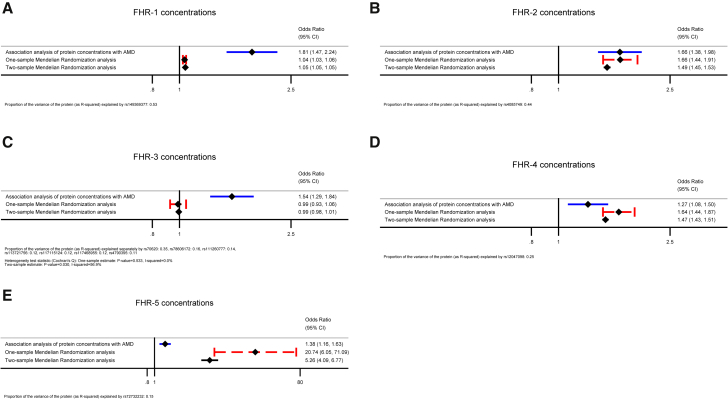
Mendelian-randomization analysis shows a highly significant elevation of circulating FHR-1, FHR-2, FHR-4, and FHR-5 concentrations in advanced AMD One-sample and two-sample Mendelian-randomization estimates of the association of FHR-1 (A), FHR-2 (B), FHR-3 (C), FHR-4 (D), and FHR-5 (E) are presented together with the corresponding traditional epidemiologic odds ratio (OR) estimates obtained from logistic-regression models (352 individuals with advanced AMD and 252 control individuals from the Cambridge AMD study). The Mendelian-randomization estimates were obtained from the Wald ratio (if a single instrument was available: FHR-1, FHR-2, FHR-4, and FHR-5) or the inverse-variance weighted (IVW) method under a fixed-effect model (if multiple instruments were available: FHR-3). Raw data used for calculation of the Mendelian-randomization estimates are provided in Table 2. The proportion of the variance of each protein (as R^2^) explained by its genetic instrument(s) is indicated in the note at the bottom of each plot.

We used the Mendelian-randomization approach to test whether genetically proxied FHR concentrations are associated with risk of AMD. Table 2 reports details of the IVs (genetic variants) selected via the p-value-clumping method with an LD cut-off R^2^ < 0.001 and the 1000 Genomes Project EUR population reference. When we used an LD cut-off of R^2^ < 0.01, the same IVs were selected. Figure 5 shows the Mendelian-randomization estimates of the FHR concentrations obtained via the one-sample and two-sample Wald ratio (if a single IV was selected: FHR-1, FHR-2, FHR-4, or FHR-5) or IVW method (if multiple IVs were selected: FHR-3) together with the traditional epidemiologic estimates of the association of the protein concentrations with AMD as obtained from logistic-regression models and ORs (Table 1). The variance of the FHR concentrations explained by each single genetic instrument varied from 0.11 to 0.53 (Table 2).

The Mendelian-randomization estimates were statistically significant and of concordant direction with the observational OR estimates for FHR-1, FHR-2, FHR-4, and FHR-5, providing evidence in support of a causal effect (Figure 5). We observed overlapping CIs for the one-sample and the two-sample Mendelian-randomization estimates; the latter showed higher accuracy with much narrower CIs, likely reflecting the larger dataset used for estimating the genetic associations with the risk of AMD (i.e., IAMDGC GWAS^2^). For FHR-3, the IV at the *CFH* locus was the only one that showed a significant association with AMD risk, but the direction of allelic effect on protein concentrations was discordant from that on disease. The corresponding Mendelian-randomization estimate did not support an association of FHR-3 with the disease (one-sample: 0.99, 95% CI = 0.93–1.06, I^2^ = 0%; two-sample: 0.99, 95% CI = 0.98–1.01, I^2^ = 57%). The GWAS of FHL-1 did not show any genome-wide-significant signals that could be used as genetic instruments in the Mendelian-randomization analysis. It is worth noticing that the strongest FHL-1 GWAS signal at the *CFH* locus was observed at rs61820755 (p = 5.3 × 10^−6^, β = 0.22) and that this variant did not show association with AMD in the Cambridge AMD study (p = 0.74; β = 0.05) or in the IAMDGC study^2^ (p = 0.50; β = −0.02).

Finally, we repeated the IV selection by using the GCTA-COJO^31^ approach with the available individual-level genotype data from the entire control set genotyped with the same array in the Cambridge AMD study^2^^,^^26^^,^^27^ as a reference for LD estimates (n = 419). The same sets of IVs as presented in Table 2 were identified for all FHR proteins. Additional secondary signals that could be used as IVs were identified for FHR-2 (rs79351096), FHR-3 (rs16840522), and FHR-4 (rs34538561) at the *CFH* locus (Table S7). These additional signals are not in high LD with each other or with the primary FHR signals (Table 2), except for rs16840522 of FHR-3 with the top signal of FHR-1 rs149369377 (R^2^ = 0.96, D′ = 0.99). The corresponding Mendelian-randomization estimates for FHR-2 (one-sample: 1.58, 95% CI = 1.39–1.80; two-sample: 1.46, 95% CI = 1.42–1.50) overlapped with the ones based on a single IV selected via p-value clumping (Figure 5), although heterogeneity was observed (I^2^ = 66% and I^2^ = 94% for the one-sample and the two-sample estimates, respectively). The Mendelian-randomization estimate for FHR-3 became significant (one-sample: 1.07, 95% CI = 1.01–1.13; two-sample: 1.08, 95% CI = 1.07–1.09) and showed high heterogeneity (I^2^ = 80% and I^2^ = 99% for the one-sample and the two-sample estimates, respectively). The Mendelian-randomization estimate for FHR-4 was refined to 1.19, 95% CI = 1.07–1.32 (one-sample) and 1.10, 95% CI = 1.08–1.12 (two-sample) and also showed high heterogeneity (I^2^ = 98% and I^2^ = 99% for the one-sample and the two-sample estimates, respectively). The Mendelian-randomization estimates calculated from secondary signals should be interpreted with caution given that our GWASs of FHR protein concentrations (n = 252) might have relatively moderate or small power for dissecting independent secondary signals with accuracy.

## Discussion

This study adds compelling evidence that genetically driven elevated circulating concentrations of FHR proteins are strongly associated with AMD. Earlier genetic studies identified a common deletion of *CFHR1* and *CFHR3* and a rare deletion encompassing *CFHR1* and *CFHR4* as being protective against AMD.^16^, ^17^, ^18^, ^19^, ^20^, ^21^, ^22^ The mechanism behind these protective associations has been assumed to revolve around the FHR proteins’ being complement activators.^25^ More direct evidence that FHR proteins drive complement activation in AMD came from the discovery that increased circulating concentrations of FHR-4 in AMD-affected individuals are driven by known *CFH*-locus AMD-risk variants.^24^ The protein itself was shown to accumulate in the intercapillary septa of human eyes, the primary site of complement over-activation associated with AMD.^34^ In the study reported here, we show that in fact the concentrations of all five circulating FHR proteins are elevated in advanced cases of AMD (Table 1, Figure 2). Furthermore, by developing a unique mass-spectrometry-based measuring technique, we were able to measure for the first time both *CFH* splice variants: FH and FHL-1. Our data confirmed previous findings that circulating FH concentrations do not change with disease.^24^ Despite the lack of differing FH concentrations, we observed that circulating concentrations of FHL-1 were statistically elevated in AMD-affected individuals (p = 4.9 × 10^−4^) (Table 1, Figure 2). However, we did not find that any genome-wide-significant signals from the GWAS of FHL-1 in 252 controls (Figure S6, Table S5) could serve as genetic instruments in our Mendelian-randomization analysis. As such, the elevation of FHL-1 concentrations in advanced cases of AMD remains observational.

The molecular mechanisms underpinning the genetic AMD risk carried on chromosomal region 1q31.3, and indeed how it contributes to complement over-activation, have been widely debated. Some genetic risk variants have obvious effects, such as the FH and FHL-1 polymorphism p.Tyr402His, which reduces their binding to the extracellular matrix in the choriocapillaris and thus leads to less support for the degradation of C3b.^35^^,^^36^ However, the role of non-coding AMD-risk variants in the *CFH* locus has been much harder to dissect, and an assumption that they somehow alter the expression or function of FH (or FHL-1) itself has prevailed. Indeed, given the previous inability to simultaneously measure concentrations of the five FHR proteins, FH, and FHL-1, this has until now remained unchallenged.

Mass spectrometry provides high levels of specificity for protein quantitation because proteins (and proteotypic peptides) are identified accurately by their mass and fragmentation patterns. This makes mass spectrometry ideally suited to the detection of protein splice variants and isoforms, and in this case to the analysis of FHL-1, which differs from FH only via a unique 4 amino acid N-terminal sequence. Here we can access an FHL-1 proteotypic peptide by using an alternative protease, GluC, to allow detection of FHL-1 and accurate measurement of its concentrations in the circulation. The multiplex nature of mass spectrometry also allows additional proteins to be added to the same assay, such that we can identify all seven key proteins encoded at the RCA locus in a single experiment. The addition of stable-isotope-labeled peptides subsequently allows quantitation, which we show here to be stable and precise across many hundreds of AMD samples, providing a powerful tool for the study of FH, FHL-1, and FHR-1 through FHR-5 in complement regulation. This approach has been tried previously^37^ with a standard trypsin enzyme for proteolysis; this allows for measurement of FHR proteins, but not FHL-1. The concentrations of FHR proteins reported here are similar (within 2x) to those reported by Zhang et al.^37^ Mean protein concentrations determined in our assay are slightly lower, with the exception of FHR-3, where we report mean concentrations in control samples of 24.1 nM, or 0.9 μg/mL, versus 0.02 μg/mL reported by Zhang et al.,^37^ and FHR-5, where we report mean control concentrations of 25.5 nM, or 1.6 μg/mL, versus 5.5 μg/mL, possibly because Zhang et al.^37^ used different fragment ion transitions for the endogenous peptides and their equivalent heavy-labeled standards.

AMD represents a paradigm in the field of complex genetics since the seminal discovery in 2005 of the *CFH* as a major susceptibility gene.^8^, ^9^, ^10^, ^11^ With this study we continued to dissect the role of the *CFH* locus in AMD, beyond FH. The FH, FHL-1, and FHR-1 through FHR-5 proteins are mainly synthesized in the liver (Figure S1), so measurement of circulating concentrations in plasma allows exploration of the effects of non-coding *CFH* variants that are strongly associated with AMD risk.^2^ Using 252 non-AMD controls to get insights into the genetic determinants of the circulating protein concentrations measured in this study, we discovered that genome-wide-significantly associated variants in our analyses of the FHR protein concentrations overlap with the AMD-associated *CFH* region; Figure 3). Established genetic associations with AMD risk at the non-coding variants 1.1, proxy for p.Tyr402His 1.2, and 1.6 translated into genome-wide-significant associations with concentrations of FHR-1, FHR-2, FHR-3, and FHR-4 from the GWASs in our control group (Table 3, Figure 4).

The identification of the *CFH* locus as a *cis* protein quantitative-trait locus (*cis*-pQTL) associated with concentrations of the five FHR proteins prompted us to use the available genetic data in a Mendelian-randomization fashion to triangulate this evidence. Mendelian randomization is increasingly being used because it can overcome major limitations such as unmeasured confounding and/or reverse causality in studies of the relationship between a modifiable exposure and a disease outcome or trait, and it is becoming a standard for assessing new drug targets.^38^^,^^39^ For FHR-3 the Mendelian-randomization approach suggests that the association with AMD as estimated by a traditional observational OR (Table 1) might have arisen from residual confounding and/or reverse causality (Figure 5). For FHR-1, FHR-2, FHR-4, and FHR-5, on the other hand, the statistical support provided by the univariate Mendelian-randomization analyses for a potential casual role in susceptibility to AMD is striking, and Mendelian-randomization estimates corroborate the preliminary evidence shown by the observational OR estimates (Table 1, Figure 5). This finding reframes our understanding of the etiology of AMD and the role of the non-coding risk variants on chromosome 1q31.3, demonstrating that the FHR proteins play a prominent role that requires significant further research.

Among the methodological approaches that use genetic data to assess relationships between risk factors and outcomes, Mendelian randomization is the only one that directly assesses the causal effect of a risk factor on an outcome.^40^ Nevertheless, there are still questions that remain unanswered by the present study and could be addressed through the use of recently developed analytical tools to perform, for example, multivariate analyses of the FHR protein concentrations as well as conditional and genetic colocalization analyses.^41^, ^42^, ^43^, ^44^, ^45^ At present, given the relatively moderate statistical power of our study, we did not fully disentangle the relative role of the concentration of each FHR protein on AMD risk and/or narrowing down the specific *CFH* genes and genetic variants that are likely to be involved in the causal cascade with AMD. There was limited pairwise correlation between the FHR protein concentrations in controls (Figure 2), and the GWAS signals of the FHR protein concentrations are not in high LD with each other, except for the top signals of FHR-2 and FHR-4 (R^2^ = 0.83, D′ = 0.95, Table S6) and for both the top signal of FHR-1 and the secondary signal of FHR-3 (R^2^ = 0.96, D′ = 0.99). As such, whether each of the FHR proteins that showed a strong association with advanced AMD coupled with a significant Mendelian-randomization estimate is independently causal to AMD needs further investigation. Moreover, the top GWAS signals of the FHR protein concentrations showed low to modest LD with AMD-risk variants 1.1, 1.2, 1.4, and 1.6 (Table S6). Although this might suggest a modest genetic colocalization between the corresponding FHR protein concentrations and AMD, it is worth noting that also the top GWAS signal of FHR-4 in our previously published study on the Cambridge samples^24^ showed only modest LD with the top AMD-associated variant rs10922109 [1.1] (rs61818890, R^2^ = 0.49). However, when the Cambridge results were meta-analyzed with data from a second cohort (EUGENDA), the top GWAS signal of FHR-4 was found to be in high LD with 1.1 (rs10737680, R^2^ = 0.98). We expect that once FHR protein concentrations are available for larger datasets, new high-powered analyses will help clarify further questions that currently remain unanswered by our study.

Finally, the data presented in this study additionally highlight the targeting (and lowering) of FHR proteins in the circulation as a viable therapeutic avenue for AMD. Indeed, delivery of a systemic therapeutic provides evidence that a paradigm in ocular therapeutic strategies could allow affected individuals to avoid surgical procedures, especially in the early stages of disease before the loss of visual acuity, where therapeutic intervention might yield the most benefit. AMD individual stratification would be important because only a proportion of AMD-affected individuals are likely to suffer from FHR-mediated disease. However, as demonstrated here, an AMD-affected individual’s genetic-risk profile, coupled with measurements of their circulating FHR protein concentrations, could possibly be used in the future to identify and stratify those affected people most likely to benefit from such treatments and to monitor their response to FHR-lowering agents.

## Data and code availability

The mass-spectrometry data are available via the ProteomeXchange data repository under accession number PXD023466. The Genotype-Tissue Expression (GTEx) Project^46^ dataset used for the gene expression analyses was obtained from the GTEx portal, dataset dbGaP accession number dbGaP: phs000424.v8.p2 (GTEx Analysis Release V8); the GTEx Project was supported by the Common Fund of the Office of the Director of the National Institutes of Health and by NCI, NHGRI, NHLBI, NIDA, NIMH and NINDS.

## Web resources

EPACTS, http://genome.sph.umich.edu/wiki/EPACTS
GTEx portal, https://gtexportal.org/home/
IAMDGC, http://eaglep.case.edu/iamdgc_web
LDlink, https://ldlink.nci.nih.gov/
PanoramaWeb, https://panoramaweb.org/
ProteomeXchange, http://proteomecentral.proteomexchange.org
Skyline, www.skyline.ms

## Supplemental information

Document S1. Figures S1–S6, Tables S1, S2, and S7, and a list of consortium members

Table S3. Reproducibility of experimental triplicate samples

Table S4. Raw individual-level concentrations of FH, FHL-1, and FHR-1 to FHR-5 in plasma samples from the Cambridge AMD cohort

Table S5. Top 10,000 association signals from the GWASs of FH, FHL-1, FHR-1, FHR-2, FHR-3, FHR-4, and FHR-5 protein concentrations in 252 controls from the Cambridge AMD cohort

Table S6. Linkage disequilibrium (R2) for the variants showing the top association signals in the GWASs of the FHR-1, FHR-2, FHR-3, FHR-4, and FHR-5 concentrations in 252 Cambridge controls and the top association signals reported at the CFH locus by the International AMD Genomics Consortium study

Document S2. Article plus Document S1
