# Supplementary material for: Beyond factor H: The impact of genetic-risk variants for age-related macular degeneration on circulating factor-H-like 1 and factor-H-related protein concentrations
Source: Am J Hum Genet. 2021 Jul 13;108(8):1385–400. doi: 10.1016/j.ajhg.2021.05.015 (PMC8387294; doi:10.1016/j.ajhg.2021.05.015)
Supplement: Document S1. Figures S1–S6, Tables S1, S2, and S7, and a list of consortium members [file mmc1.pdf]

The American Journal of Human Genetics, Volume *108*

Supplemental information

Beyond factor H: The impact of genetic-risk variants for age-related macular degeneration on circulating factor-H-like 1 and factor-H-related protein concentrations

Valentina Cipriani, Anna Tierney, John R. Griffiths, Verena Zuber, Panagiotis I. Sergouniotis, John R.W. Yates, Anthony T. Moore, Paul N. Bishop, Simon J. Clark, and Richard D. Unwin

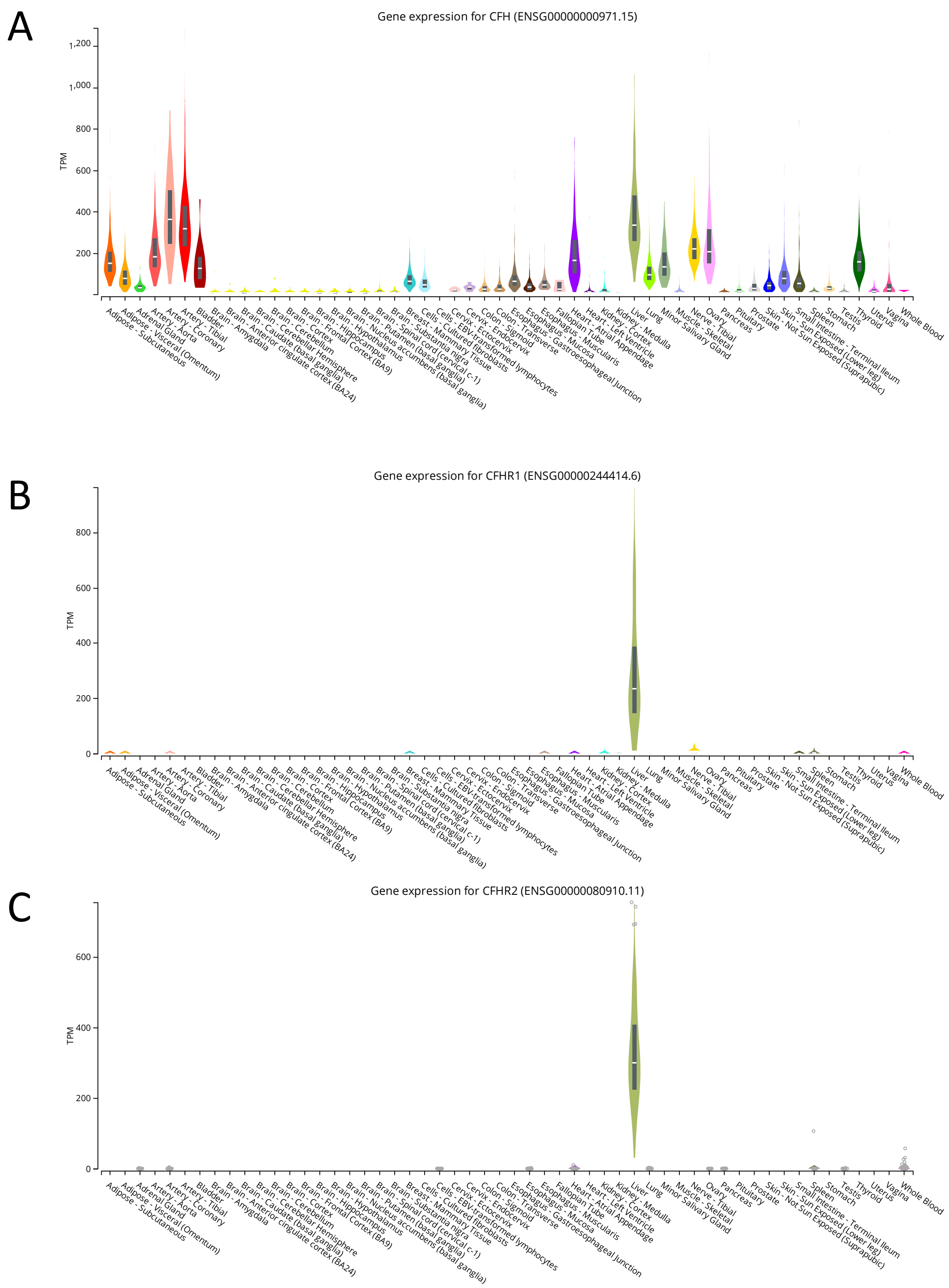

Figure S1. Gene expression of *CFHR1*, *CFHR2*, *CFHR3*, *CFHR4* and *CFHR5* is restricted to liver.

RNA sequencing of 54 human tissue samples from the Genotype-Tissue Expression (GTEx) project[1] (https://gtexportal.org/home/multiGeneQueryPage; dataset dbGaP accession number phs000424.v8.p2) detected *CFHR1* (B), *CFHR2* (C), *CFHR3* (D), *CFHR4* (E) and *CFHR5* (F) expression exclusively in the liver, whereas *CFH* (A) expression was more widespread.

(continued on the next page)

D

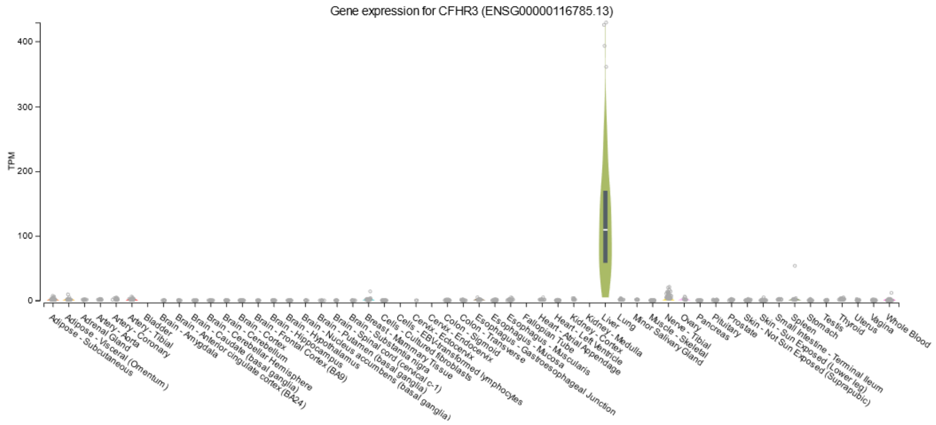
Gene expression for CFHR3 (ENSG00000116785.13)
TPM
400
300
200
100
0

E

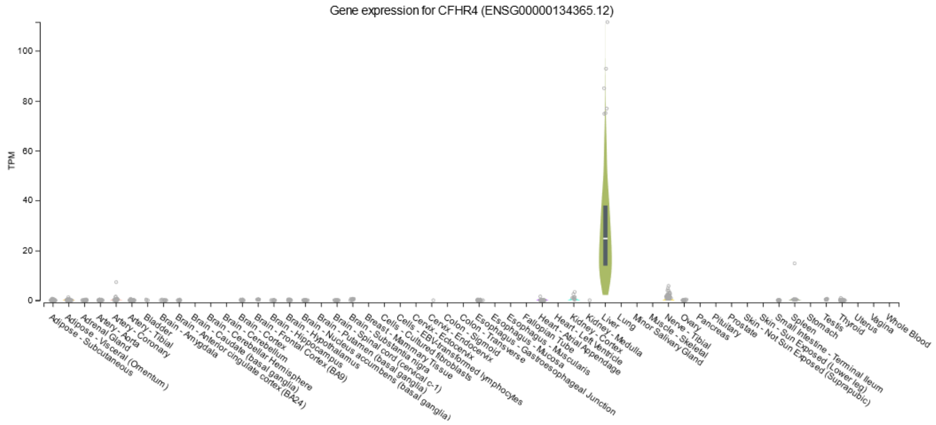
Gene expression for CFHR4 (ENSG00000134365.12)
TPM
100
80
60
40
20
0

F

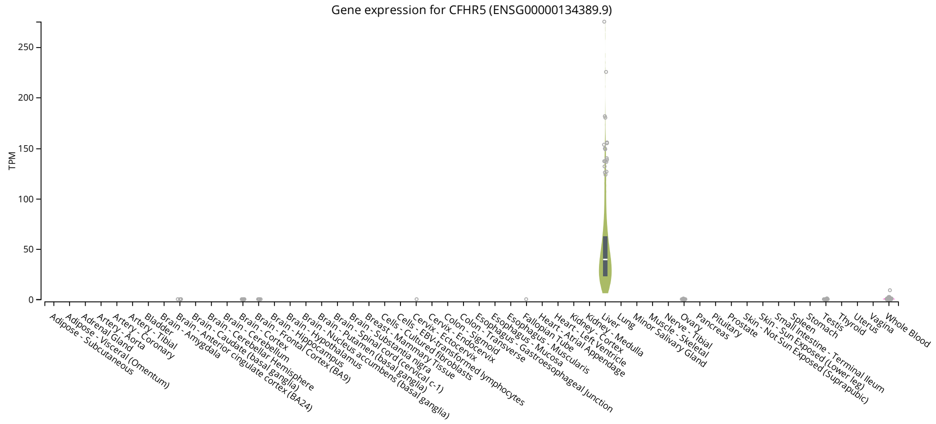
Gene expression for CFHR5 (ENSG00000134389.9)
TPM
250
200
150
100
50
0

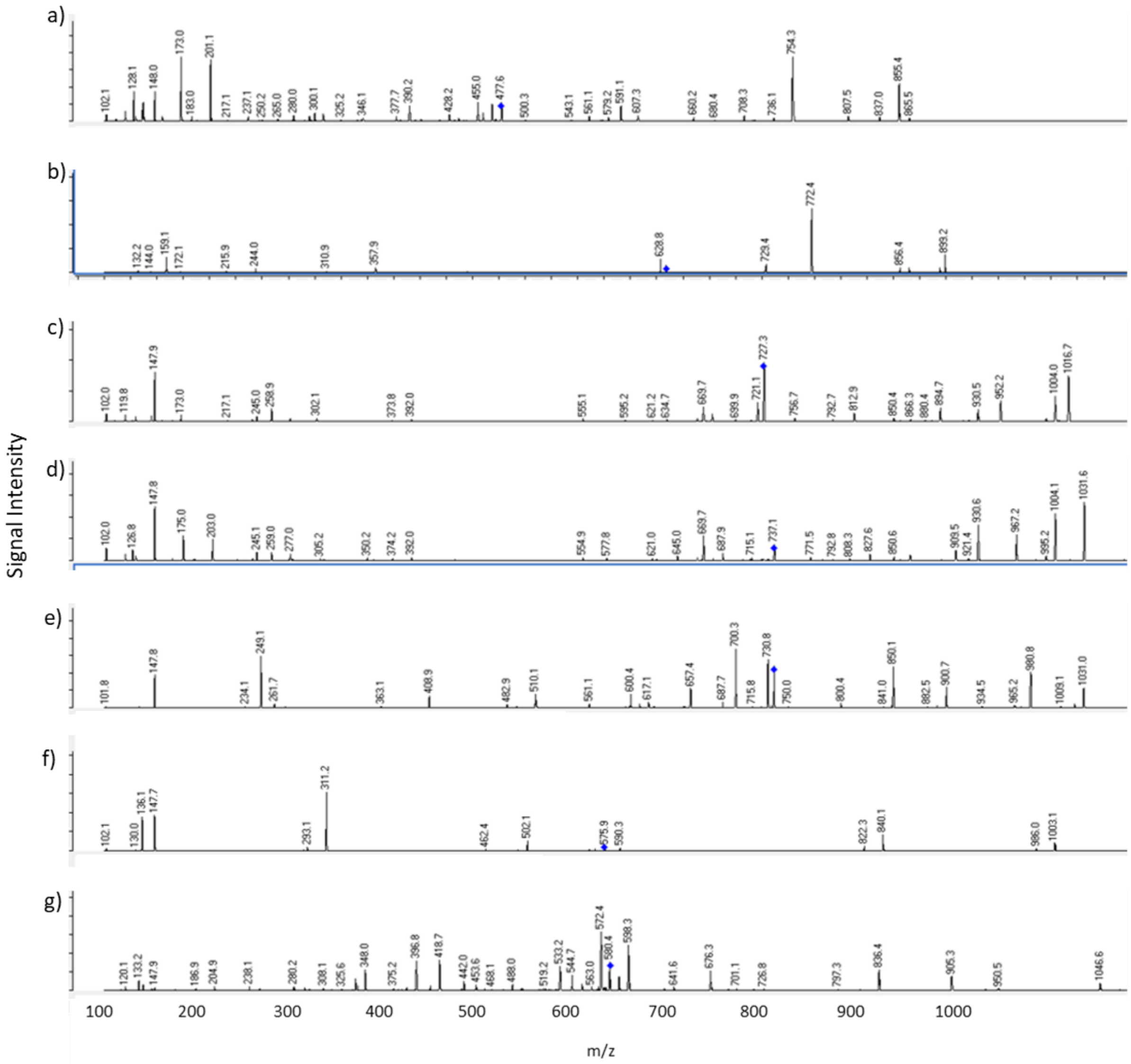

Figure S2. MS/MS fragmentation spectra of all proteotypic peptides used for quantification of FH, FHL-1 and FHR-1 to FHR-5 proteins in human samples.

a) VTYKcFE (FH), b) NGWSPTPRcIRVSFTL (FHL-1), c) ATFcDFPKINHGILYDEE (FHR-1), d) AMFcDFPKINHGILYDEE (FHR-2), e) VAcHPGYGLPKAQTTVTcTE (FHR-3), f) YQcQSYYE (FHR-4), and g) RGWSTPPIcSFTKGE (FHR-5).

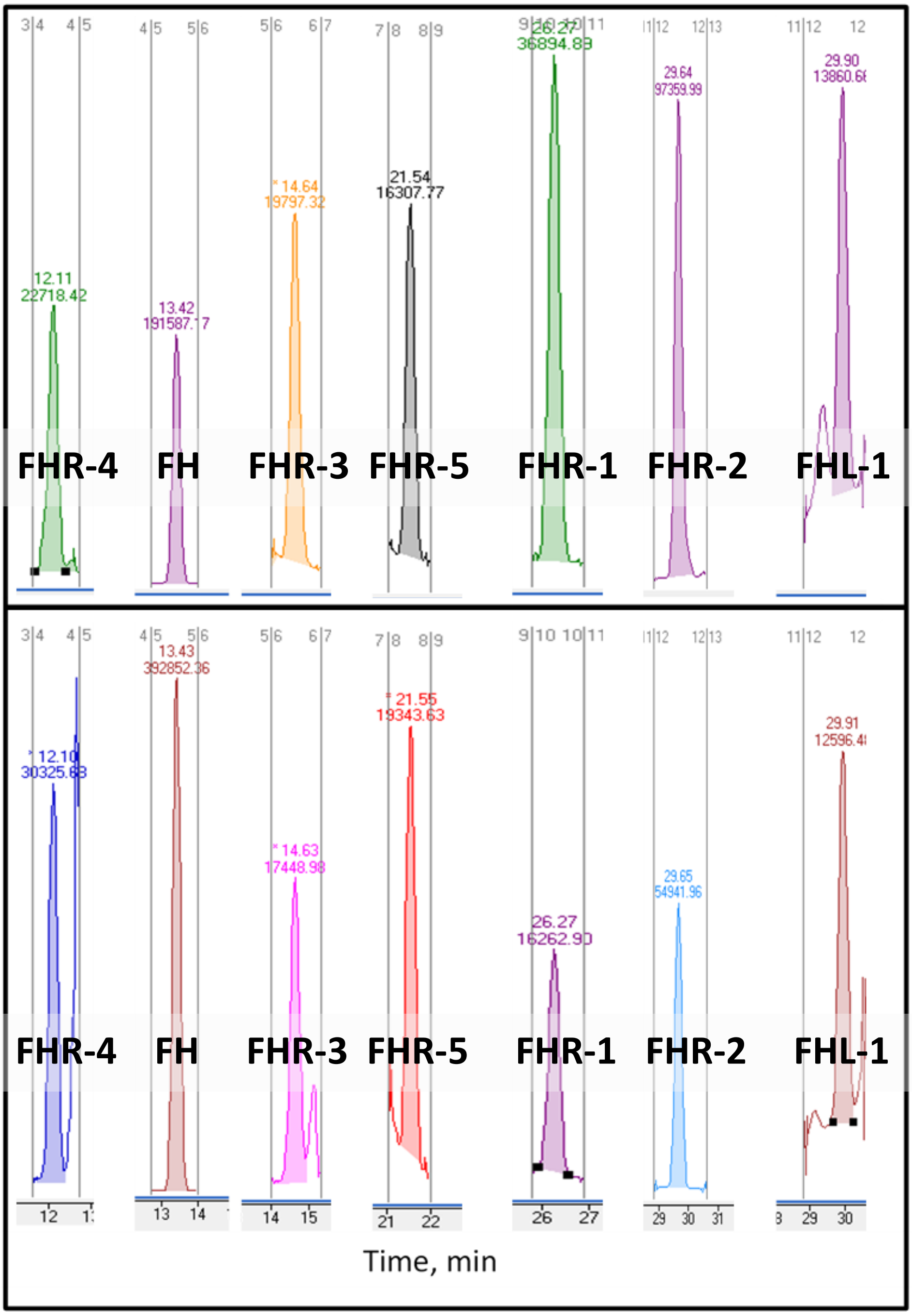

Figure S3. Overlay of endogenous and stable isotope-labelled standard peptide SRM signals.

To confirm assay specificity, stable isotope-labelled peptides were spiked into plasma and the elution profiles of each of the heavy:light pairs was compared to confirm specificity of the individual SRMs for each peptide. Upper panel shows signals from endogenous peptides, while the lower panel shows the equivalent SIS peptide.

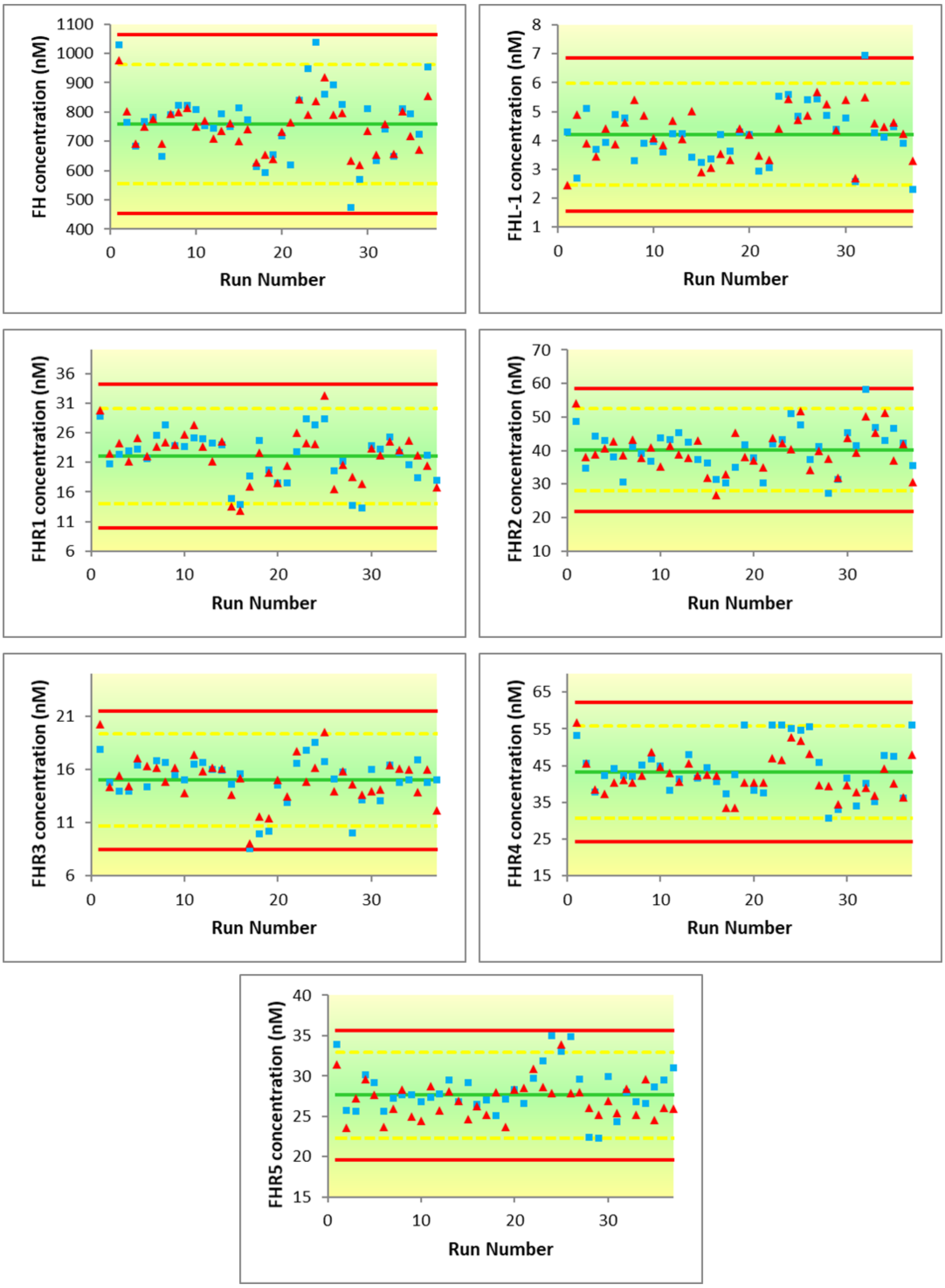

Figure S4. Levy-Jennings graphs to monitor between-batch stability of the whole process across the course of the study.

Measured concentrations for each protein in two replicate analyses of the same sample included in each batch were monitored. Green line = mean concentration, Yellow line = +/- 2x s.d., Red line = +/-3x s.d.

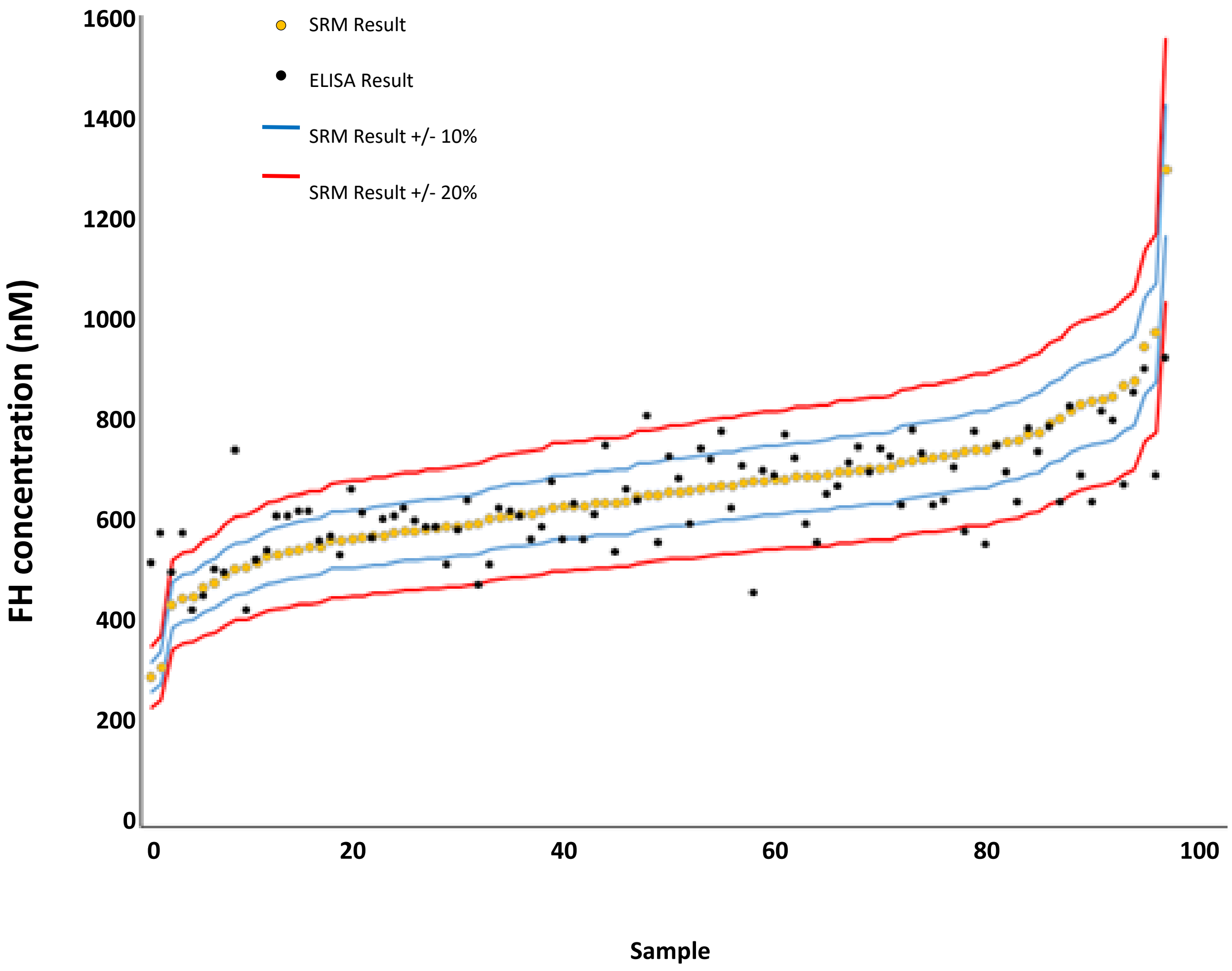

Figure S5. Correlation between measured concentrations of FH using the LS-MS based assay and immunoassay.

Immunoassay-derived concentrations were normalised to match the median concentration calculated by the SRM. In most case ELISA measurements are within 20% of the SRM measurement.

A

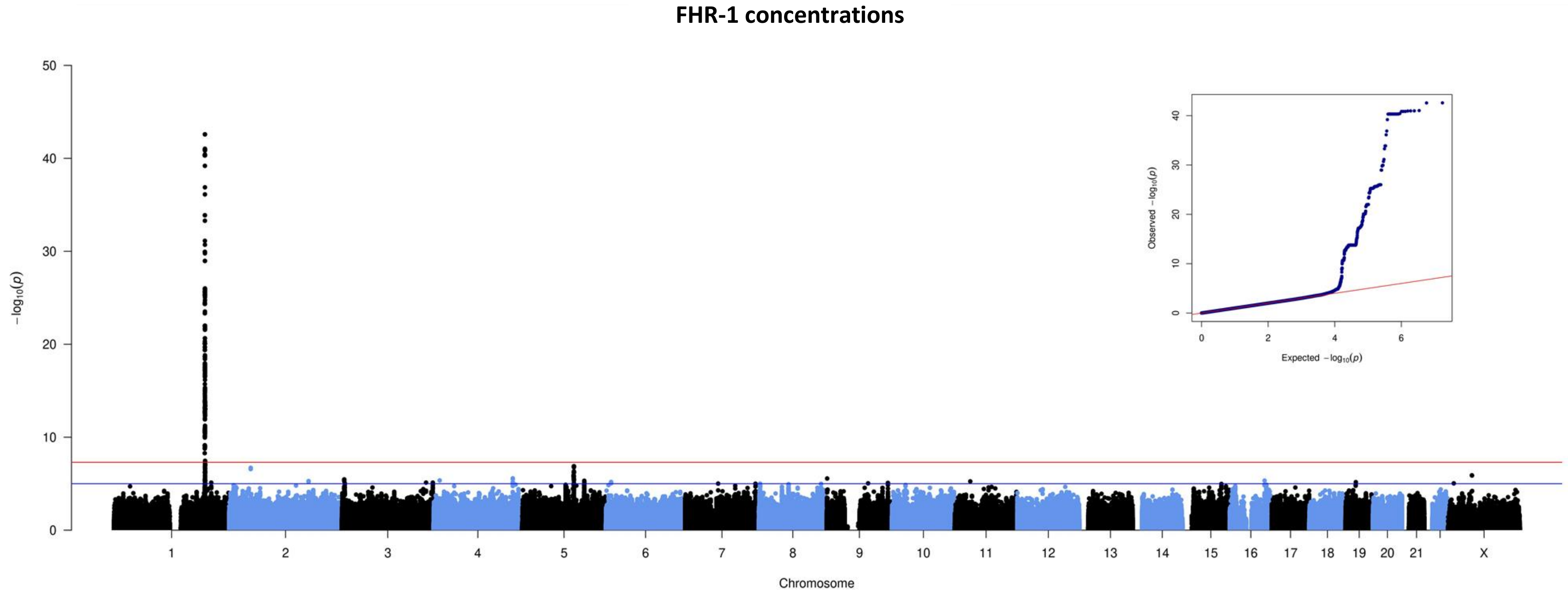

Figure S6. GWASs of circulating FHR-1, FHR-2, FHR-3, FHR-4, FHR-5 protein concentrations in 252 controls from the Cambridge AMD cohort reveal a strong genome-wide significant signal spanning the AMD-associated *CFH* locus on chromosome 1q31.3.

Manhattan plot together with quantile-quantile (QQ) plot (upper right-hand side of each panel) for the GWAS of FHR-1 (A), FHR-2 (B), FHR-3 (C), FHR-4 (D), FHR-5 (E), FH (F) and FHL-1 (G) protein concentrations. Manhattan plots illustrate P-values for each single variant tested for association with the protein concentrations. Observed -$\log_{10}$(P-values) are plotted against the genomic position of each variant on chromosomes 1–22. The horizontal red line indicates the threshold considered for genome-wide significance (P-value ≤ 5 x 10^{-8}). QQ plots compare the distribution of the observed test statistics with its expected distribution under the null hypothesis of no association. Genomic control values (λ)

calculated based on the 50th percentile (and 1/10th of a percentile) were equal to 1.010 (1.004), 1.014 (1.026), 0.983 (1.074), 1.012 (1.025), 0.994 (1.014), 0.991 (1.018) and 0.995 (0.998) for FHR-1, FHR-2, FHR-3, FHR-4, FHR-5, FH and FHL-1, respectively.

(continued on the next page)

B

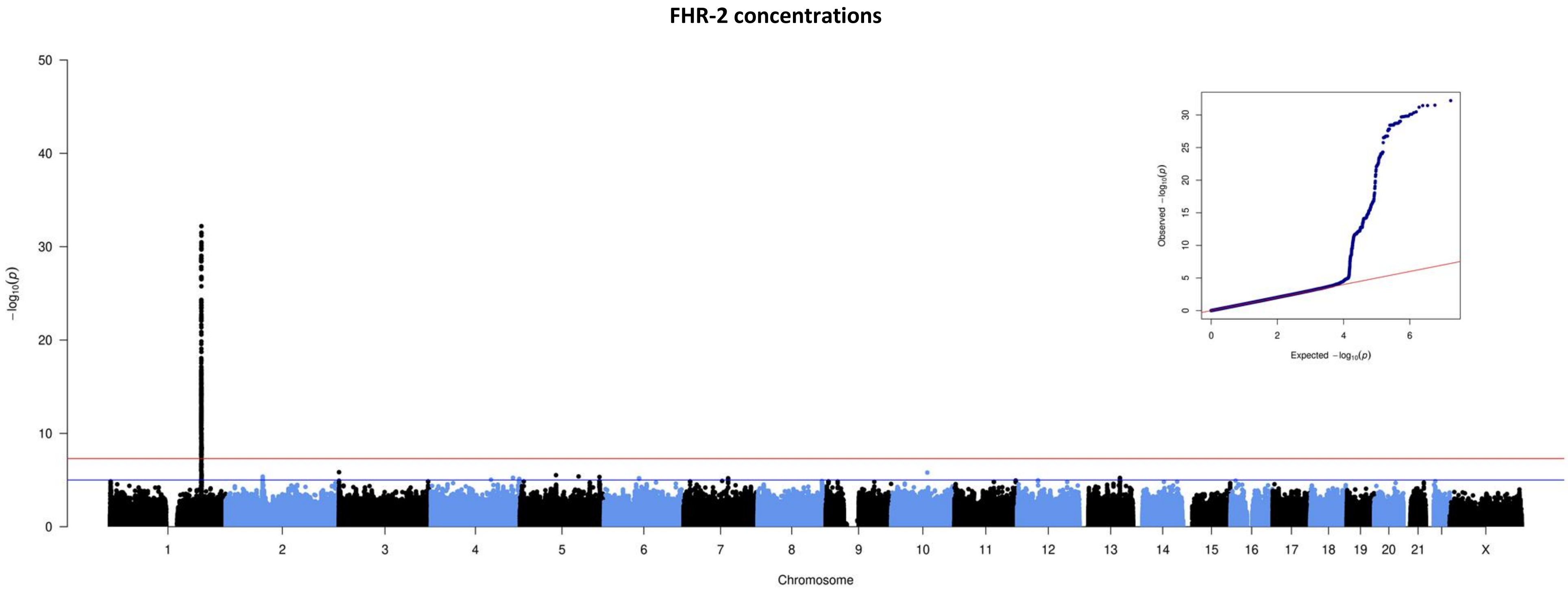

(continued on the next page)

C

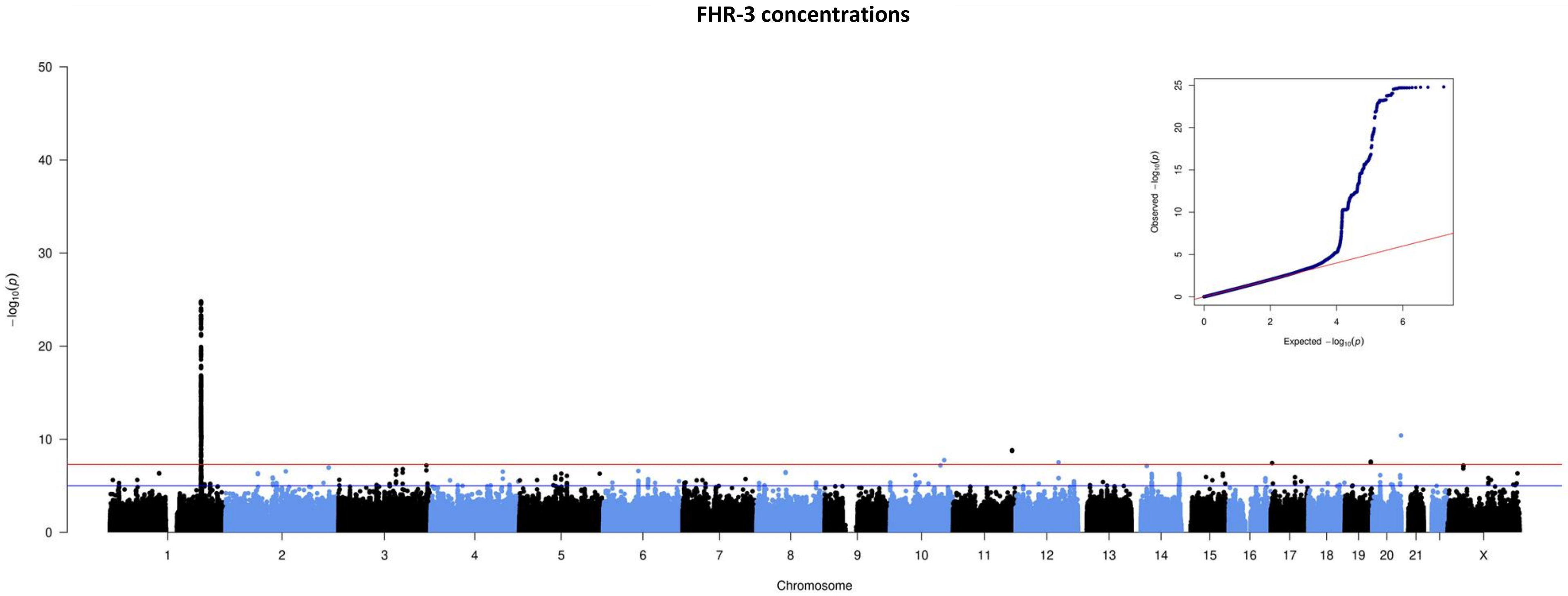

(continued on the next page)

D

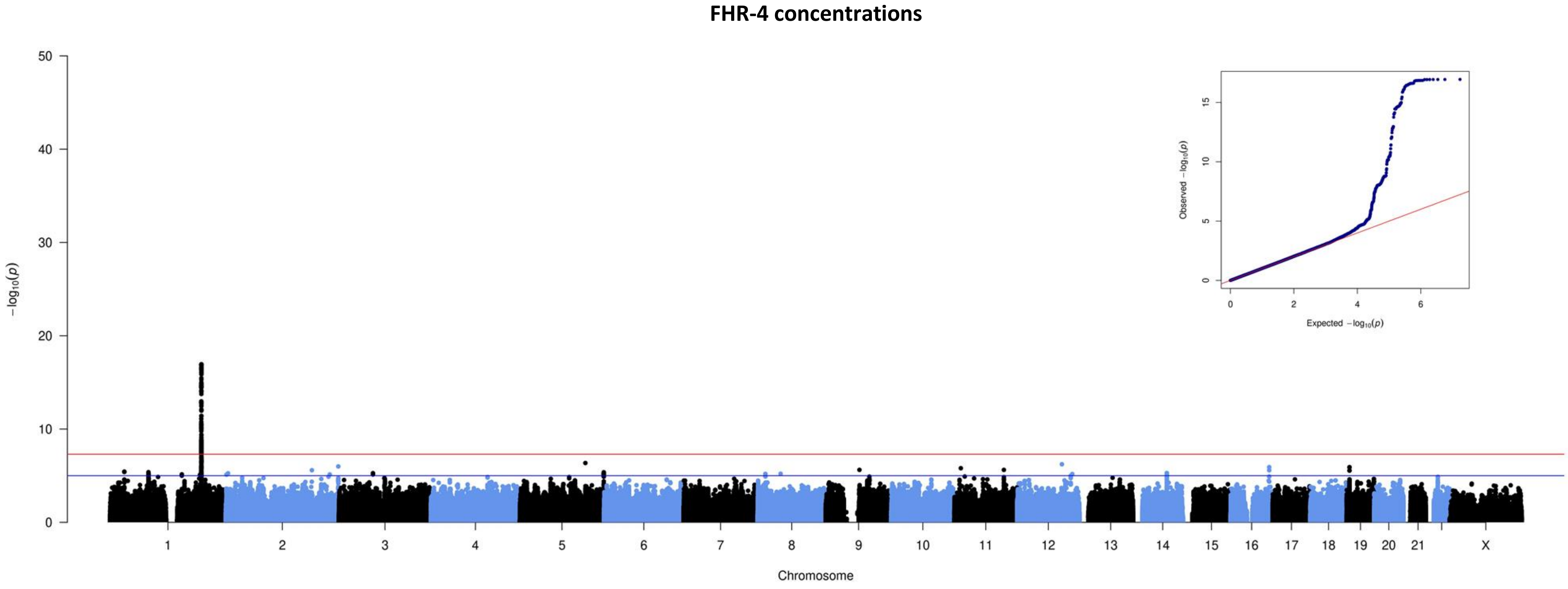

(continued on the next page)

E

FHR-5 concentrations

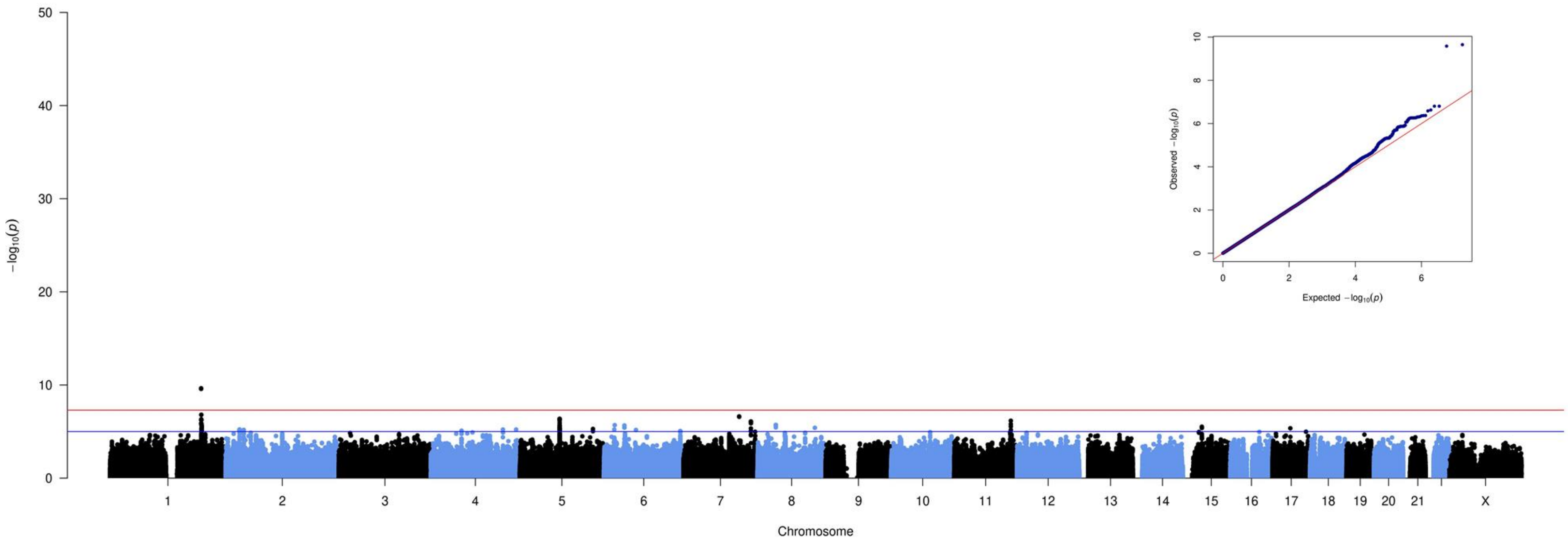

(continued on the next page)

F

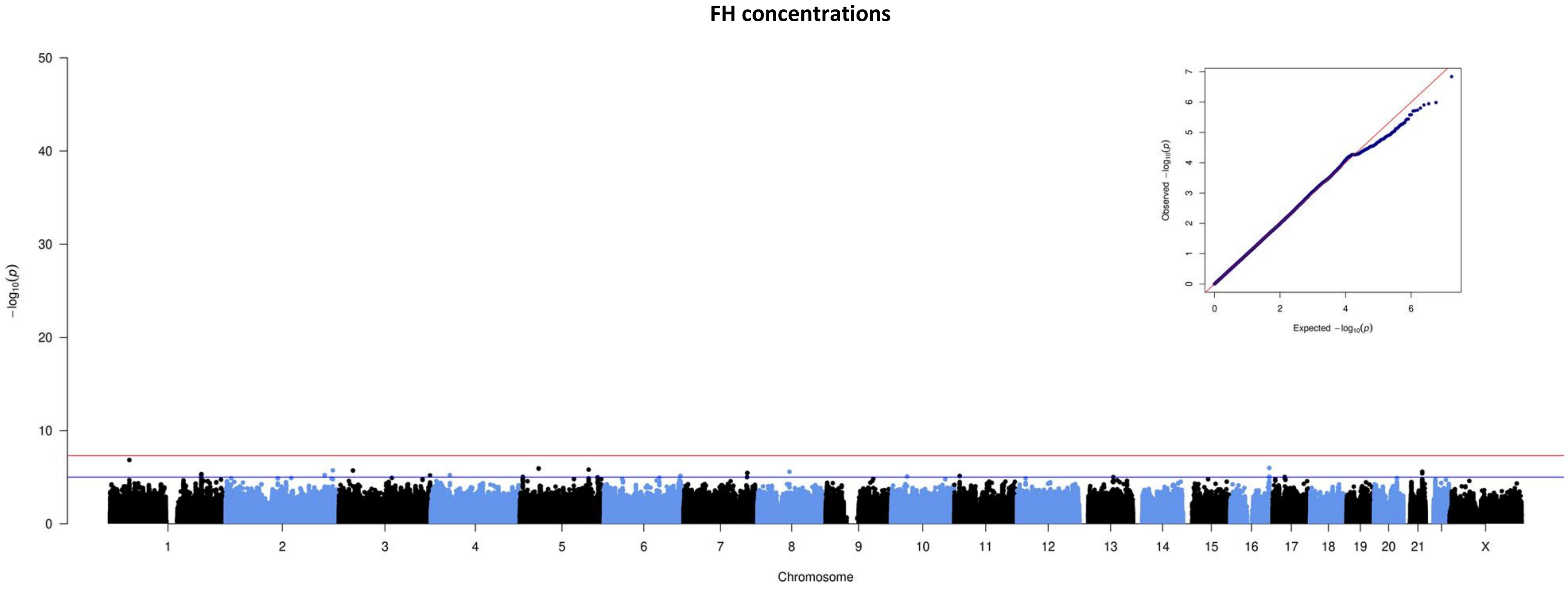

(continued on the next page)

G

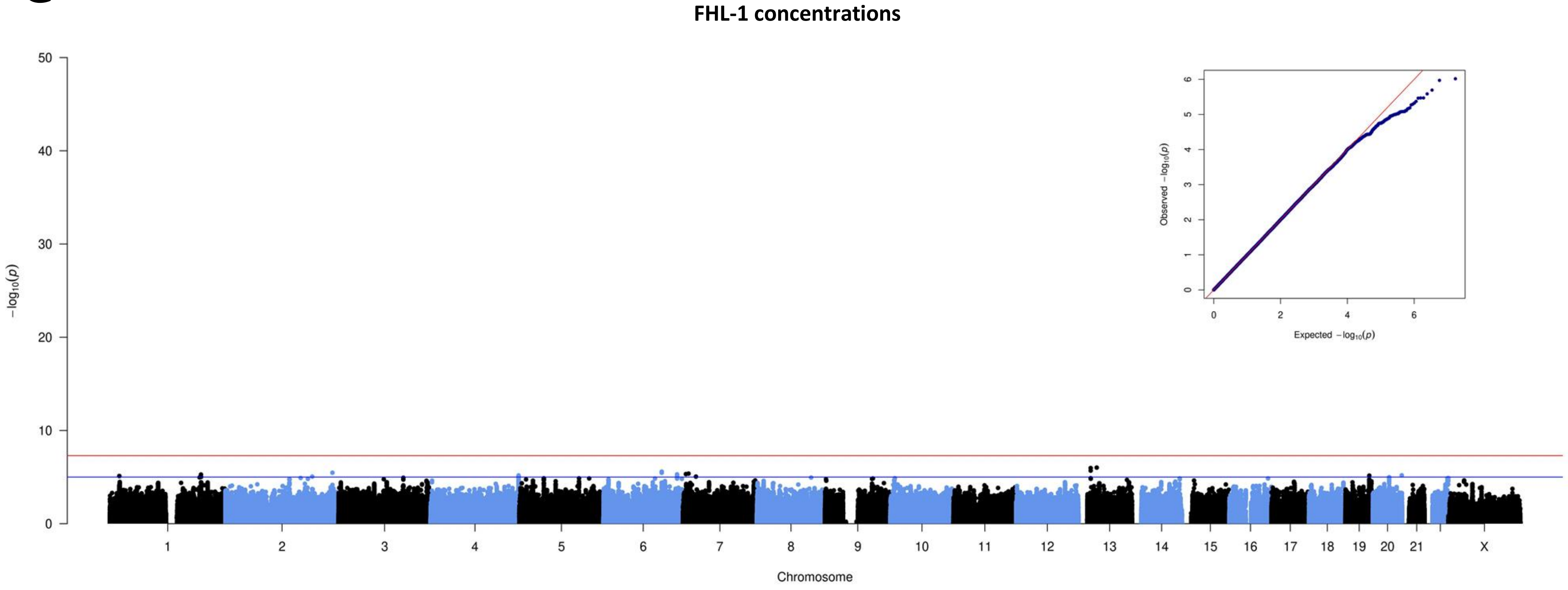
FHL-1 concentrations
50
40
30
20
10
0
$-\log_{10}(p)$
1
2
3
4
5
6
7
8
9
10
11
12
13
14
15
16
17
18
19
20
21
X
Chromosome
Observed $-\log_{10}(p)$
Expected $-\log_{10}(p)$

Parameter	Value
Gas Temp	210 °C
Gas Flow	15 l/min
Nebuliser	30 psi
Sheath Gas Temp	250 °C
Sheath Gas Flow	12 l/min
Capillary Voltage	2650 V
Nozzle Voltage	1000 V
High Pressure RF	200 V
Low Pressure RF	110 V

Table S1. Liquid chromatography-selected reaction monitoring mass spectrometry (LC-SRM-MS) instrument parameters.

**Protein**	**Peptide Sequence**	**Precursor ion m/z**	**Product ions m/z**	**Collision energy, eV**	**Dwell time, ms**
**FH**	VTY**K**cFE (Light)	473.7	583.3, 847.4, 746.3	16, 16, 16	400, 200, 150
	VTY**K**cFE (Heavy)	477.7	591.3, 855.4, 754.3	16, 16, 16	400, 200, 150
**FHL-1**	NGWSPTP**R**cIRVSFTL (Light)	631.2	723.9, 860.5, 767.4	19, 19, 19	150, 200, 100
	NGWSPTP**R**cIRVSFTL (Heavy)	634.3	728.9, 865.5, 772.4	19, 19, 19	150, 200, 100
**FHR-1**	ATFcD**F**PKINHGILYDEE (Light)	724.2	925.6, 1011.9, 947.1	20, 16, 20	400, 200, 200
	ATFcD**F**PKINHGILYDEE (Heavy)	727.2	930.6, 1016.9, 952.1	20, 16, 20	400, 200, 200
**FHR-2**	AMFcD**F**PKINHGILYDEE (Light)	734.0	999.5, 925.9, 1027	18,22,18	150, 125, 100
	AMFcD**F**PKINHGILYDEE (Heavy)	737.3	1004.5, 930.9, 1032	18,22,18	150, 125, 100
**FHR-3**	VAcHPG**Y**GLP**K**AQTTVTcTE (Light)	730.7	1022.4, 971.7	16, 18	350, 400
	VAcHPG**Y**GLP**K**AQTTVTcTE (Heavy)	736.7	1031.4, 980.7	16, 18	350, 400
**FHR-4**	**Y**QcQSYYE (Light)	570.7	830.3, 993.1, 311.1	11, 10, 14	250, 250, 250
	**Y**QcQSYYE (Heavy)	575.7	840.3, 1003.1, 311.1	11, 10, 14	250, 250, 250
**FHR-5**	**R**GWSTPPIcSFT**K**GE (Light)	575.2	828.4, 895.5, 588.3	16, 15, 20	200, 350, 200
	**R**GWSTPPIcSFT**K**GE (Heavy)	581.2	836.4, 905.5, 598.3	16, 15, 20	200, 350, 200

Table S2. SRM transition parameters.

Protein	Instrumental variable (IV) dbSNP ID (Chr:Position)[a] Non effect allele/Effect allele	*cis* / *trans* pQTL	IV strength (R^2)[b]	Association with protein concentrations in 252 Cambridge AMD study[2; 3] controls			Association with AMD in the Cambridge AMD GWAS[2; 3] (845 AMD cases and 419 controls)			Association with AMD in the IAMDGC GWAS[4] (16,144 AMD cases and 17,832 controls)			
				Beta	SE	P-value	Beta	SE	P-value	Beta	SE	P-value	Minor Allele Frequency
**FHR-2**	rs79351096 1:196918741_G/A (*CFHR2* nonsynonymous)	*cis*	0.09	-1.81	0.36	1.2×10^{-6}	-0.37	0.29	0.207	-0.46	0.06	1.0×10^{-13}	0.019
**FHR-3**	rs16840522 1:196710916_T/C (*CFH* intronic)	*cis*	0.35	-1.79	0.16	6.1×10^{-24}	-0.74	0.125	4.6×10^{-9}	-0.86	0.025	5.6×10^{-292}	0.158
**FHR-4**	rs34538561 1:196534406_C/G (*KCNT2* intronic)	*cis*	0.12	-1.63	0.28	2.5×10^{-8}	0.56	0.14	4.0×10^{-5}	0.51	0.03	7.8×10^{-92}	0.132

Table S7. Additional instrumental variables (IVs) for FHR-2, FHR-3 and FHR-4 identified at the *CFH* locus using the GCTA-COJO[5] approach.

[a]Chromosomal position is given according to the NCBI RefSeq hg19 human genome reference assembly; [b]The strength of each IV was evaluated using R^2 as the proportion of the variance of the protein explained by the genetic variant (function *get_r_from_pn* from R package *TwoSampleMR*, version 0.5.5).

The GCTA-COJO[5] approach was applied with default settings; the available individual-level genotype data from the entire control set in the Cambridge AMD study,[2; 3] n = 419, was used as a reference sample to estimate LD among genetic variants.

AMD = Age-Related macular degeneration; GWAS = Genome-wide association study; IAMDGC = International Age-Related Macular Degeneration Genomics Consortium; pQTL = protein quantitative trait locus.

Supplemental Note

List of the International Age-related Macular Degeneration Genomics Consortium (IAMDGC) members

The list reflects the author list of the previous IAMDGC publication by Fritsche *et al.*, 2016.[4]

Lars G Fritsche[1], Wilmar Igl[2], Jessica N Cooke Bailey[3], Felix Grassmann[4], Sebanti Sengupta[1], Jennifer L Bragg-Gresham[1,5], Kathryn P Burdon[6], Scott J Hebbring[7], Cindy Wen[8], Mathias Gorski[2], Ivana K Kim[9], David Cho[10], Donald Zack[11-15], Eric Souied[16], Hendrik P N Scholl[11,17], Elisa Bala[18], Kristine E Lee[19], David J Hunter[20,21], Rebecca J Sardell[22], Paul Mitchell[23], Joanna E Merriam[24], Valentina Cipriani[25,26], Joshua D Hoffman[27], Tina Schick[28], Yara T E Lechanteur[29], Robyn H Guymer[30], Matthew P Johnson[31], Yingda Jiang[32], Chloe M Stanton[33], Gabriëlle H S Buitendijk[34,35], Xiaowei Zhan[1,36,37], Alan M Kwong[1], Alexis Boleda[38], Matthew Brooks[38], Linn Gieser[38], Rinki Ratnapriya[38], Kari E Branham[39], Johanna R Foerster[1], John R Heckenlively[39], Mohammad I Othman[39], Brendan J Vote[6], Helena Hai Liang[30], Emmanuelle Souzeau[40], Ian L McAllister[41], Timothy Isaacs[41], Janette Hall[40], Stewart Lake[40], David A Mackey[6,30,41], Ian J Constable[41], Jamie E Craig[40], Terrie E Kitchner[7], Zhenglin Yang[42,43], Zhiguang Su[44], Hongrong Luo[8], Daniel Chen[8], Hong Ouyang[8], Ken Flagg[8], Danni Lin[8], Guanping Mao[8], Henry Ferreyra[8], Klaus Stark[2], Claudia N von Strachwitz[45], Armin Wolf[46], Caroline Brandl[2,4,47], Guenther Rudolph[46], Matthias Olden[2], Margaux A Morrison[48], Denise J Morgan[48], Matthew Schu[49-53], Jeeyun Ahn[54], Giuliana Silvestri[55], Evangelia E Tsironi[56], Kyu Hyung Park[57], Lindsay A Farrer[49-53], Anton Orlin[58], Alexander Brucker[59], Mingyao Li[60], Christine A Curcio[61], Saddek Mohand-Saïd[62-65], José-Alain Sahel[25,62-67], Isabelle Audo[62-64,68], Mustapha Benchaboune[65], Angela J Cree[69], Christina A Rennie[70], Srinivas V Goverdhan[69], Michelle Grunin[71], Shira Hagbi-Levi[71], Peter Campochiaro[11,13], Nicholas Katsanis[72-74], Frank G Holz[17], Frédéric Blond[62-64], Hélène Blanché[75], Jean-François Deleuze[75,76], Robert P Igo Jr[3], Barbara Truitt[3], Neal S Peachey[18,77], Stacy M Meuer[19], Chelsea E Myers[19], Emily L Moore[19], Ronald Klein[19], Michael A Hauser[78-80], Eric A Postel[78], Monique D Courtenay[22], Stephen G Schwartz[81], Jaclyn L Kovach[81], William K Scott[22], Gerald Liew[23], Ava G Tan[23], Bamini Gopinath[23], John C Merriam[24], R Theodore Smith[24,82], Jane C Khan[41,83,84], Humma Shahid[84,85], Anthony T Moore[25,26,86], J Allie McGrath[27], Reneé Laux[3], Milam A Brantley Jr[87], Anita

Agarwal[87], Lebriz Ersoy[28], Albert Caramoy[28], Thomas Langmann[28], Nicole T M Saksens[29], Eiko K de Jong[29], Carel B Hoyng[29], Melinda S Cain[30], Andrea J Richardson[30], Tammy M Martin[88], John Blangero[31], Daniel E Weeks[32,89], Bal Dhillon[90], Cornelia M van Duijn[35], Kimberly F Doheny[91], Jane Romm[91], Caroline C W Klaver[34,35], Caroline Hayward[33], Michael B Gorin[92,93], Michael L Klein[88], Paul N Baird[30], Anneke I den Hollander[29,94], Sascha Fauser[28], John R W Yates[25,26,84], Rando Allikmets[24,95], Jie Jin Wang[23], Debra A Schaumberg[20,96,97], Barbara E K Klein[19], Stephanie A Hagstrom[77], Itay Chowers[71], Andrew J Lotery[69], Thierry Léveillard[62-64], Kang Zhang[8,44], Murray H Brilliant[7], Alex W Hewitt[6,30,41], Anand Swaroop[38], Emily Y Chew[98], Margaret A Pericak-Vance[22], Margaret DeAngelis[48], Dwight Stambolian[10], Jonathan L Haines[3,99], Sudha K Iyengar[3], Bernhard H F Weber[4], Gonçalo R Abecasis[1] & Iris M Heid[2]

IAMDGC members' affiliations

[1]Center for Statistical Genetics, Department of Biostatistics, University of Michigan, Ann Arbor, Michigan, USA. [2]Department of Genetic Epidemiology, University of Regensburg, Regensburg, Germany. [3]Department of Epidemiology and Biostatistics, Case Western Reserve University School of Medicine, Cleveland, Ohio, USA. [4]Institute of Human Genetics, University of Regensburg, Regensburg, Germany. [5]Kidney Epidemiology and Cost Center, Department of Internal Medicine–Nephrology, University of Michigan, Ann Arbor, Michigan, USA. [6]School of Medicine, Menzies Research Institute Tasmania, University of Tasmania, Hobart, Tasmania, Australia. [7]Center for Human Genetics, Marshfield Clinic Research Foundation, Marshfield, Wisconsin, USA. [8]Department of Ophthalmology, University of California, San Diego and Veterans Affairs San Diego Health System, La Jolla, California, USA. [9]Retina Service, Massachusetts Eye and Ear, Department of Ophthalmology, Harvard Medical School, Boston, Massachusetts, USA. [10]Department of Ophthalmology, Perelman School of Medicine, University of Pennsylvania, Philadelphia, Pennsylvania, USA. [11]Department of Ophthalmology, Wilmer Eye Institute, Johns Hopkins University School of Medicine, Baltimore, Maryland, USA. [12]Department of Molecular Biology and Genetics, Johns Hopkins University School of Medicine, Baltimore, Maryland, USA. [13]Department of Neuroscience, Johns Hopkins University School of Medicine, Baltimore, Maryland, USA. [14]Institute of Genetic Medicine, Johns Hopkins University School of Medicine, Baltimore, Maryland, USA. [15]Institue de la Vision, Université Pierre et Marie Curie, Paris, France. [16]Hôpital Intercommunal de Créteil, Hôpital Henri Mondor, Université Paris Est Créteil,

Créteil, France. [17]Department of Ophthalmology, University of Bonn, Bonn, Germany. [18]Louis Stokes Cleveland Veterans Affairs Medical Center, Cleveland, Ohio, USA. [19]Department of Ophthalmology and Visual Sciences, University of Wisconsin, Madison, Wisconsin, USA. [20]Department of Epidemiology, Harvard School of Public Health, Boston, Massachusetts, USA. [21]Department of Nutrition, Harvard School of Public Health, Boston, Massachusetts, USA. [22]John P. Hussman Institute for Human Genomics, Miller School of Medicine, University of Miami, Miami, Florida, USA. [23]Centre for Vision Research, Department of Ophthalmology and Westmead Millennium Institute for Medical Research, University of Sydney, Sydney, New South Wales, Australia. [24]Department of Ophthalmology, Columbia University, New York, New York, USA. [25]University College London Institute of Ophthalmology, University College London, London, UK. [26]Moorfields Eye Hospital, London, UK. [27]Center for Human Genetics Research, Vanderbilt University Medical Center, Nashville, Tennessee, USA. [28]Department of Ophthalmology, University Hospital of Cologne, Cologne, Germany. [29]Department of Ophthalmology, Radboud University Medical Centre, Nijmegen, the Netherlands. [30]Centre for Eye Research Australia, University of Melbourne, Royal Victorian Eye and Ear Hospital, East Melbourne, Victoria, Australia. [31]South Texas Diabetes and Obesity Institute, School of Medicine, University of Texas Rio Grande Valley, Brownsville, Texas, USA. [32]Department of Biostatistics, Graduate School of Public Health, University of Pittsburgh, Pittsburgh, Pennsylvania, USA. [33]Medical Research Council (MRC) Human Genetics Unit, Institute of Genetics and Molecular Medicine, University of Edinburgh, Edinburgh, UK. [34]Department of Ophthalmology, Erasmus Medical Center, Rotterdam, the Netherlands. [35]Department of Epidemiology, Erasmus Medical Center, Rotterdam, the Netherlands. [36]Quantitative Biomedical Research Center, Department of Clinical Science, University of Texas Southwestern Medical Center, Dallas, Texas, USA. [37]Center for the Genetics of Host Defense, University of Texas Southwestern Medical Center, Dallas, Texas, USA. [38]Neurobiology, Neurodegeneration and Repair Laboratory (N-NRL), National Eye Institute, US National Institutes of Health, Bethesda, Maryland, USA. [39]Department of Ophthalmology and Visual Sciences, University of Michigan, Kellogg Eye Center, Ann Arbor, Michigan, USA. [40]Department of Ophthalmology, Flinders Medical Centre, Flinders University, Adelaide, South Australia, Australia. [41]Centre for Ophthalmology and Visual Science, Lions Eye Institute, University of Western Australia, Perth, Western Australia, Australia. [42]Sichuan Provincial Key Laboratory for Human Disease Gene Study, Hospital of the University of Electronic Science and Technology of China and Sichuan Provincial People's Hospital, Chengdu, China. [43]Sichuan Translational Medicine Hospital, Chinese Academy of

Sciences, Chengdu, China. [44]Molecular Medicine Research Center, State Key Laboratory of Biotherapy, West China Hospital, Sichuan University, Chengdu, China. [45]EyeCentre Southwest, Stuttgart, Germany. [46]University Eye Clinic, Ludwig Maximilians University, Munich, Germany. [47]Department of Ophthalmology, University Hospital Regensburg, Regensburg, Germany. [48]Department of Ophthalmology and Visual Sciences, University of Utah, Salt Lake City, Utah, USA. [49]Department of Medicine (Biomedical Genetics), Boston University Schools of Medicine and Public Health, Boston, Massachusetts, USA. [50]Department of Ophthalmology, Boston University Schools of Medicine and Public Health, Boston, Massachusetts, USA. [51]Department of Neurology, Boston University Schools of Medicine and Public Health, Boston, Massachusetts, USA. [52]Department of Epidemiology, Boston University Schools of Medicine and Public Health, Boston, Massachusetts, USA. [53]Department of Biostatistics, Boston University Schools of Medicine and Public Health, Boston, Massachusetts, USA. [54]Department of Ophthalmology, Seoul Metropolitan Government Seoul National University Boramae Medical Center, Seoul, Republic of Korea. [55]Centre for Experimental Medicine, Queen's University, Belfast, UK. [56]Department of Ophthalmology, University of Thessaly, School of Medicine, Larissa, Greece. [57]Department of Ophthalmology, Seoul National University Bundang Hospital, Seongnam, Republic of Korea. [58]Department of Ophthalmology, Weill Cornell Medical College, New York, New York, USA. [59]Scheie Eye Institute, Department of Ophthalmology, University of Pennsylvania Perelman School of Medicine, Philadelphia, Pennsylvania, USA. [60]Department of Biostatistics and Epidemiology, University of Pennsylvania Perelman School of Medicine, Philadelphia, Pennsylvania, USA. [61]Department of Ophthalmology, University of Alabama at Birmingham, Birmingham, Alabama, USA. [62]INSERM, Paris, France. [63]Institut de la Vision, Department of Genetics, Paris, France. [64]Centre National de la Recherche Scientifique (CNRS), Paris, France. [65]Centre Hospitalier National d'Ophtalmologie des Quinze-Vingts, Paris, France. [66]Fondation Ophtalmologique Adolphe de Rothschild, Paris, France. [67]Académie des Sciences–Institut de France, Paris, France. [68]Department of Molecular Genetics, Institute of Ophthalmology, London, UK. [69]Clinical and Experimental Sciences, Faculty of Medicine, University of Southampton, Southampton, UK. [70]University Hospital Southampton, Southampton, UK. [71]Department of Ophthalmology, Hadassah Hebrew University Medical Center, Jerusalem, Israel. [72]Center for Human Disease Modeling, Duke University, Durham, North Carolina, USA. [73]Department of Cell Biology, Duke University, Durham, North Carolina, USA. [74]Department of Pediatrics, Duke University, Durham, North Carolina, USA. [75]Centre d'Etude du Polymorphisme Humain (CEPH) Fondation Jean Dausset, Paris, France. [76]Commissariat à

l'Energie Atomique et aux Energies Alternatives (CEA), Institut de Génomique, Centre National de Génotypage, Evry, France. [77]Cole Eye Institute, Cleveland Clinic, Cleveland, Ohio, USA. [78]Department of Ophthalmology, Duke University Medical Center, Durham, North Carolina, USA. [79]Department of Medicine, Duke University Medical Center, Durham, North Carolina, USA. [80]Duke Molecular Physiology Institute, Duke University Medical Center, Durham, North Carolina, USA. [81]Bascom Palmer Eye Institute, University of Miami Miller School of Medicine, Naples, Florida, USA. [82]Department of Ophthalmology, New York University School of Medicine, New York, New York, USA. [83]Department of Ophthalmology, Royal Perth Hospital, Perth, Western Australia, Australia. [84]Department of Medical Genetics, Cambridge Institute for Medical Research, University of Cambridge, Cambridge, UK. [85]Department of Ophthalmology, Cambridge University Hospitals National Health Service (NHS) Foundation Trust, Cambridge, UK. [86]Department of Ophthalmology, University of California San Francisco Medical School, San Francisco, California, USA. [87]Department of Ophthalmology and Visual Sciences, Vanderbilt University, Nashville, Tennessee, USA. [88]Casey Eye Institute, Oregon Health and Science University, Portland, Oregon, USA. [89]Department of Human Genetics, Graduate School of Public Health, University of Pittsburgh, Pittsburgh, Pennsylvania, USA. [90]School of Clinical Sciences, University of Edinburgh, Edinburgh, UK. [91]Center for Inherited Disease Research (CIDR) Institute of Genetic Medicine, Johns Hopkins University School of Medicine, Baltimore, Maryland, USA. [92]Department of Ophthalmology, David Geffen School of Medicine, Stein Eye Institute, University of California, Los Angeles, Los Angeles, California, USA. [93]Department of Human Genetics, David Geffen School of Medicine, University of California, Los Angeles, Los Angeles, California, USA. [94]Department of Human Genetics, Radboud University Medical Centre, Nijmegen, the Netherlands. [95]Department of Pathology and Cell Biology, Columbia University, New York, New York, USA. [96]Center for Translational Medicine, Moran Eye Center, University of Utah School of Medicine, Salt Lake City, Utah, USA. [97]Division of Preventive Medicine, Brigham and Women's Hospital, Harvard Medical School, Boston, Massachusetts, USA. [98]Division of Epidemiology and Clinical Applications, Clinical Trials Branch, National Eye Institute, US National Institutes of Health, Bethesda, Maryland, USA. [99]Institute for Computational Biology, Case Western Reserve University School of Medicine, Cleveland, Ohio, USA.

Supplemental References

1. Consortium, G.T. (2015). Human genomics. The Genotype-Tissue Expression (GTEx) pilot analysis: multitissue gene regulation in humans. Science 348, 648-660.
2. Cipriani, V., Leung, H.T., Plagnol, V., Bunce, C., Khan, J.C., Shahid, H., Moore, A.T., Harding, S.P., Bishop, P.N., Hayward, C., et al. (2012). Genome-wide association study of age-related macular degeneration identifies associated variants in the TNXB-FKBPL-NOTCH4 region of chromosome 6p21.3. Hum Mol Genet 21, 4138-4150.
3. Yates, J.R.W., Sepp, T., Matharu, B.K., Khan, J.C., Thurlby, D.A., Shahid, H., Clayton, D.G., Hayward, C., Morgan, J., Wright, A.F., et al. (2007). Complement C3 variant and the risk of age-related macular degeneration. New Engl J Med 357, 553-561.
4. Fritsche, L.G., Igl, W., Bailey, J.N.C., Grassmann, F., Sengupta, S., Bragg-Gresham, J.L., Burdon, K.P., Hebbring, S.J., Wen, C., Gorski, M., et al. (2016). A large genome-wide association study of age-related macular degeneration highlights contributions of rare and common variants. Nature Genetics 48, 134-143.
5. Yang, J., Ferreira, T., Morris, A.P., Medland, S.E., Madden, P.A.F., Heath, A.C., Martin, N.G., Montgomery, G.W., Weedon, M.N., Loos, R.J., et al. (2012). Conditional and joint multiple-SNP analysis of GWAS summary statistics identifies additional variants influencing complex traits. Nature Genetics 44, 369-U170.
