# Supplementary material for: Beyond factor H: The impact of genetic-risk variants for age-related macular degeneration on circulating factor-H-like 1 and factor-H-related protein concentrations
Source: Am J Hum Genet. 2021 Jul 13;108(8):1385–400. doi: 10.1016/j.ajhg.2021.05.015 (PMC8387294; doi:10.1016/j.ajhg.2021.05.015)
Supplement: Document S2. Article plus Document S1 [file mmc6.pdf]

Beyond factor H: The impact of genetic-risk variants for age-related macular degeneration on circulating factor-H-like 1 and factor-H-related protein concentrations

Valentina Cipriani,[1,2,3,4,16,18,*] Anna Tierney,[5,16] John R. Griffiths,[5,16] Verena Zuber,[6] Panagiotis I. Sergouniotis,[7,8] John R.W. Yates,[2,3,9] Anthony T. Moore,[2,3,10] Paul N. Bishop,[7,11] Simon J. Clark,[12,13,14,17] and Richard D. Unwin[15,17,19,*]

Summary

Age-related macular degeneration (AMD) is a leading cause of vision loss; there is strong genetic susceptibility at the complement factor H (*CFH*) locus. This locus encodes a series of complement regulators: factor H (FH), a splice variant factor-H-like 1 (FHL-1), and five factor-H-related proteins (FHR-1 to FHR-5), all involved in the regulation of complement factor C3b turnover. Little is known about how AMD-associated variants at this locus might influence FHL-1 and FHR protein concentrations. We have used a bespoke targeted mass-spectrometry assay to measure the circulating concentrations of all seven complement regulators and demonstrated elevated concentrations in 352 advanced AMD-affected individuals for all FHR proteins (FHR-1, $p = 2.4 \times 10^{-10}$; FHR-2, $p = 6.0 \times 10^{-10}$; FHR-3, $p = 1.5 \times 10^{-5}$; FHR-4, $p = 1.3 \times 10^{-3}$; FHR-5, $p = 1.9 \times 10^{-4}$) and FHL-1 ($p = 4.9 \times 10^{-4}$) when these individuals were compared to 252 controls, whereas no difference was seen for FH ($p = 0.94$). Genome-wide association analyses in controls revealed genome-wide-significant signals at the *CFH* locus for all five FHR proteins, and univariate Mendelian-randomization analyses strongly supported the association of FHR-1, FHR-2, FHR-4, and FHR-5 with AMD susceptibility. These findings provide a strong biochemical explanation for how genetically driven alterations in circulating FHR proteins could be major drivers of AMD and highlight the need for research into FHR protein modulation as a viable therapeutic avenue for AMD.

Introduction

Age-related macular degeneration (AMD) is a major cause of sight loss and is estimated to affect about 290 million people by 2040.[1] A total of 34 different genetic loci (including 45 common and seven rare genetic variants) have been reported to be strongly associated with AMD risk;[2] many of these are linked to genes of the complement system, particularly those encoded on chromosomal region 1q31.3 at the "regulators of complement activation" (RCA) locus.[3] The RCA locus contains a gene cluster that regulates the alternative pathway of complement, including complement factor H (*CFH*) and five complement-factor-H-related (CFHR) genes. *CFH* encodes full-length factor H (FH) and a truncated splice variant, factor-H-like protein 1 (FHL-1), whereas the CFHR genes encode five FHR proteins, from FHR-1 to FHR-5. FH, FHL-1, and FHR-1 to FHR-5 are synthesized primarily in the liver (Figure S1), although there is evidence for local synthesis within the eye of FH and FHL-1.[4] FH and FHL-1 are cofactors for factor I, which cleaves and inactivates the central C3b protein in the complement pathway and ensures that activation is kept in check. Although the functions of FHR proteins are less well understood, there is increasing evidence that they compete with the actions of FH and FHL-1 and thereby slow the rate of C3b breakdown and stimulate complement activation.[5] AMD is a condition that primarily affects the choroid, Bruch's membrane, and retinal pigment epithelium underlying the neurosensory retina, and there is strong evidence

[1]William Harvey Research Institute, Queen Mary University of London, London, EC1M 6BQ, United Kingdom; [2]UCL Institute of Ophthalmology, University College London, London, EC1V 9EL, United Kingdom; [3]Moorfields Eye Hospital National Health Service Foundation Trust, London, EC1V 2PD, United Kingdom; [4]UCL Genetics Institute, University College London, London, WC1E 6BT, United Kingdom; [5]Division of Cardiovascular Sciences, School of Medical Sciences, Faculty of Biology, Medicine, and Health, The University of Manchester, Manchester, M13 9NY, United Kingdom; [6]Department of Epidemiology and Biostatistics, Imperial College London, London, W2 1PG, United Kingdom; [7]Division of Evolution and Genomic Sciences, School of Biological Sciences, Faculty of Biology, Medicine, and Health, University of Manchester, Manchester, M13 9PT, United Kingdom; [8]Manchester Centre for Genomic Medicine, Saint Mary's Hospital, Manchester University National Health Service Foundation Trust, Manchester, M13 9WL, United Kingdom; [9]Department of Medical Genetics, University of Cambridge, Cambridge, CB2 0QQ, United Kingdom; [10]Ophthalmology Department, University of California San Francisco, San Francisco, CA 94143-0730, USA; [11]Manchester Royal Eye Hospital, Manchester University NHS Foundation Trust, Manchester Academic Health Science Centre, Manchester, M13 9WL, United Kingdom; [12]University Eye Clinic, Department for Ophthalmology, Eberhard Karls University of Tübingen, Tübingen, Baden-Württemberg, 72076, Germany; [13]Institute for Ophthalmic Research, Eberhard Karls University of Tübingen, Tübingen, Baden-Württemberg, 72076, Germany; [14]Lydia Becker Institute of Immunology and Inflammation, Faculty of Biology, Medicine, and Health, University of Manchester, Manchester, M13 9PT, UK; [15]Stoller Biomarker Discovery Centre and Division of Cancer Sciences, School of Medical Sciences, Faculty of Biology, Medicine, and Health, The University of Manchester, Manchester, M13 9NQ, United Kingdom
[16]These authors contributed equally to this work
[17]These authors contributed equally to this work
[18]Twitter: @Val3Cipriani
[19]Twitter: @RDUnwin
*Correspondence: v.cipriani@qmul.ac.uk (V.C.), r.unwin@manchester.ac.uk (R.D.U.)
https://doi.org/10.1016/j.ajhg.2021.05.015.
© 2021 The Authors. This is an open access article under the CC BY license (http://creativecommons.org/licenses/by/4.0/).

that complement over-activation in this complex has a central role in the condition.[6,7]

CFH has undoubtedly been implicated in AMD susceptibility since the ground-breaking discovery of common risk-associated single-nucleotide polymorphisms, including coding variant rs1061170 (p.Tyr402His), within the gene;[8–11] this has been corroborated by many studies that identified rare, highly penetrant, AMD-risk-associated coding variants in *CFH*.[12–15] Downstream of *CFH*, a common deletion of *CFHR1* and *CFHR3* and a rare deletion encompassing *CFHR1* and *CFHR4* are associated with decreased risk of AMD.[16–22] However, as with other complex traits, the majority of the AMD-associated variants at the RCA locus, and indeed overall, are non-coding (i.e., six out of the eight independent association signals established on chromosomal region 1q.31.3 by the recent largest genome-wide association study (GWAS) of AMD are intronic or intergenic)[2] and are likely to manifest their effects on disease risk through genetic regulatory mechanisms.[23] Recently, it has been found that increased circulating FHR-4 is strongly associated with increased risk of AMD.[24] These observations led us to investigate whether the circulating concentrations of other FHR proteins are associated with AMD. The FHR proteins share high sequence homology (see Figure 3 of Clark and Bishop[25] for an explanatory diagram), whereas FHL-1 is a splice variant expressed from the same *CFH* gene as FH. This makes it challenging to develop antibody-based assays that can specifically measure concentrations of all seven gene products from this region in blood samples.

Here, we have developed a liquid-chromatography-selected reaction-monitoring mass-spectrometry (LC-SRM-MS)-based assay that could simultaneously measure circulating concentrations of FH, FHL-1, and FHR-1 to FHR-5. We used this assay to interrogate samples from a case-control study of AMD. We subsequently discovered that raised concentrations of FHL-1 and all five FHR proteins are strongly associated with AMD risk, and we used Mendelian randomization to assess the effects of these raised concentrations on AMD.

Material and methods

Study samples

The Cambridge AMD study is a case-control study with subjects recruited from the southeast and northwest of England between 2002 and 2006.[26,27] All affected subjects analyzed had advanced AMD, i.e., choroidal neovascularization (CNV) and/or geographic atrophy (GA). Controls were spouses, partners, or friends of index AMD individuals. Blood samples were obtained at the time of interview; EDTA and lithium-heparin plasma samples were used for DNA extraction and for FH, FHL-1, and FHR1–5 measurements, respectively. Participants were excluded if they had greater than 6 diopters of myopic refractive error or evidence of other inflammatory or retinovascular disease (such as retinal vessel occlusion, diabetic retinopathy, or chorioretinitis) that could contribute to the development of or confound the diagnosis of AMD. All participants described their ancestry as white on a recruitment questionnaire and were confirmed to be of European descent in the genetic analyses. Participants were examined by an ophthalmologist and underwent color stereoscopic fundus photography of the macular region. Images were graded at the Reading Centre, Moorfields Eye Hospital, London, via the International Classification of Age-related Maculopathy and Macular Degeneration.[28] All participants provided written informed consent for clinical examination, epidemiological data collection, and blood sampling for biochemical and genetic analyses. Ethical approval was obtained from the NRES Committee East Midlands, Derby and adhered to the tenets of the Declaration of Helsinki.

Preparation of peptide standards

High-purity heavy-labeled synthetic standards, with S-carboxymethylated (CAM) cysteine residues (denoted by a lowercase c), were obtained (Cambridge Research Biochemicals, Cambridge, UK) and diluted to 1 μg/μL with 50:50 acetonitrile:water + 0.1% v/v formic acid prior to storage at −80°C. Peptide sequences were VTY**K**cFE (FH), NGWSPTP**R**cIRVSFTL (FHL-1), ATFcD**F**PKINHGILYDEE (FHR-1), AMFcD**F**PKINHGILYDEE (FHR-2), VAcHPG**Y**GLP**K**AQTTVTcTE (FHR-3), **Y**QcQSYYE (FHR-4), and **R**GWSTPPIcSFT**K**GE (FHR-5). The residue in bold type contained an isotopically heavy amino acid, with mass increases K(+8), R(+10), F(+10), and Y(+10), respectively. A mixed, concentrated standard mixture was subsequently generated with peptides at a final concentration, in ng/μL, of 47.6 (FH), 0.95 (FHL-1), 7.14 (FHR-1), 19 (FHR-2), and 4.76 (FHR-3, FHR-4, and FHR-5). This concentrated standard was stored at −80°C in 5 μL aliquots until use.

Preparation of plasma samples for LC-SRM-MS

Frozen plasma samples were thawed to room temperature, vortexed for 5 min, and then centrifuged at 13,300 g for 30 min. A 5 μL aliquot was transferred to a 1.5 mL LoBind Eppendorf tube for processing. 90 μL of 50 mM ammonium bicarbonate (pH 7.8), 2 μL ProteaseMAX (Promega) solution (1% w/v in 50 mM ammonium bicarbonate) and 1 μL 500 mM dithiothreitol prepared in 50 mM ammonium bicarbonate was added. This was vortexed briefly, given a pulse spin, and incubated at 56°C for 25 min. After cooling to room temperature, 3 μL 500 mM iodoacetamide (prepared in 50 mM ammonium bicarbonate) was added, sample vortexed briefly, given a pulse spin, and incubated at room temperature in the dark for 15 min.

For protein digestion, 43 μL 50 mM ammonium bicarbonate (pH 7.8), 1 μL ProteaseMAX solution (1% w/v in 50 mM ammonium bicarbonate), and 5 μL 1 μg/μL endoproteinase Glu-C (Roche) were added, and the tube was vortexed and given a pulse spin before being incubated with shaking (400 rpm) for 16 h at 25°C.

We prepared standard peptides for spiking by adding 195 μL 50:50 acetonitrile:water to a 5 μL aliquot of the concentrated standard mixture. 2 μL was added to each digested sample along with 6 μL 10% v/v TFA, and samples were vortexed briefly so they were mixed, then pulse spun. This provided final standard concentrations of 500 nM (FH), 5 nM (FHL-1), 32.75 nM (FHR-1), 86.75 nM (FHR-2), 21.68 nM (FHR-3), 41.5 nM (FHR-4), and 27.5 nM (FHR-5). Samples were then dried in a centrifugal evaporator (Eppendorf) at 45°C. The dried peptides were reconstituted in 50 μL 0.1% v/v TFA and vortexed so that any residue would be dissolved before centrifugation at 13,300 g for 30 min so that any insoluble or particulate material would settle. Taking care to

leave behind any precipitated material, we transferred approximately 48 μL to a LC autosampler vial for subsequent analysis by LC-MS/MS.

LC-SRM-MS analysis of plasma digests

SRM analyses of plasma digests were performed on a 6495 triple quadrupole mass spectrometer with electrospray ion source (Agilent) (source parameters are in Table S1) coupled to an Agilent Infinity 1200 Series liquid chromatography system. Samples were injected directly (4 μL) onto a C18 column (250 mm × 2.1 mm I.D., Thermo Scientific Acclaim 120, 3 μm particle size) maintained at a temperature of 50°C. Peptides were eluted with gradient-chromatography buffer A (water + 0.1% formic acid) and buffer B (acetonitrile + 0.1% formic acid) at a flow rate of 250 μL/min at an initial composition of 5% buffer B. The following gradient was used (time, %B): 0 min, 5% B; 2 min, 5% B; 3 min, 12% B; 12 min, 15% B; 15 min, 20% B; 30 min, 25% B; 31 min, 90% B; 39 min, 90% B; 40 min, 5% B; and 49 min, 5% B. Optimized SRM settings were determined through the use of SIS solutions and are given in Table S2.

So that the source region would be protected from unwanted contaminants, a switching valve located between the column and source was diverted to the waste position at points in the chromatogram when the analyte peptides were not eluting. This allowed for six windows (two of the peptides, FHR-2 and FHL-1, eluted within the same window) of acquisition, of approximately 1 min each, to be acquired with the column on-line to the mass spectrometer.

FH, FHL-1, and the five FHR protein concentrations were determined in plasma samples from the Cambridge AMD cohort.[26,27] Samples were randomized into batches such that each batch contained a mixture of experimental and quality-control samples. Alongside 20 experimental samples, each batch contained (1) full technical duplicates on a commercial standard human serum sample, (2) a full technical replicate of one of the samples in the batch, and (3) a full technical replicate of the "duplicated" sample from the previous batch. These allowed for sample-batch quality control and assessment of batch-to-batch variability.

SRM data extraction and analysis

SRM data were processed via a dedicated project in Skyline (v19.1.0.193).[29] We visually checked retention times and heavy peptide peak areas for all samples to ensure correct peak allocations and integrations. We extracted peak-area data from Skyline into an Excel workbook, where we compared peak areas between heavy and light transitions for each peptide. We calculated the on-column loading of endogenous peptide by using the largest signal as a quantifier and the other two transitions as qualifier signals to confirm specificity and agreement in quantitation. The on-column loading of endogenous peptide was converted to a concentration per unit volume for each plasma sample on the basis of the injection of an equivalent of 0.8 μL of plasma for each sample.

Factor H measurement by ELISA

Human factor H ELISAs (Abcam) were performed on a subset of samples from the full cohort as per the manufacturer's instructions. Plasma samples were serially diluted to 1:50,000 prior to use. A mix of affected individuals and controls were selected with an even distribution across the full SRM FH result spectrum. Endpoint results were read at 450 nm with a SpectraMax M5 plate reader (Molecular Devices).

Association analysis of circulating protein concentrations with AMD

We transformed protein concentrations to ensure normality of the distribution (by using the square-root function for FH and FHR-2; FHR-3 and FHR-4; and the log function for FHL-1 and FHR-5; FHR-1 was normally distributed) when we used linear-regression models. We assessed the association of advanced AMD with concentrations of FH, FHL-1, and each of the five FHR proteins via Wald tests by using linear-regression models adjusted for sex, age, and the first two genetic principal components (as estimated within the International AMD Genomics Consortium [IAMDGC] study).[2] We also reported the association of protein concentrations with advanced AMD via odds ratio (OR) expressed as a per-one-standard-deviation (SD) change of log levels by using logistic-regression models adjusted for sex, age, and the first two genetic principal components. These statistical analyses were conducted with Stata software version 14.2 (StataCorp).

Genotype data and genome-wide association analyses

All individuals included in this study had been previously genotyped with a custom-modified Illumina HumanCoreExome array at the Centre for Inherited Disease Research (CIDR, Baltimore, Maryland, USA) and analyzed within the IAMDGC GWAS (43,566 subjects; 16,144 individuals with advanced AMD and 17,832 controls of European ancestry in the primary analysis dataset).[2] Quality-control and genotype imputation based on the 1000 Genomes Project[30] reference panel were performed by the IAMDGC as described previously.[2] We carried out GWASs of concentrations of FH, FHL-1, and all five FHR proteins (we transformed concentrations as above to ensure normality) in controls only by using linear-regression models adjusted for sex, age, and the first two genetic principal components and variants with minor-allele frequency (MAF)[3] ≥ 1% (and imputation quality, R^2,≥ 0.3, if imputed). The GWASs were carried out with the EPACTS software (version 3.3.2), and Wald tests were performed on the variant genotypes coded as 0, 1, and 2 according to the number of minor alleles for the directly typed variants or allele dosages for the imputed variants. Manhattan and Q-Q plots were generated with the *qqman* R package (version 0.1.4). Regional plots of association were generated with LocusZoom.org. Finally, linkage disequilibrium (LD) measures (R^2 and D') were calculated with LDlink (version 5.0) on the basis of the European (EUR) population genotype data originated from phase 3 (version 5) of the 1000 Genomes Project.[30]

Mendelian-randomization analysis

We used a Mendelian-randomization approach to test whether genetically proxied FHR protein concentrations are associated with risk of AMD. We used p value clumping to select independent genetic variants associated with the exposure (a protein at a time) at genome-wide significance level ($p < 5 \times 10^{-8}$) as instrumental variables (IVs) (function *ld_clump* of R package *ieugwasr*, version 0.1.5; LD cut-offs $R^2 < 0.001$ and $R^2 < 0.01$, and the default 1000 Genomes Project EUR population reference,[30] n = 489, were used for estimating LD among genetic variants). We evaluated the strength of each IV by using R^2 as the proportion of the variance of the protein explained by the genetic variant (function *get_r_from_pn* from R package *TwoSampleMR*, version 0.5.5). We also repeated the IV selection by using the GCTA-COJO[31] approach (with default settings). As a reference sample

to estimate LD among genetic variants, we used the available individual-level genotype data from the entire control set in the Cambridge AMD study,[2,26,27] n = 419, and thus ensured that the same set of variants analyzed in the GWASs of FHR protein concentrations were also used for estimating LD. It is common in Mendelian-randomization analyses of unmatched case-control studies to estimate the association of the IV with the exposure within the controls only; the justification behind this approach is that the distributions of the exposure in the general population and the control group are similar when the disease prevalence is low and that the association between the outcome and the expected exposure value conditioned on the IV is unconfounded under the IV assumptions.[32] Therefore, we carried out the IV selection by using the GWAS findings on the Cambridge AMD study[26,27] controls for whom we measured protein concentrations (n = 252).

If a single IV was available, we used the ratio-of-coefficients method, also known as the Wald method, to estimate the effect of genetically proxied protein concentrations on the disease risk.[33] The Wald ratio for a single genetic variant as IV is defined as its genetic association with the outcome (i.e., risk of AMD) over the genetic association with the exposure (i.e., protein concentration). Using a one-sample approach, we derived the genetic association with the exposure from the GWASs of the available FHR protein concentrations in the Cambridge AMD study[26,27] control individuals only (n = 252). The genetic associations with the risk of AMD were obtained from the summary GWAS estimates on the basis of a logistic-regression model with AMD status as the outcome observed in the Cambridge AMD study[26,27] (419 controls and 845 affected individuals). If multiple IVs were available for a protein, we used the inverse-variance weighted (IVW) method under a fixed-effect model[33] (function *mr_ivw* from R package *MendelianRandomization*, version 0.4.2). We assessed heterogeneity across the different single-IV estimates by using the Cochran's Q and I^2 statistics. Additionally, we calculated analogous Mendelian randomization estimates by using a two-sample approach whereby we measured the genetic association with the exposure from the FHR-concentration GWASs conducted on the Cambridge AMD study[26,27] controls only (n = 252) and the genetic associations with the AMD risk observed in the IAMDGC GWAS[2] (16,144 advanced AMD affected individuals and 17,832 control individuals of European ancestry).

Results

Development of an assay for FH, FHL-1, and FHR-1 to FHR-5

To facilitate the detection of the five FHR proteins, FH, and the splice variant FHL-1, we used mass spectrometry because this provides the necessary specificity to allow confident detection of and differentiation between similar proteins or proteoforms. Standard trypsin hydrolysis of FHL-1 yields a specific N-terminal peptide of only four amino acids, which is challenging for MS detection. Therefore, we developed an approach utilizing Endoproteinase Glu-C (V8 Protease) to produce not only distinct proteotypic peptides for all of the FHR proteins but also a unique proteotypic peptide representative of FHL-1. These peptides can thus be used for the simultaneous detection and relative quantification of all seven key regulatory proteins in a plasma sample in a single assay.

To confirm the specificity and quantitative ability of the assay, we established optimal SRM transitions on the basis of fragmentation of synthetic versions of each peptide of interest (Figure S2). Assay specificity was determined by analysis both of human plasma and of serum samples with and without synthetic peptides spiked in. Subsequently, serum samples containing stable isotope standards (SIS) peptides was analyzed. Figure S3 shows an overlay of endogenous and SIS peptides in human serum, confirming specificity.

A typical chromatogram for the assay is shown in Figure 1A. Note that, to prevent dirtying and signal decay, we diverted flow away from the source when analytes were not eluting. We determined quantitative performance by generating standard curves with the SIS peptides spiked into a Glu-C plasma digest. The assay shows excellent linearity across the dilution range (Figures 1B–1H). This allowed determination of lower limits of quantitation, defined as plasma concentrations of FH = 25 nM, FHL-1 = 0.25 nM, FHR-1 = 2 nM, FHR-2 = 1 nM, FHR-3 = 1 nM, FHR-4 = 4 nM, and FHR-5 = 3 nM.

Assay reproducibility was confirmed across all batches of samples. Duplicate commercial samples included in all batches across the experiment (74 measurements over 37 batches) demonstrated %CV for each of the following proteins: FH = 13.4%, FHL-1 = 21.0%, FHR-1 = 18.3%, FHR-2 = 15.3%, FHR-3 = 14.4%, FHR-4 = 14.6%, and FHR-5 = 9.7% (Figure S4). Triplicate AMD samples analyzed across adjacent batches were highly reproducible; 91.8% of measurements resulted in %CV < 15% (Table S3). Measured concentration for each sample is provided in Table S4 and summarized in Table 1. To further validate our assay, we also measured FH concentrations by using a commercial ELISA and compared the results to our MS data; normalized values from both approaches were generally within ~20% (Figure S5) of each other.

Circulating FHL-1 and FHR-1 to FHR-5 concentrations are higher in people with advanced AMD

Using our newly developed assay, we measured circulating concentrations of FH, FHL-1, and FHR-1 to FHR-5 in plasma samples of 352 individuals with advanced AMD and 252 phenotyped controls from the Cambridge AMD study[26,27] (Table 1, Figure 2). Although no significant difference between AMD-affected individuals and controls (p value = 0.94) was observed for FH, AMD-affected individuals showed significantly higher concentrations of FHL-1 and all five FHR proteins than did control individuals (FHL-1, $\beta = 0.08$ and $p = 4.9 \times 10^{-4}$; FHR-1, $\beta = 7.21$ and $p = 2.4 \times 10^{-10}$; FHR-2, $\beta = 0.74$ and $p = 6.0 \times 10^{-10}$; FHR-3, $\beta = 0.59$ and $p = 1.5 \times 10^{-5}$; FHR-4, $\beta = 0.56$ and $p = 1.3 \times 10^{-3}$; FHR-5, $\beta = 0.10$ and $p = 1.9 \times 10^{-4}$) (Table 1, Figures 2A–2G). The adjusted ORs of advanced AMD for a 1 SD increase of log-transformed concentrations are also presented in Table 1. Correlation

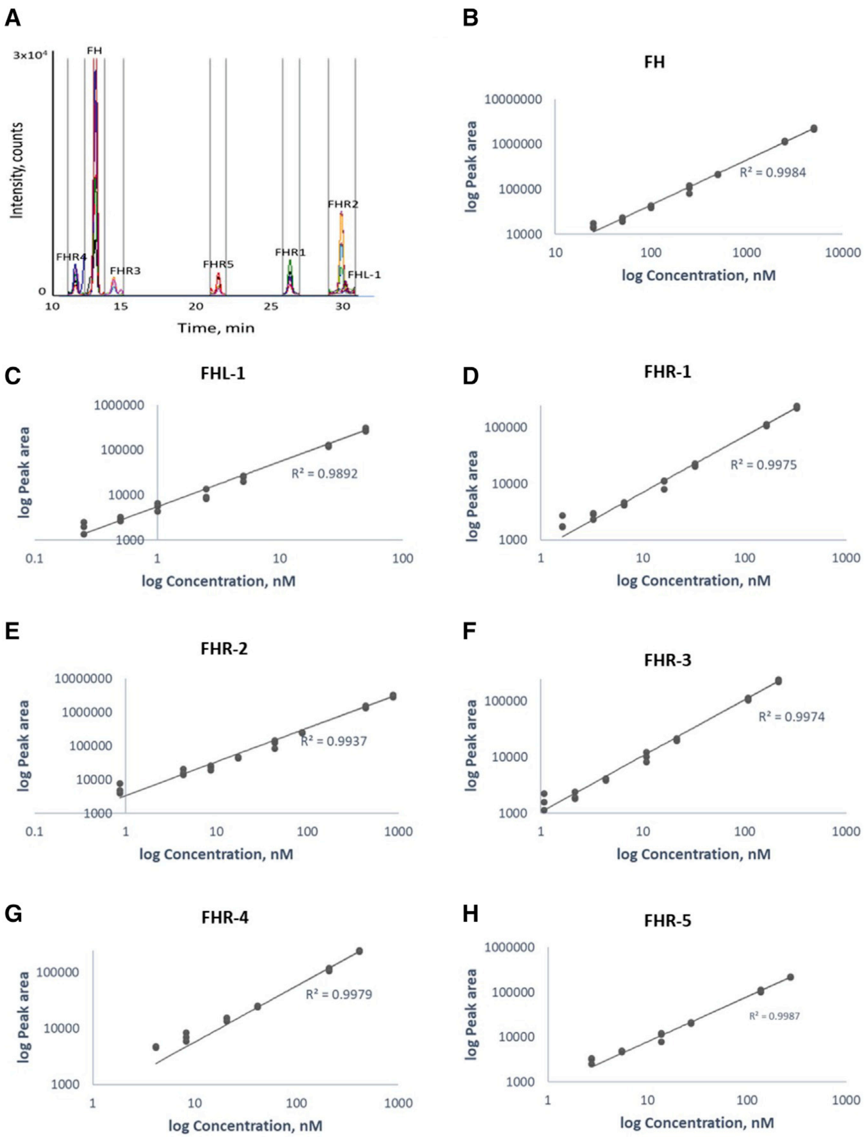

Figure 1. Development of a method for quantification of FH, FHL-1, and FHR-1 to FHR-5

(A) A total ion chromatogram from a typical LC-SRM-MS analysis of a plasma sample demonstrates low background, lack of interferences, and specific signals for each peptide.

(B–H) Assay linearity of standards in a plasma matrix showing linearity over the sample concentration range for each analyte.

analysis in the control samples demonstrated little pairwise correlation between FHRs; there was only some modest correlation between FH and FHR-5, between FHL-1 and FHR-3, between FHR-1 and FHR-3, and between FHR-2 and FHR-5 (Figure 2H). In general, it does not follow that an individual with high concentrations of one FHR protein will have higher concentrations of the others. As such, determining the concentrations of all FHR proteins is most likely important.

Genetic determinants of circulating concentrations of complement regulatory proteins overlap with the AMD-associated *CFH* locus

We performed genome-wide association analyses of the protein concentrations that were found to be elevated in individuals with advanced AMD (i.e., FHL-1 and FHR-1 to FHR-5). All GWASs of the concentrations of the five FHR proteins in 252 control individuals showed a genome-wide-significant ($p < 5 \times 10^{-8}$) peak at the *CFH* locus (Figure 3, Figure S6, Table S5). For FHR-1, FHR-2, FHR-4, and FHR-5, the *CFH* locus displayed the only observed genome-wide-significant peak for which, in the case of all the top signals, the direction of allelic effect on concentrations was concordant with the direction of effect on disease, as estimated in the IAMDGC GWAS study[2] (Table 2). FHR-3 showed a more polygenic profile, with genome-wide-significant signals at rs113721756 on chromosome 10 ($p = 1.7 \times 10^{-8}$), rs111260777 on chromosome 11 ($p = 1.5 \times 10^{-9}$), rs117468955 on chromosome 12 ($p = 3.0 \times 10^{-8}$), rs4790395 on chromosome 17 ($p = 3.6 \times 10^{-8}$), rs117115124 on chromosome 19 ($p = 2.5 \times 10^{-8}$), and rs78606172 on chromosome 20 ($p = 3.9 \times 10^{-11}$), in addition to the *CFH* locus (Table 2). The strongest signal from the GWAS of FHL-1 concentrations was observed at rs200404865 on chromosome 13 ($p = 9.6 \times 10^{-7}$), and the strongest signal at the *CFH* locus was observed at intronic *KCNT2* variant rs61820755 ($p = 5.3 \times 10^{-6}$). We also observed a block of variants in high or complete LD with the top AMD-associated intronic *CFH* variant rs10922109 [1.1] from the IAMDGC GWAS[2] as the forth signal ($p = 3.7 \times 10^{-5}$) (Figure 3, Figure S6, Table S5).

The genome-wide-significant regions of the *CFH* locus as determined from the analyses of concentrations of FHR-1 through FHR-5 overlapped among the different concentrations but showed nominally different top signals (i.e., intergenic between *CFHR1* and *CFHR4* rs149369377 for FHR-1, with $p = 2.6 \times 10^{-43}$ and $\beta = -18.2$; synonymous *CFHR2* rs4085749 for FHR-2 with $p = 6.3 \times 10^{-33}$ and $\beta = -1.5$; intronic *CFH* rs70620 for FHR-3 with $p = 1.5 \times 10^{-25}$ and $\beta = 2.0$; intergenic between *CFHR1* and *CFHR4* rs12047098 for FHR-4, with $p = 1.1 \times 10^{-17}$ and $\beta = -1.7$; and intronic *KCNT2* rs72732232 for FHR-5, with $p = 2.2 \times 10^{-10}$ and $\beta = -0.5$) (Table 2, Figure 3, Table S5).

Table 1. **Demographics of study samples and association analyses between AMD and circulating concentrations of FH, FHL-1, and FHR-1 through FHR-5**

Characteristics	Control individuals	AMD-affected individuals		
N	252	352		
Age, yr (SD)	75.2 (7.9)	73.9 (8.3)		
Male (%)	39.3	45.7		
AMD phenotype				
CNV only		218		
GA only		73		
Mixed		61		
**Protein concentrations**	**nM (95% CI)**	**nM (95% CI)**	**Association with AMD, beta, SE, P^a**	**OR (95% CI)[b]**
Mean FH concentrations	737.3 (718.2–756.5)	736.5 (721.3–751.6)	0.005, 0.23, 0.982 (0.02, 0.23, 0.936)	1.01 (0.86–1.20)
Mean FHL-1 concentrations	10.4 (10.1–10.8)	11.3 (11.0–11.7)	0.08, 0.02, 1.4×10^{-3} (0.08, 0.02, 4.9×10^{-4})	1.35 (1.14–1.60)
Mean FHR-1 concentrations	31.2 (29.4–32.9)	38.4 (37.0–39.8)	7.22, 1.12, 2.1×10^{-10} (7.21, 1.12, 2.4×10^{-10})	1.81 (1.47–2.24)
Mean FHR-2 concentrations	45.3 (43.1–47.6)	55.3 (53.2–57.4)	0.71, 0.12. 1.9×10^{-9} (0.74, 0.12, 6.0×10^{-10})	1.66 (1.38–1.98)
Mean FHR-3 concentrations	24.1 (21.7–26.5)	28.9 (27.1–30.8)	0.55, 0.13, 4.4×10^{-5} (0.59, 0.13, 1.4×10^{-5})	1.54 (1.29–1.84)
Mean FHR-4 concentrations	46.1 (42.7–49.6)	53.8 (50.5–57.1)	0.53, 0.17, 2.1×10^{-3} (0.56, 0.17, 1.3×10^{-3})	1.27 (1.08–1.50)
Mean FHR-5 concentrations	25.5 (24.5–26.5)	27.9 (27.0–28.9)	0.09, 0.03, 1.9×10^{-4} (0.10, 0.03, 1.9×10^{-4})	1.38 (1.16–1.63)

Abbreviations are as follows: AMD = age-related macular degeneration; CNV = choroidal neovascularization; GA = geographic atrophy; SE = standard error; and CI = confidence interval.
[a]Wald tests using linear-regression models; adjusted p values for sex, age, and the first two genetic principal components as estimated in Fritsche et al.[2] are displayed in parentheses
[b]Odds ratio (OR) of advanced disease expressed as the per-standard-deviation change of log levels in logistic-regression models adjusted for sex, age, and the first two genetic principal components.

These top signals are not in high LD with each other, except for rs4085749 of FHR-2 and rs12047098 of FHR-4 ($R^2 = 0.83$, $D' = 0.95$) (Table S6).

Next, we assessed whether the GWAS top signals of FHR-1 through FHR-5 protein concentrations were in LD with any of the independently AMD-associated variants at the *CFH* locus reported by the IAMDGC GWAS,[2] which also included the Cambridge samples analyzed in this study (i.e., intronic *CFH* rs10922109 [1.1]; intronic *CFH* rs570618 [1.2], proxy for p.Tyr402His; *CFH* R1210C, rs121913059 [1.3]; intergenic rs148553336 [1.4], 8 kb upstream of *CFH* and 35 kb downstream of *KCNT2*; intronic *KCNT2* rs187328863 [1.5]; intergenic rs61818925 [1.6], 14 kb downstream of *CFHR1* and 156 kb upstream of *CFHR4*; intronic *CFH* rs35292876 [1.7]; intronic *CFHR5* rs191281603 [1.8]; Table 3). The rare *CFH* variant rs121913059 (p.Arg1210Cys), [1.3][13] was present heterozygously in a single affected individual from the Cambridge study and was excluded from this analysis. The top signal for FHR-1 was in modest LD with the top AMD-associated variant 1.1 ($R^2 = 0.30$) and low LD with the proxy for p.Tyr402His 1.2 ($R^2 = 0.12$); the top signal for FHR-2 was in modest LD with 1.1 ($R^2 = 0.35$) and 1.6 ($R^2 = 0.36$) and in low LD with 1.2 ($R^2 = 0.16$); similar results were obtained for the top signal of FHR-4 ($R^2 =$ 0.38, 0.42, and 0.16 with 1.1, 1.6, and 1.2, respectively); low LD was seen with 1.1, 1.2, and 1.6 ($R^2 = 0.16$, 0.12, and 0.11, respectively) for the top signal of FHR-3, and the top signal of FHR-5 was in low to modest LD with 1.4 ($R^2 = 0.26$) (Table S6).

Furthermore, genome-wide-significant associations were observed at the top IAMDGC variant rs10922109 (1.1) with $p = 8.6 \times 10^{-21}$, 2.9×10^{-10}, 2.2×10^{-16}, and 1.7×10^{-9} for FHR-1, FHR-2, FHR-3, and FHR-4, respectively; at the proxy for p.Tyr402His 1.2 with $p = 2.0 \times 10^{-11}$ and 1.8×10^{-12} for FHR-1 and FHR-2, respectively; and at the variant 1.6 with $p = 1.8 \times 10^{-11}$ and 2.4×10^{-9} for FHR-2 and FHR-4, respectively. For all these genetic associations, the direction of allelic effect on respective protein concentrations was concordant with that on disease as estimated in the IAMDGC GWAS[2] (Table 3, Figure 4). Altogether, these GWAS findings support the hypothesis that the *CFH* locus AMD-risk variants increase disease risk through increase of FHR concentrations.

We also carried out a GWAS of the FH concentrations in controls and observed no genome-wide-significant associations; the strongest signal was at the *CFH* locus for intronic *CFHR2* rs114036234 ($p = 4.7 \times 10^{-6}$ and $\beta = -3.0$), and the direction of allelic effect on concentrations was concordant with that on disease as estimated in the IAMDGC GWAS study[2] ($p = 2.5 \times 10^{-9}$ and $\beta = -0.3$) (Figure S6, Table S5).

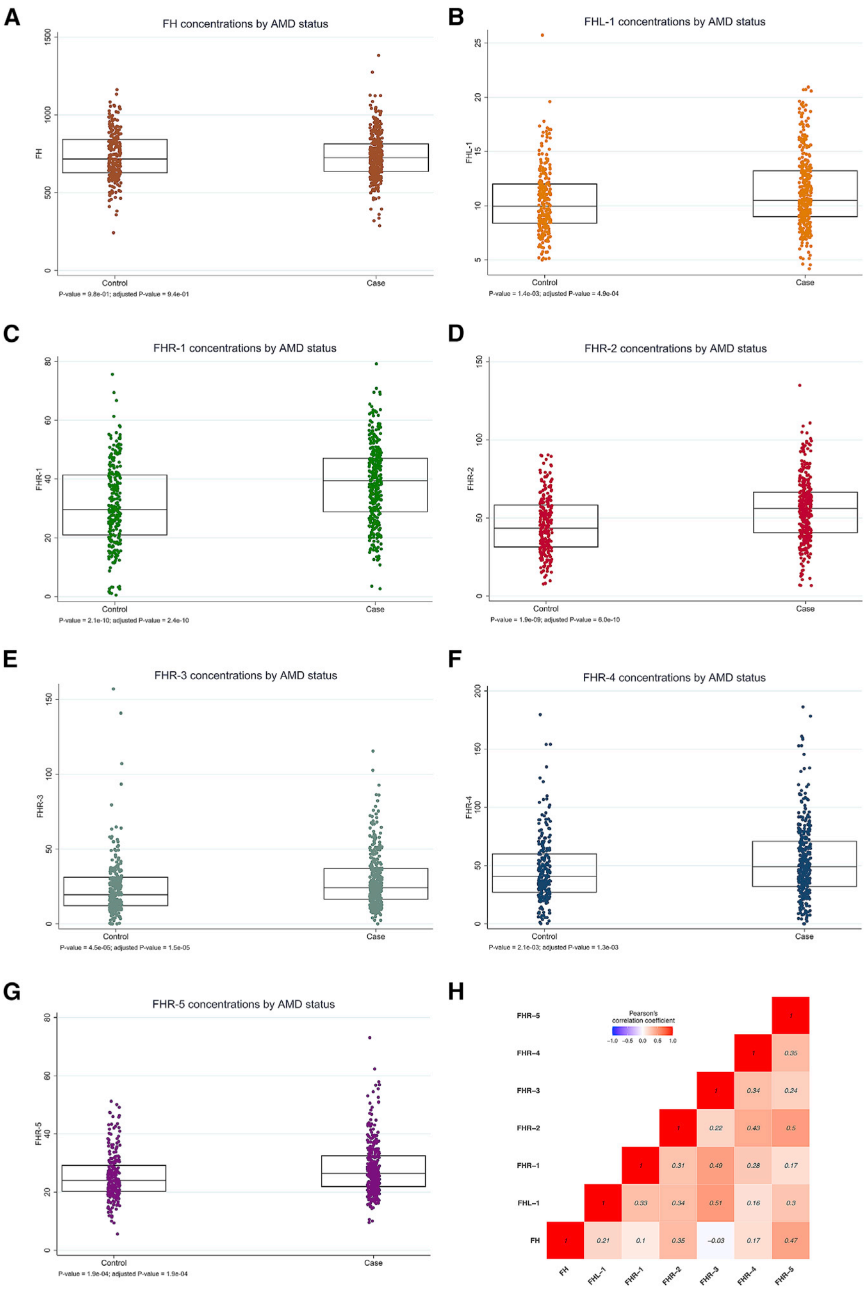

Figure 2. Circulating concentrations of FHL-1 and FHR-1 to FHR-5 are elevated in AMD-affected individuals

Boxplots of FH (A), FHL-1 (B), and FHR-1 to FHR-5 (C–G) protein concentrations measured in plasma samples of 352 individuals with advanced AMD and 252 phenotyped control individuals from the Cambridge AMD study.[26,27] Protein concentrations are expressed as nM. Individuals with AMD show statistically significant elevated concentrations of FHL-1 and FHR-1 through FHR-5 in comparison to controls, whereas no significant difference between individuals with AMD and controls was observed for FH concentrations. Unadjusted p values were obtained from Wald tests via linear-regression models and are presented together with p values adjusted for sex, age, and the first two genetic principal components (as estimated within the IAMDGC study[2]). (H) Pairwise Pearson's correlation coefficients (r) between the seven protein concentrations for the 252 control samples. Some modest correlation is observed between FH and FHR-5, FHL-1 and FHR-3, FHR-1 and FHR-3, and FHR-2 and FHR-5.

Mendelian-randomization estimates of the effects of circulating concentrations of complement regulatory proteins on susceptibility to AMD

We used the Mendelian-randomization approach to test whether genetically proxied FHR concentrations are associated with risk of AMD. Table 2 reports details of the IVs (genetic variants) selected via the p-value-clumping method with an LD cut-off $R^2 < 0.001$ and the 1000 Genomes Project EUR population reference. When we used an LD cut-off of $R^2 < 0.01$, the same IVs were selected. Figure 5 shows the Mendelian-randomization estimates of the FHR concentrations obtained via the one-sample and two-sample Wald ratio (if a single IV was selected: FHR-1, FHR-2, FHR-4, or FHR-5) or IVW method (if multiple IVs were selected: FHR-3) together with the traditional epidemiologic estimates of the association of the protein concentrations with AMD as obtained from logistic-regression models and ORs (Table 1). The variance of the FHR concentrations explained by each single genetic instrument varied from 0.11 to 0.53 (Table 2).

The Mendelian-randomization estimates were statistically significant and of concordant direction with the observational OR estimates for FHR-1, FHR-2, FHR-4, and FHR-5, providing evidence in support of a causal effect (Figure 5). We observed overlapping CIs for the one-sample and the two-sample Mendelian-randomization estimates; the latter showed higher accuracy with much narrower CIs, likely reflecting the larger dataset used for estimating the genetic associations with the risk of AMD (i.e., IAMDGC GWAS[2]). For FHR-3, the IV at the *CFH* locus was the only one that showed a significant association with AMD risk, but the direction of allelic effect on protein concentrations was discordant from that on disease. The corresponding Mendelian-randomization estimate did not support an association of FHR-3 with the disease (one-sample: 0.99, 95% CI = 0.93–1.06, $I^2 = 0\%$; two-sample: 0.99, 95% CI = 0.98–1.01, $I^2 = 57\%$). The GWAS of FHL-1 did not show any genome-wide-significant signals that could be used as genetic instruments in the

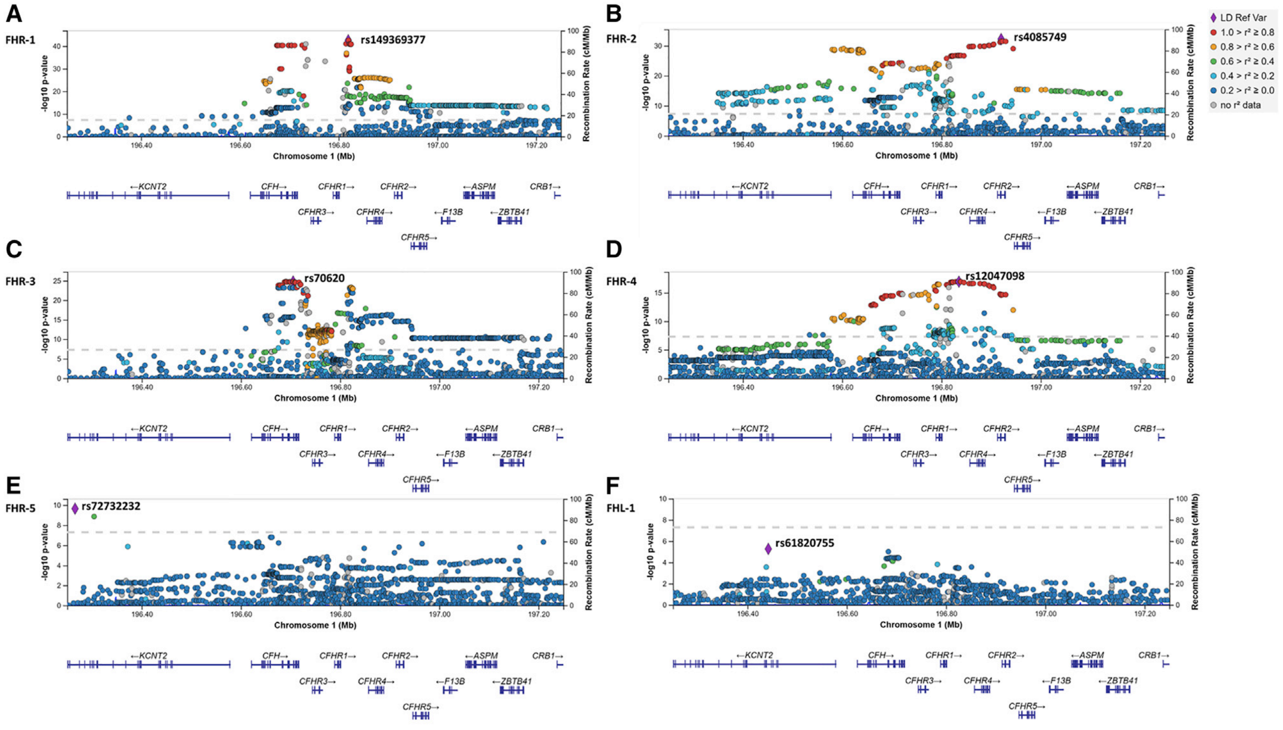

Figure 3. GWASs of circulating concentrations of FHR-1 through FHR-5 reveal a strong genome-wide-significant signal spanning the *CFH* locus

Regional plots show the genome-wide-significant ($p < 5 \times 10^{-8}$) association signals from the GWASs of FHR-1 through FHR-5 concentrations (A–E) at the *CFH* locus on chromosomal region 1q31.3. (F) The equivalent *CFH* region for the GWAS of FHL-1 concentrations (no genome-wide-significant association regions were observed). The most associated variant is denoted by a purple diamond and is labeled by its rs number. The other surrounding variants are shown by circles colored to reflect the extent of linkage disequilibrium with the most associated variant (on the basis of the European [EUR] population genotype data originated from the 1000 Genomes Project, November 2014). A diagram of the genes within the relevant regions is depicted below each plot. Physical positions are based on NCBI RefSeq hg19 human genome reference assembly.

Mendelian-randomization analysis. It is worth noticing that the strongest FHL-1 GWAS signal at the *CFH* locus was observed at rs61820755 ($p = 5.3 \times 10^{-6}$, $\beta = 0.22$) and that this variant did not show association with AMD in the Cambridge AMD study ($p = 0.74$; $\beta = 0.05$) or in the IAMDGC study[2] ($p = 0.50$; $\beta = -0.02$).

Finally, we repeated the IV selection by using the GCTA-COJO[31] approach with the available individual-level genotype data from the entire control set genotyped with the same array in the Cambridge AMD study[2,26,27] as a reference for LD estimates (n = 419). The same sets of IVs as presented in Table 2 were identified for all FHR proteins. Additional secondary signals that could be used as IVs were identified for FHR-2 (rs79351096), FHR-3 (rs16840522), and FHR-4 (rs34538561) at the *CFH* locus (Table S7). These additional signals are not in high LD with each other or with the primary FHR signals (Table 2), except for rs16840522 of FHR-3 with the top signal of FHR-1 rs149369377 ($R^2 = 0.96$, $D' = 0.99$). The corresponding Mendelian-randomization estimates for FHR-2 (one-sample: 1.58, 95% CI = 1.39–1.80; two-sample: 1.46, 95% CI = 1.42–1.50) overlapped with the ones based on a single IV selected via p-value clumping (Figure 5), although heterogeneity was observed ($I^2 = 66\%$ and $I^2 = 94\%$ for the one-sample and the two-sample estimates, respectively). The Mendelian-randomization estimate for FHR-3 became significant (one-sample: 1.07, 95% CI = 1.01–1.13; two-sample: 1.08, 95% CI = 1.07–1.09) and showed high heterogeneity ($I^2 = 80\%$ and $I^2 = 99\%$ for the one-sample and the two-sample estimates, respectively). The Mendelian-randomization estimate for FHR-4 was refined to 1.19, 95% CI = 1.07–1.32 (one-sample) and 1.10, 95% CI = 1.08–1.12 (two-sample) and also showed high heterogeneity ($I^2 = 98\%$ and $I^2 = 99\%$ for the one-sample and the two-sample estimates, respectively). The Mendelian-randomization estimates calculated from secondary signals should be interpreted with caution given that our GWASs of FHR protein concentrations (n = 252) might have relatively moderate or small power for dissecting independent secondary signals with accuracy.

Discussion

This study adds compelling evidence that genetically driven elevated circulating concentrations of FHR proteins are strongly associated with AMD. Earlier genetic studies identified a common deletion of *CFHR1* and *CFHR3* and a rare deletion encompassing *CFHR1* and *CFHR4* as being protective against AMD.[16–22] The mechanism behind these

Table 2. Instrumental variables (IVs) of FHR protein concentrations and their corresponding genetic-association estimates for FHR protein concentrations and AMD

				Association with protein concentrations in 252 Cambridge controls			Association with AMD in the Cambridge AMD GWAS[26,27] (845 AMD-affected individuals and 419 control individuals)			Association with AMD in the IAMDGC GWAS[2] (16,144 AMD-affected individuals and 17,832 controls)			
**Protein**	**Instrumental variable (IV) : dbSNP ID; (Chr: position)[a]; non-effect allele/effect allele**	***cis/trans* pQTL**	**IV strength (R^2)[b]**	**Beta**	**SE**	**p value**	**Beta**	**SE**	**p value**	**Beta**	**SE**	**p value**	**Minor-allele frequency**
FHR-1	rs149369377; (1: 196819479_A/G); *CFHR2* intronic	*cis*	0.53	−18.15	1.07	2.6×10^{-43}	−0.76	0.13	1.9×10^{-9}	−0.87	0.02	7.6×10^{-295}	0.157
FHR-2	rs4085749; (1: 196920148_C/T); *CFHR2* synonymous	*cis*	0.44	−1.55	0.11	6.3×10^{-33}	−0.78	0.11	2.0×10^{-12}	−0.62	0.02	2.2×10^{-184}	0.192
FHR-3	rs70620; (1:196704997_G/A); *CFH* intronic	*cis*	0.35	2.02	0.17	1.5×10^{-25}	−0.06	0.12	0.581	−0.07	0.02	2.7×10^{-3}	0.162
	rs78606172; (20: 62087676_G/A); *KCNQ2* intronic	*trans*	0.16	7.61	1.10	3.9×10^{-11}	−0.20	0.56	0.721	0.14	0.11	0.210	0.010
	rs111260777; (11: 127117796_T/C); intergenic	*trans*	0.14	4.36	0.69	1.5×10^{-9}	−0.04	0.47	0.924	−0.01	0.07	0.865	0.018
	rs113721756; (10: 116647277_C/T); *FAM160B1* intronic	*trans*	0.12	4.92	0.84	1.7×10^{-8}	−0.76	0.54	0.161	0.12	0.09	0.187	0.012
	rs11711512; (19: 56030803_G/A); intergenic	*trans*	0.12	4.57	0.79	2.5×10^{-8}	0.75	0.45	0.096	0.09	0.10	0.351	0.016
	rs117468955; (12: 92269900_A/G); intergenic	*trans*	0.12	4.72	0.82	3.0×10^{-8}	0.06	0.53	0.913	−0.03	0.10	0.789	0.015
	rs4790395; (17: 2852632_C/T); *RAP1GAP2*	*trans*	0.11	−6.50	1.14	3.6×10^{-8}	−0.04	0.78	0.955	0.16	0.13	0.206	0.031
FHR-4	rs12047098; (1: 196835106_T/C); *CFHR2* intronic	*cis*	0.25	−1.75	0.19	1.1×10^{-17}	−0.86	0.12	8.9×10^{-14}	−0.67	0.02	5.5×10^{-198}	0.172
FHR-5	rs72732232; (1: 196265545_T/A); *KCNT2* intronic	*cis*	0.15	−0.52	0.08	2.2×10^{-10}	−1.56	0.32	1.4×10^{-6}	−0.86	0.07	3.2×10^{-41}	0.026

Number of genetic variants associated with concentrations of a protein at genome-wide significance level ($p < 5 \times 10^{-8}$): FHR-1: 529 on chromosome 1; FHR-2: 553 on chromosome 1; FHR-3: 611 on chromosome 1, 1 on chromosome 10, 2 on chromosome 11, 1 on chromosome 12, 1 on chromosome 17, 3 on chromosome 19, 1 on chromosome 20; FHR-4: 253 on chromosome 1; FHR-5: 2 on chromosome 1. IVs were selected using the P value clumping method. P value clumping was performed with function *ld_clump* of R package *ieugwasr*, version 0.1.5; LD cut-offs $R^2 < 0.001$ and the 1000 Genomes Project EUR population reference.[30]
AMD = Age-Related macular degeneration; GWAS = Genome-wide association study; IAMDGC = International Age-Related Macular Degeneration Genomics Consortium; pQTL = protein quantitative trait locus.
[a]Chromosomal position is given according to the NCBI RefSeq hg19 human genome reference assembly;
[b]The strength of each IV was evaluated using R^2 as the proportion of the variance of the protein explained by the genetic variant(s) (function *get_r_from_pn* from R package *TwoSampleMR*, version 0.5.5).

Table 3. Single-variant association analyses for the eight established AMD independently associated variants at the *CFH* locus with concentrations of FH, FHL-1, and FHR-1 to FHR-5 in control individuals

				Association with protein concentrations in 252 Cambridge AMD study[26,27] control individuals[a]						
				FH	FHL-1	FHR-1	FHR-2	FHR-3	FHR-4	FHR-5
IAMDGC[2] association signal number (direction[b])	dbSNP ID (Chr: position)[c]; major/minor allele (imputation R^2)[d]	IAMDGC OR (MAF in controls)	MAF, Cambridge controls	Beta (SE); p	Beta (SE); p	Beta (SE); p	Beta (SE); p	Beta (SE); p	Beta (SE); p	Beta (SE); p
1.1 (−)	rs10922109 (1: 196704632); C/A (1.00)	0.38 (0.426)	0.422	0.49 (0.25), 0.056	−0.10 (0.02); 3.7×10^{-5}	−10.67 (1.04); 7.8×10^{-21}	−0.75 (0.11); 2.9×10^{-11}	−1.20 (0.14); 1.7×10^{-16}	−1.0 (0.17); 1.5×10^{-9}	−0.04 (0.03); 0.184
1.2 (+)	rs570618 (1: 196657064); G/T (1.00)	2.38 (0.364)	0.357	0.27 (0.26); 0.296	0.05 (0.03); 0.046	8.19 (1.17); 2.0×10^{-11}	0.85 (0.11); 1.8×10^{-12}	0.16 (0.16); 0.304	0.62 (0.18); 6.8×10^{-4}	0.10 (0.03); 7.8×10^{-4}
1.3 (+)	rs121913059 (1: 196716375); C/T (genotyped)	20.28 (0.00014)	0	*no control carrier observed; not analyzed*						
1.4 (−)	rs148553336 (1: 196613173); T/C (genotyped)	0.29 (0.009)	0.017	−2.29 (1.02); 0.025	−0.09 (0.10); 0.353	0.06 (5.00); 0.990	−1.99 (0.48); 4.6×10^{-5}	0.33 (0.62); 0.603	0.74 (0.72); 0.302	−0.55 (0.11); 6.3×10^{-7}
1.5 (+)	rs187328863 (1: 196380158); C/T (0.83)	2.27 (0.028)	0.013	1.12 (1.34); 0.404	0.06 (0.13); 0.660	0.75 (6.52); 0.908	1.13 (0.64); 0.080	−0.51 (0.81); 0.536	0.61 (0.94); 0.515	0.01 (0.15); 0.956
1.6 (−)	rs61818925 (1: 196815450); G/T (0.87)	0.60 (0.385)	0.405	−0.42 (0.27); 0.124	0.001 (0.03); 0.962	0.99 (1.33); 0.459	−0.93 (0.12); 1.3×10^{-13}	0.85 (0.16); 1.7×10^{-7}	−1.12 (0.18); 1.5×10^{-9}	−0.07 (0.03); 0.014
1.7 (+)	rs35292876 (1: 196706642); C/T (genotyped)	2.42 (0.009)	0.004	*MAF ≤ 1%; not analyzed*						
1.8 (+)	rs191281603 (1: 196958651); C/G (0.42)	1.07 (0.006)	0.008	*MAF ≤ 1%; not analyzed*						

Abbreviations are as follows: AMD = age-related macular degeneration; IAMDGC = International Age-Related Macular Degeneration Genomics Consortium; and MAF = minor-allele frequency.
[a]Wald tests using linear-regression models adjusted for sex, age, and the first two genetic principal components as estimated within the IAMDGC study.[2]
[b]Direction of association with AMD for the minor allele, as estimated in the IAMDGC study.[2]
[c]Chromosomal position is given according to the NCBI RefSeq hg19 human genome reference assembly.
[d]Imputation quality metric R^2 as estimated in the IAMDGC study.[2]

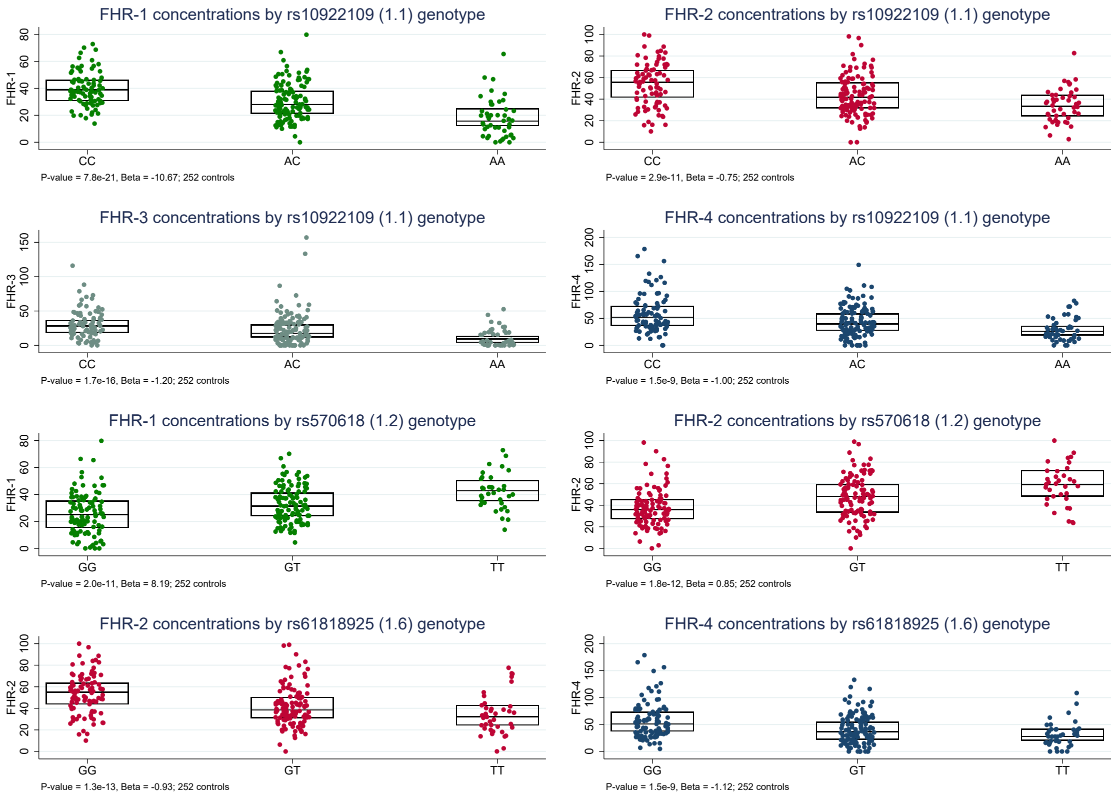

Figure 4. Established AMD-risk variants at the *CFH* locus are associated with circulating FHR-1, FHR-2, FHR-3, and FHR-4 concentrations in control individuals

Boxplots of FHR concentrations by variant genotype for those *CFH* variants that were established as conferring AMD risk in the IAMDGC study[2] and that showed genome-wide-significant ($p < 5 \times 10^{-8}$) associations in 252 controls from the Cambridge AMD study[26,27] cohort (Table 3). p values and beta values from Wald tests using linear-regression models adjusted for sex, age, and the first two genetic principal components (as estimated within the IAMDGC study) are indicated in the note at the bottom of each plot.

protective associations has been assumed to revolve around the FHR proteins' being complement activators.[25] More direct evidence that FHR proteins drive complement activation in AMD came from the discovery that increased circulating concentrations of FHR-4 in AMD-affected individuals are driven by known *CFH*-locus AMD-risk variants.[24] The protein itself was shown to accumulate in the intercapillary septa of human eyes, the primary site of complement over-activation associated with AMD.[34] In the study reported here, we show that in fact the concentrations of all five circulating FHR proteins are elevated in advanced cases of AMD (Table 1, Figure 2). Furthermore, by developing a unique mass-spectrometry-based measuring technique, we were able to measure for the first time both *CFH* splice variants: FH and FHL-1. Our data confirmed previous findings that circulating FH concentrations do not change with disease.[24] Despite the lack of differing FH concentrations, we observed that circulating concentrations of FHL-1 were statistically elevated in AMD-affected individuals ($p = 4.9 \times 10^{-4}$) (Table 1, Figure 2). However, we did not find that any genome-wide-significant signals from the GWAS of FHL-1 in 252 controls (Figure S6, Table S5) could serve as genetic instruments in our Mendelian-randomization analysis. As such, the elevation of FHL-1 concentrations in advanced cases of AMD remains observational.

The molecular mechanisms underpinning the genetic AMD risk carried on chromosomal region 1q31.3, and indeed how it contributes to complement over-activation, have been widely debated. Some genetic risk variants have obvious effects, such as the FH and FHL-1 polymorphism p.Tyr402His, which reduces their binding to the extracellular matrix in the choriocapillaris and thus leads to less support for the degradation of C3b.[35,36] However, the role of non-coding AMD-risk variants in the *CFH* locus has been much harder to dissect, and an assumption that they somehow alter the expression or function of FH (or FHL-1) itself has prevailed. Indeed, given the previous inability to simultaneously measure concentrations of the five FHR proteins, FH, and FHL-1, this has until now remained unchallenged.

Mass spectrometry provides high levels of specificity for protein quantitation because proteins (and proteotypic peptides) are identified accurately by their mass and fragmentation patterns. This makes mass spectrometry ideally suited to the detection of protein splice variants and isoforms, and in this case to the analysis of FHL-1, which differs from FH only via a unique 4 amino acid N-terminal

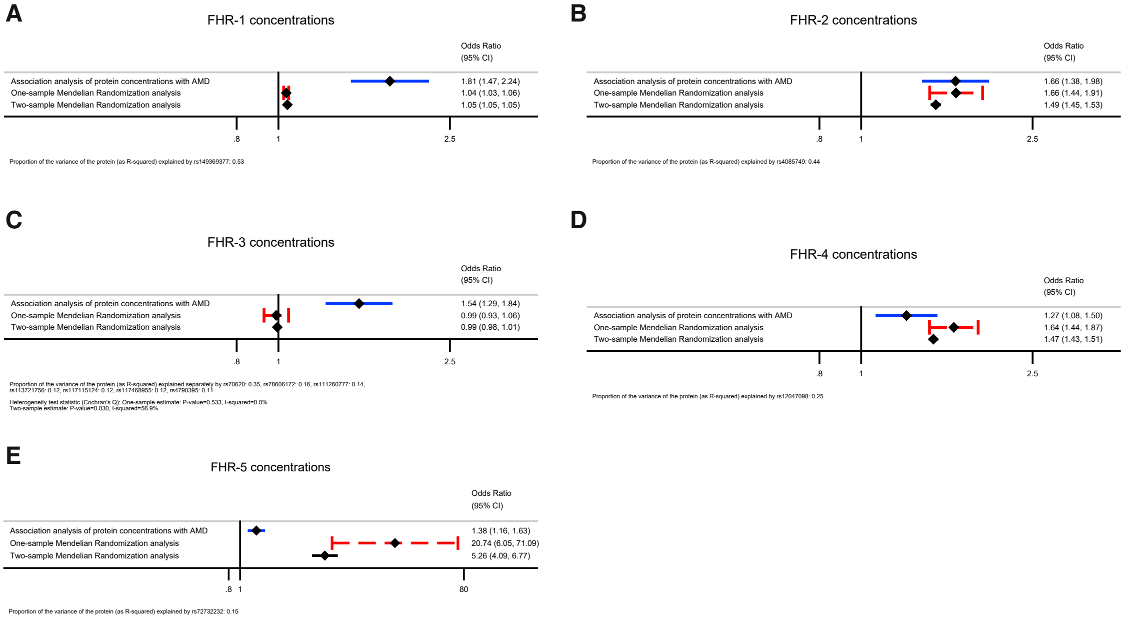

Figure 5. Mendelian-randomization analysis shows a highly significant elevation of circulating FHR-1, FHR-2, FHR-4, and FHR-5 concentrations in advanced AMD
One-sample and two-sample Mendelian-randomization estimates of the association of FHR-1 (A), FHR-2 (B), FHR-3 (C), FHR-4 (D), and FHR-5 (E) are presented together with the corresponding traditional epidemiologic odds ratio (OR) estimates obtained from logistic-regression models (352 individuals with advanced AMD and 252 control individuals from the Cambridge AMD study). The Mendelian-randomization estimates were obtained from the Wald ratio (if a single instrument was available: FHR-1, FHR-2, FHR-4, and FHR-5) or the inverse-variance weighted (IVW) method under a fixed-effect model (if multiple instruments were available: FHR-3). Raw data used for calculation of the Mendelian-randomization estimates are provided in Table 2. The proportion of the variance of each protein (as R^2) explained by its genetic instrument(s) is indicated in the note at the bottom of each plot.

sequence. Here we can access an FHL-1 proteotypic peptide by using an alternative protease, GluC, to allow detection of FHL-1 and accurate measurement of its concentrations in the circulation. The multiplex nature of mass spectrometry also allows additional proteins to be added to the same assay, such that we can identify all seven key proteins encoded at the RCA locus in a single experiment. The addition of stable-isotope-labeled peptides subsequently allows quantitation, which we show here to be stable and precise across many hundreds of AMD samples, providing a powerful tool for the study of FH, FHL-1, and FHR-1 through FHR-5 in complement regulation. This approach has been tried previously[37] with a standard trypsin enzyme for proteolysis; this allows for measurement of FHR proteins, but not FHL-1. The concentrations of FHR proteins reported here are similar (within 2x) to those reported by Zhang et al.[37] Mean protein concentrations determined in our assay are slightly lower, with the exception of FHR-3, where we report mean concentrations in control samples of 24.1 nM, or 0.9 μg/mL, versus 0.02 μg/mL reported by Zhang et al.,[37] and FHR-5, where we report mean control concentrations of 25.5 nM, or 1.6 μg/mL, versus 5.5 μg/mL, possibly because Zhang et al.[37] used different fragment ion transitions for the endogenous peptides and their equivalent heavy-labeled standards.

AMD represents a paradigm in the field of complex genetics since the seminal discovery in 2005 of the *CFH* as a major susceptibility gene.[8–11] With this study we continued to dissect the role of the *CFH* locus in AMD, beyond FH. The FH, FHL-1, and FHR-1 through FHR-5 proteins are mainly synthesized in the liver (Figure S1), so measurement of circulating concentrations in plasma allows exploration of the effects of non-coding *CFH* variants that are strongly associated with AMD risk.[2] Using 252 non-AMD controls to get insights into the genetic determinants of the circulating protein concentrations measured in this study, we discovered that genome-wide-significantly associated variants in our analyses of the FHR protein concentrations overlap with the AMD-associated *CFH* region; Figure 3). Established genetic associations with AMD risk at the non-coding variants 1.1, proxy for p.Tyr402His 1.2, and 1.6 translated into genome-wide-significant associations with concentrations of FHR-1, FHR-2, FHR-3, and FHR-4 from the GWASs in our control group (Table 3, Figure 4).

The identification of the *CFH* locus as a *cis* protein quantitative-trait locus (*cis*-pQTL) associated with concentrations of the five FHR proteins prompted us to use the available genetic data in a Mendelian-randomization fashion to triangulate this evidence. Mendelian randomization is increasingly being used because it can overcome major limitations such as unmeasured confounding and/or reverse causality in studies of the relationship between a modifiable exposure and a disease

outcome or trait, and it is becoming a standard for assessing new drug targets.[38,39] For FHR-3 the Mendelian-randomization approach suggests that the association with AMD as estimated by a traditional observational OR (Table 1) might have arisen from residual confounding and/or reverse causality (Figure 5). For FHR-1, FHR-2, FHR-4, and FHR-5, on the other hand, the statistical support provided by the univariate Mendelian-randomization analyses for a potential casual role in susceptibility to AMD is striking, and Mendelian-randomization estimates corroborate the preliminary evidence shown by the observational OR estimates (Table 1, Figure 5). This finding reframes our understanding of the etiology of AMD and the role of the non-coding risk variants on chromosome 1q31.3, demonstrating that the FHR proteins play a prominent role that requires significant further research.

Among the methodological approaches that use genetic data to assess relationships between risk factors and outcomes, Mendelian randomization is the only one that directly assesses the causal effect of a risk factor on an outcome.[40] Nevertheless, there are still questions that remain unanswered by the present study and could be addressed through the use of recently developed analytical tools to perform, for example, multivariate analyses of the FHR protein concentrations as well as conditional and genetic colocalization analyses.[41–45] At present, given the relatively moderate statistical power of our study, we did not fully disentangle the relative role of the concentration of each FHR protein on AMD risk and/or narrowing down the specific *CFH* genes and genetic variants that are likely to be involved in the causal cascade with AMD. There was limited pairwise correlation between the FHR protein concentrations in controls (Figure 2), and the GWAS signals of the FHR protein concentrations are not in high LD with each other, except for the top signals of FHR-2 and FHR-4 ($R^2 = 0.83$, $D' = 0.95$, Table S6) and for both the top signal of FHR-1 and the secondary signal of FHR-3 ($R^2 = 0.96$, $D' = 0.99$). As such, whether each of the FHR proteins that showed a strong association with advanced AMD coupled with a significant Mendelian-randomization estimate is independently causal to AMD needs further investigation. Moreover, the top GWAS signals of the FHR protein concentrations showed low to modest LD with AMD-risk variants 1.1, 1.2, 1.4, and 1.6 (Table S6). Although this might suggest a modest genetic colocalization between the corresponding FHR protein concentrations and AMD, it is worth noting that also the top GWAS signal of FHR-4 in our previously published study on the Cambridge samples[24] showed only modest LD with the top AMD-associated variant rs10922109 [1.1] (rs61818890, $R^2 = 0.49$). However, when the Cambridge results were meta-analyzed with data from a second cohort (EUGENDA), the top GWAS signal of FHR-4 was found to be in high LD with 1.1 (rs10737680, $R^2 = 0.98$). We expect that once FHR protein concentrations are available for larger datasets, new high-powered analyses will help clarify further questions that currently remain unanswered by our study.

Finally, the data presented in this study additionally highlight the targeting (and lowering) of FHR proteins in the circulation as a viable therapeutic avenue for AMD. Indeed, delivery of a systemic therapeutic provides evidence that a paradigm in ocular therapeutic strategies could allow affected individuals to avoid surgical procedures, especially in the early stages of disease before the loss of visual acuity, where therapeutic intervention might yield the most benefit. AMD individual stratification would be important because only a proportion of AMD-affected individuals are likely to suffer from FHR-mediated disease. However, as demonstrated here, an AMD-affected individual's genetic-risk profile, coupled with measurements of their circulating FHR protein concentrations, could possibly be used in the future to identify and stratify those affected people most likely to benefit from such treatments and to monitor their response to FHR-lowering agents.

Data and code availability

The mass-spectrometry data are available via the ProteomeXchange data repository under accession number PXD023466. The Genotype-Tissue Expression (GTEx) Project[46] dataset used for the gene expression analyses was obtained from the GTEx portal, dataset dbGaP accession number dbGaP: phs000424.v8.p2 (GTEx Analysis Release V8); the GTEx Project was supported by the Common Fund of the Office of the Director of the National Institutes of Health and by NCI, NHGRI, NHLBI, NIDA, NIMH and NINDS.

Supplemental information

Supplemental information can be found online at https://doi.org/10.1016/j.ajhg.2021.05.015.

Acknowledgments

We are grateful to all the subjects who kindly participated in this research. For the Cambridge AMD Study (UK Medical Research Council grant G0000067 to J.R.W.Y. and A.T.M.), we gratefully acknowledge help with patient recruitment from members of the Genetic Factors in AMD Study Group (P. Black, Z. Butt, V. Chong, C. Edelsten, A. Fitt, D.W. Flanagan, A. Glenn, S.P. Harding, C. Jakeman, C. Jones, R.J. Lamb, V. Moffatt, C.M. Moorman, R.J. Pushpanathan, E. Redmond, T. Rimmer, and D.A. Thurlby); we thank Jane Khan and Humma Shahid for carrying out the clinical evaluation and sampling of subjects and Tunde Peto and colleagues at the Reading Centre, Moorfields Eye Hospital, London, for grading the fundus photographs. We are grateful to Dr. Serena Sanna for the helpful discussion about causality and Mendelian-randomization analysis. We also thank the IAMDGC (for a full list of consortium members, please see the supplemental information) for providing the genotype data for the Cambridge AMD study samples and the consortium summary association statistics for the two-sample Mendelian-randomization analysis. The Cambridge samples were genotyped as part of the IAMDGC exomechip project supported by Centre for Inherited Disease Research in Baltimore, MD (contract number HHSN268201200008I) and funded

by EY022310 (to J.L. Haines, Case Western Reserve University, Cleveland) and 1x01HG006934-01 (to G.R. Abecasis, University of Michigan, Department of Biostatistics). This work was funded by the Medical Research Council (MR/P025838/1) and facilitated by the Manchester National Institute for Health Research Biomedical Research Centre and the Greater Manchester Comprehensive Local Research Network. The Stoller Biomarker Discovery Centre was established with an award from the Medical Research Council (MR/M008959/1). S.J.C. is funded by the Helmut Ecker Foundation, Germany. The funding bodies had no role in the design of the study, in the collection, analysis, or interpretation of data, or in writing the manuscript.

Declaration of interests

P.N.B., S.J.C., and R.D.U. are inventors named in patent applications that describe the use of complement inhibitors for therapeutic purposes and the use of circulating complement-protein measurement for patient stratification and are co-founders of and shareholders in Complement Therapeutics, a company that focuses on the development of complement-targeted therapeutics, including for AMD. The remaining authors declare no competing interests.

Received: January 10, 2021
Accepted: May 27, 2021
Published: July 13, 2021

Web resources

EPACTS, http://genome.sph.umich.edu/wiki/EPACTS
GTEx portal, https://gtexportal.org/home/
IAMDGC, http://eaglep.case.edu/iamdgc_web
LDlink, https://ldlink.nci.nih.gov/
PanoramaWeb, https://panoramaweb.org/
ProteomeXchange, http://proteomecentral.proteomexchange.org
Skyline, www.skyline.ms

References

1. Wong, W.L., Su, X., Li, X., Cheung, C.M., Klein, R., Cheng, C.Y., and Wong, T.Y. (2014). Global prevalence of age-related macular degeneration and disease burden projection for 2020 and 2040: a systematic review and meta-analysis. Lancet Glob. Health *2*, e106–e116.
2. Fritsche, L.G., Igl, W., Bailey, J.N.C., Grassmann, F., Sengupta, S., Bragg-Gresham, J.L., Burdon, K.P., Hebbring, S.J., Wen, C., Gorski, M., et al. (2016). A large genome-wide association study of age-related macular degeneration highlights contributions of rare and common variants. Nat. Genet. *48*, 134–143.
3. McHarg, S., Clark, S.J., Day, A.J., and Bishop, P.N. (2015). Age-related macular degeneration and the role of the complement system. Mol. Immunol. *67*, 43–50.
4. Weinberger, A.W., Eddahabi, C., Carstesen, D., Zipfel, P.F., Walter, P., and Skerka, C. (2014). Human complement factor H and factor H-like protein 1 are expressed in human retinal pigment epithelial cells. Ophthalmic Res. *51*, 59–66.
5. Cserhalmi, M., Papp, A., Brandus, B., Uzonyi, B., and Józsi, M. (2019). Regulation of regulators: Role of the complement factor H-related proteins. Semin. Immunol. *45*, 101341.
6. Keenan, T.D.L., Toso, M., Pappas, C., Nichols, L., Bishop, P.N., and Hageman, G.S. (2015). Assessment of Proteins Associated With Complement Activation and Inflammation in Maculae of Human Donors Homozygous Risk at Chromosome 1 CFH-to-F13B. Invest. Ophthalmol. Vis. Sci. *56*, 4870–4879.
7. Mullins, R.F., Schoo, D.P., Sohn, E.H., Flamme-Wiese, M.J., Workamelahu, G., Johnston, R.M., Wang, K., Tucker, B.A., and Stone, E.M. (2014). The membrane attack complex in aging human choriocapillaris: relationship to macular degeneration and choroidal thinning. Am. J. Pathol. *184*, 3142–3153.
8. Edwards, A.O., Ritter, R., 3rd, Abel, K.J., Manning, A., Panhuysen, C., and Farrer, L.A. (2005). Complement factor H polymorphism and age-related macular degeneration. Science *308*, 421–424.
9. Hageman, G.S., Anderson, D.H., Johnson, L.V., Hancox, L.S., Taiber, A.J., Hardisty, L.I., Hageman, J.L., Stockman, H.A., Borchardt, J.D., Gehrs, K.M., et al. (2005). A common haplotype in the complement regulatory gene factor H (HF1/CFH) predisposes individuals to age-related macular degeneration. Proc. Natl. Acad. Sci. USA *102*, 7227–7232.
10. Haines, J.L., Hauser, M.A., Schmidt, S., Scott, W.K., Olson, L.M., Gallins, P., Spencer, K.L., Kwan, S.Y., Noureddine, M., Gilbert, J.R., et al. (2005). Complement factor H variant increases the risk of age-related macular degeneration. Science *308*, 419–421.
11. Klein, R.J., Zeiss, C., Chew, E.Y., Tsai, J.Y., Sackler, R.S., Haynes, C., Henning, A.K., SanGiovanni, J.P., Mane, S.M., Mayne, S.T., et al. (2005). Complement factor H polymorphism in age-related macular degeneration. Science *308*, 385–389.
12. Hoffman, J.D., Cooke Bailey, J.N., D'Aoust, L., Cade, W., Ayala-Haedo, J., Fuzzell, D., Laux, R., Adams, L.D., Reinhart-Mercer, L., Caywood, L., et al. (2014). Rare complement factor H variant associated with age-related macular degeneration in the Amish. Invest. Ophthalmol. Vis. Sci. *55*, 4455–4460.
13. Raychaudhuri, S., Iartchouk, O., Chin, K., Tan, P.L., Tai, A.K., Ripke, S., Gowrisankar, S., Vemuri, S., Montgomery, K., Yu, Y., et al. (2011). A rare penetrant mutation in CFH confers high risk of age-related macular degeneration. Nat. Genet. *43*, 1232–1236.
14. Triebwasser, M.P., Roberson, E.D., Yu, Y., Schramm, E.C., Wagner, E.K., Raychaudhuri, S., Seddon, J.M., and Atkinson, J.P. (2015). Rare Variants in the Functional Domains of Complement Factor H Are Associated With Age-Related Macular Degeneration. Invest. Ophthalmol. Vis. Sci. *56*, 6873–6878.
15. Yu, Y., Triebwasser, M.P., Wong, E.K., Schramm, E.C., Thomas, B., Reynolds, R., Mardis, E.R., Atkinson, J.P., Daly, M., Raychaudhuri, S., et al. (2014). Whole-exome sequencing identifies rare, functional CFH variants in families with macular degeneration. Hum. Mol. Genet. *23*, 5283–5293.
16. Fritsche, L.G., Lauer, N., Hartmann, A., Stippa, S., Keilhauer, C.N., Oppermann, M., Pandey, M.K., Köhl, J., Zipfel, P.F., Weber, B.H., and Skerka, C. (2010). An imbalance of human complement regulatory proteins CFHR1, CFHR3 and factor H influences risk for age-related macular degeneration (AMD). Hum. Mol. Genet. *19*, 4694–4704.
17. Hughes, A.E., Orr, N., Esfandiary, H., Diaz-Torres, M., Goodship, T., and Chakravarthy, U. (2006). A common CFH haplotype, with deletion of CFHR1 and CFHR3, is associated with lower risk of age-related macular degeneration. Nat. Genet. *38*, 1173–1177.

18. Kubista, K.E., Tosakulwong, N., Wu, Y., Ryu, E., Roeder, J.L., Hecker, L.A., Baratz, K.H., Brown, W.L., and Edwards, A.O. (2011). Copy number variation in the complement factor H-related genes and age-related macular degeneration. Mol. Vis. *17*, 2080–2092.
19. Raychaudhuri, S., Ripke, S., Li, M., Neale, B.M., Fagerness, J., Reynolds, R., Sobrin, L., Swaroop, A., Abecasis, G., Seddon, J.M., and Daly, M.J. (2010). Associations of CFHR1-CFHR3 deletion and a CFH SNP to age-related macular degeneration are not independent. Nat. Genet. *42*, 553–555, author reply 555–556.
20. Sivakumaran, T.A., Igo, R.P., Jr., Kidd, J.M., Itsara, A., Kopplin, L.J., Chen, W., Hagstrom, S.A., Peachey, N.S., Francis, P.J., Klein, M.L., et al. (2011). A 32 kb critical region excluding Y402H in CFH mediates risk for age-related macular degeneration. PLoS ONE *6*, e25598.
21. Spencer, K.L., Hauser, M.A., Olson, L.M., Schmidt, S., Scott, W.K., Gallins, P., Agarwal, A., Postel, E.A., Pericak-Vance, M.A., and Haines, J.L. (2008). Deletion of CFHR3 and CFHR1 genes in age-related macular degeneration. Hum. Mol. Genet. *17*, 971–977.
22. Hageman, G.S., Hancox, L.S., Taiber, A.J., Gehrs, K.M., Anderson, D.H., Johnson, L.V., Radeke, M.J., Kavanagh, D., Richards, A., Atkinson, J., et al. (2006). Extended haplotypes in the complement factor H (CFH) and CFH-related (CFHR) family of genes protect against age-related macular degeneration: Characterization, ethnic distribution and evolutionary implications. Ann. Med. *38*, 592–604.
23. Visscher, P.M., Wray, N.R., Zhang, Q., Sklar, P., McCarthy, M.I., Brown, M.A., and Yang, J. (2017). 10 Years of GWAS Discovery: Biology, Function, and Translation. Am. J. Hum. Genet. *101*, 5–22.
24. Cipriani, V., Lorés-Motta, L., He, F., Fathalla, D., Tilakaratna, V., McHarg, S., Bayatti, N., Acar, I.E., Hoyng, C.B., Fauser, S., et al. (2020). Increased circulating levels of Factor H-Related Protein 4 are strongly associated with age-related macular degeneration. Nat. Commun. *11*, 778.
25. Clark, S.J., and Bishop, P.N. (2015). Role of Factor H and Related Proteins in Regulating Complement Activation in the Macula, and Relevance to Age-Related Macular Degeneration. J. Clin. Med. *4*, 18–31.
26. Cipriani, V., Leung, H.T., Plagnol, V., Bunce, C., Khan, J.C., Shahid, H., Moore, A.T., Harding, S.P., Bishop, P.N., Hayward, C., et al.; French AMD Investigators (2012). Genome-wide association study of age-related macular degeneration identifies associated variants in the TNXB-FKBPL-NOTCH4 region of chromosome 6p21.3. Hum. Mol. Genet. *21*, 4138–4150.
27. Yates, J.R.W., Sepp, T., Matharu, B.K., Khan, J.C., Thurlby, D.A., Shahid, H., Clayton, D.G., Hayward, C., Morgan, J., Wright, A.F., et al.; Genetic Factors in AMD Study Group (2007). Complement C3 variant and the risk of age-related macular degeneration. N. Engl. J. Med. *357*, 553–561.
28. Bird, A.C., Bressler, N.M., Bressler, S.B., Chisholm, I.H., Coscas, G., Davis, M.D., de Jong, P.T.V.M., Klaver, C.C.W., Klein, B.E.K., Klein, R., et al.; The International ARM Epidemiological Study Group (1995). An international classification and grading system for age-related maculopathy and age-related macular degeneration. Surv. Ophthalmol. *39*, 367–374.
29. Pino, L.K., Searle, B.C., Bollinger, J.G., Nunn, B., MacLean, B., and MacCoss, M.J. (2020). The Skyline ecosystem: Informatics for quantitative mass spectrometry proteomics. Mass Spectrom. Rev. *39*, 229–244.
30. Abecasis, G.R., Auton, A., Brooks, L.D., DePristo, M.A., Durbin, R.M., Handsaker, R.E., Kang, H.M., Marth, G.T., McVean, G.A.; and 1000 Genomes Project Consortium (2012). An integrated map of genetic variation from 1,092 human genomes. Nature *491*, 56–65.
31. Yang, J., Ferreira, T., Morris, A.P., Medland, S.E., Madden, P.A.F., Heath, A.C., Martin, N.G., Montgomery, G.W., Weedon, M.N., Loos, R.J., et al.; Genetic Investigation of ANthropometric Traits (GIANT) Consortium; and DIAbetes Genetics Replication And Meta-analysis (DIAGRAM) Consortium (2012). Conditional and joint multiple-SNP analysis of GWAS summary statistics identifies additional variants influencing complex traits. Nat. Genet. *44*, 369–375, S1–S3.
32. Bowden, J., and Vansteelandt, S. (2011). Mendelian randomization analysis of case-control data using structural mean models. Stat. Med. *30*, 678–694.
33. Burgess, S., Small, D.S., and Thompson, S.G. (2017). A review of instrumental variable estimators for Mendelian randomization. Stat. Methods Med. Res. *26*, 2333–2355.
34. Clark, S.J., and Bishop, P.N. (2018). The eye as a complement dysregulation hotspot. Semin. Immunopathol. *40*, 65–74.
35. Clark, S.J., Perveen, R., Hakobyan, S., Morgan, B.P., Sim, R.B., Bishop, P.N., and Day, A.J. (2010). Impaired binding of the age-related macular degeneration-associated complement factor H 402H allotype to Bruch's membrane in human retina. J. Biol. Chem. *285*, 30192–30202.
36. Clark, S.J., Schmidt, C.Q., White, A.M., Hakobyan, S., Morgan, B.P., and Bishop, P.N. (2014). Identification of factor H-like protein 1 as the predominant complement regulator in Bruch's membrane: implications for age-related macular degeneration. J. Immunol. *193*, 4962–4970.
37. Zhang, P., Zhu, M., Geng-Spyropoulos, M., Shardell, M., Gonzalez-Freire, M., Gudnason, V., Eiriksdottir, G., Schaumberg, D., Van Eyk, J.E., Ferrucci, L., and Semba, R.D. (2017). A novel, multiplexed targeted mass spectrometry assay for quantification of complement factor H (CFH) variants and CFH-related proteins 1-5 in human plasma. Proteomics *17*.
38. Davies, N.M., Holmes, M.V., and Davey Smith, G. (2018). Reading Mendelian randomisation studies: a guide, glossary, and checklist for clinicians. BMJ *362*, k601.
39. Walker, V.M., Davey Smith, G., Davies, N.M., and Martin, R.M. (2017). Mendelian randomization: a novel approach for the prediction of adverse drug events and drug repurposing opportunities. Int. J. Epidemiol. *46*, 2078–2089.
40. Burgess, S., Foley, C.N., and Zuber, V. (2018). Inferring Causal Relationships Between Risk Factors and Outcomes from Genome-Wide Association Study Data. Annu. Rev. Genomics Hum. Genet. *19*, 303–327.
41. Foley, C.N., Staley, J.R., Breen, P.G., Sun, B.B., Kirk, P.D.W., Burgess, S., and Howson, J.M.M. (2021). A fast and efficient colocalization algorithm for identifying shared genetic risk factors across multiple traits. Nat. Commun. *12*, 764.
42. Hormozdiari, F., van de Bunt, M., Segrè, A.V., Li, X., Joo, J.W.J., Bilow, M., Sul, J.H., Sankararaman, S., Pasaniuc, B., and Eskin, E. (2016). Colocalization of GWAS and eQTL Signals Detects Target Genes. Am. J. Hum. Genet. *99*, 1245–1260.
43. Hukku, A., Pividori, M., Luca, F., Pique-Regi, R., Im, H.K., and Wen, X. (2021). Probabilistic colocalization of genetic variants

from complex and molecular traits: promise and limitations. Am. J. Hum. Genet. *108*, 25–35.

44. Wallace, C. (2020). Eliciting priors and relaxing the single causal variant assumption in colocalisation analyses. PLoS Genet. *16*, e1008720.

45. Zheng, J., Haberland, V., Baird, D., Walker, V., Haycock, P.C., Hurle, M.R., Gutteridge, A., Erola, P., Liu, Y., Luo, S., et al. (2020). Phenome-wide Mendelian randomization mapping the influence of the plasma proteome on complex diseases. Nat. Genet. *52*, 1122–1131.

46. Consortium, G.T.; and GTEx Consortium (2015). Human genomics. The Genotype-Tissue Expression (GTEx) pilot analysis: multitissue gene regulation in humans. Science *348*, 648–660.

The American Journal of Human Genetics, Volume *108*

Supplemental information

Beyond factor H: The impact of genetic-risk variants for age-related macular degeneration on circulating factor-H-like 1 and factor-H-related protein concentrations

Valentina Cipriani, Anna Tierney, John R. Griffiths, Verena Zuber, Panagiotis I. Sergouniotis, John R.W. Yates, Anthony T. Moore, Paul N. Bishop, Simon J. Clark, and Richard D. Unwin

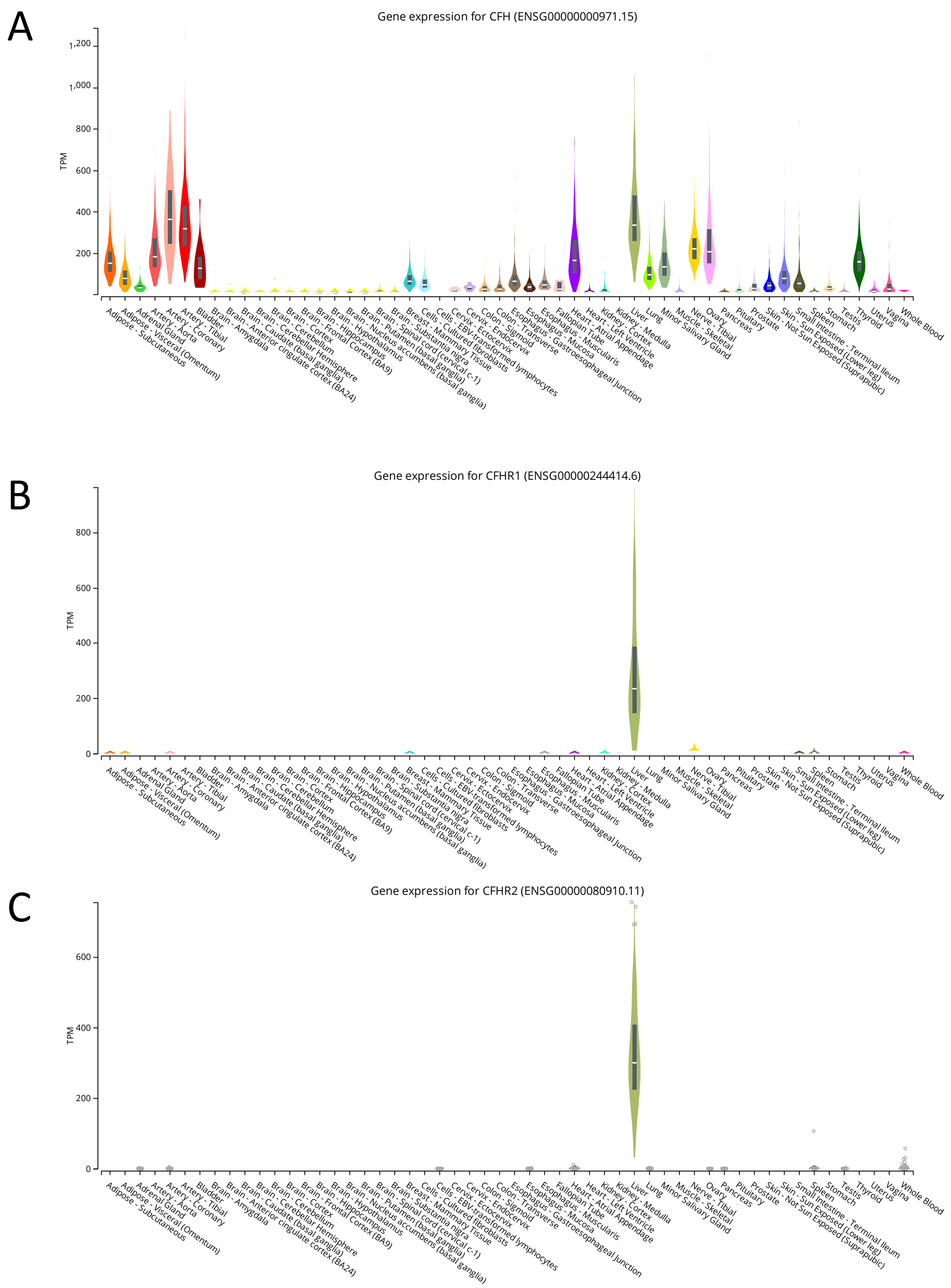

Figure S1. Gene expression of *CFHR1*, *CFHR2*, *CFHR3*, *CFHR4* and *CFHR5* is restricted to liver.

RNA sequencing of 54 human tissue samples from the Genotype-Tissue Expression (GTEx) project[1] (https://gtexportal.org/home/multiGeneQueryPage; dataset dbGaP accession number phs000424.v8.p2) detected *CFHR1* (B), *CFHR2* (C), *CFHR3* (D), *CFHR4* (E) and *CFHR5* (F) expression exclusively in the liver, whereas *CFH* (A) expression was more widespread.

(continued on the next page)

D

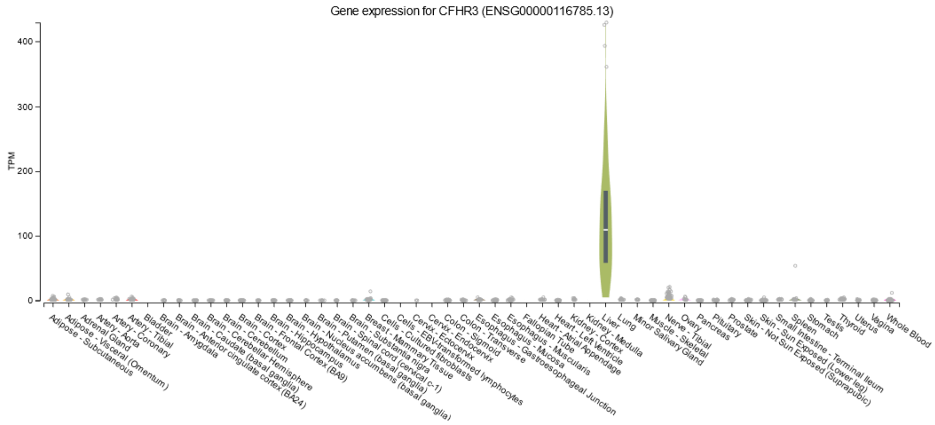
Gene expression for CFHR3 (ENSG00000116785.13)
TPM
400
300
200
100
0
Adipose - Subcutaneous
Adipose - Visceral (Omentum)
Adrenal Gland
Artery - Aorta
Artery - Coronary
Artery - Tibial
Bladder
Brain - Amygdala
Brain - Anterior cingulate cortex (BA24)
Brain - Caudate (basal ganglia)
Brain - Cerebellar Hemisphere
Brain - Cerebellum
Brain - Cortex
Brain - Frontal Cortex (BA9)
Brain - Hippocampus
Brain - Hypothalamus
Brain - Nucleus accumbens (basal ganglia)
Brain - Putamen (basal ganglia)
Brain - Spinal cord (cervical c-1)
Brain - Substantia nigra
Breast - Mammary Tissue
Cells - Cultured fibroblasts
Cells - EBV-transformed lymphocytes
Cervix - Ectocervix
Cervix - Endocervix
Colon - Sigmoid
Colon - Transverse
Esophagus - Gastroesophageal Junction
Esophagus - Mucosa
Esophagus - Muscularis
Fallopian Tube
Heart - Atrial Appendage
Heart - Left Ventricle
Kidney - Cortex
Kidney - Medulla
Liver
Lung
Minor Salivary Gland
Muscle - Skeletal
Nerve - Tibial
Ovary
Pancreas
Pituitary
Prostate
Skin - Not Sun Exposed (Suprapubic)
Skin - Sun Exposed (Lower leg)
Small Intestine - Terminal Ileum
Spleen
Stomach
Testis
Thyroid
Uterus
Vagina
Whole Blood

E

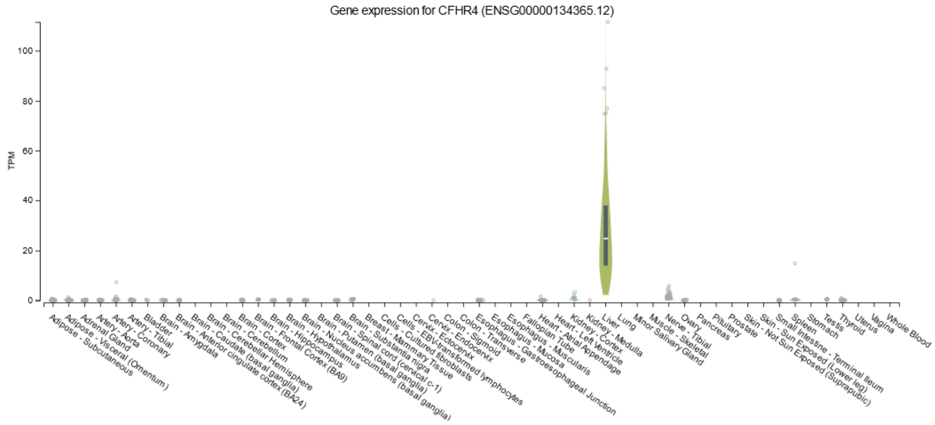
Gene expression for CFHR4 (ENSG00000134365.12)
TPM
100
80
60
40
20
0
Adipose - Subcutaneous
Adipose - Visceral (Omentum)
Adrenal Gland
Artery - Aorta
Artery - Coronary
Artery - Tibial
Bladder
Brain - Amygdala
Brain - Anterior cingulate cortex (BA24)
Brain - Caudate (basal ganglia)
Brain - Cerebellar Hemisphere
Brain - Cerebellum
Brain - Cortex
Brain - Frontal Cortex (BA9)
Brain - Hippocampus
Brain - Hypothalamus
Brain - Nucleus accumbens (basal ganglia)
Brain - Putamen (basal ganglia)
Brain - Spinal cord (cervical c-1)
Brain - Substantia nigra
Breast - Mammary Tissue
Cells - Cultured fibroblasts
Cells - EBV-transformed lymphocytes
Cervix - Ectocervix
Cervix - Endocervix
Colon - Sigmoid
Colon - Transverse
Esophagus - Gastroesophageal Junction
Esophagus - Mucosa
Esophagus - Muscularis
Fallopian Tube
Heart - Atrial Appendage
Heart - Left Ventricle
Kidney - Cortex
Kidney - Medulla
Liver
Lung
Minor Salivary Gland
Muscle - Skeletal
Nerve - Tibial
Ovary
Pancreas
Pituitary
Prostate
Skin - Not Sun Exposed (Suprapubic)
Skin - Sun Exposed (Lower leg)
Small Intestine - Terminal Ileum
Spleen
Stomach
Testis
Thyroid
Uterus
Vagina
Whole Blood

F

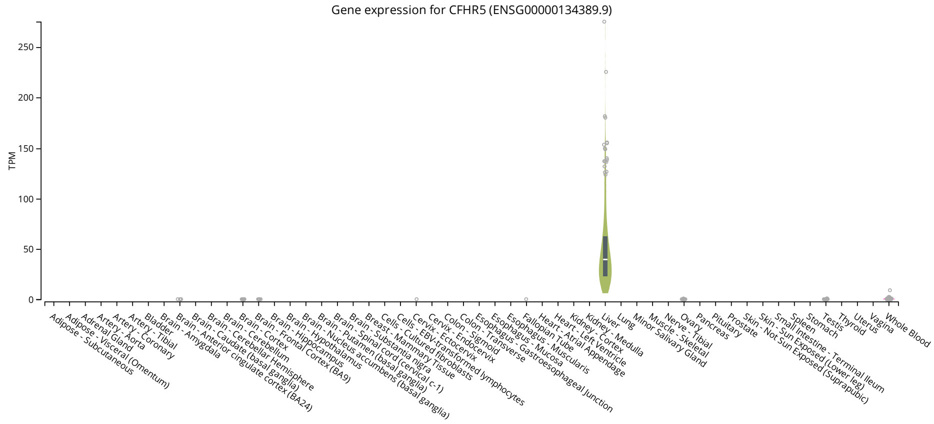
Gene expression for CFHR5 (ENSG00000134389.9)
TPM
250
200
150
100
50
0
Adipose - Subcutaneous
Adipose - Visceral (Omentum)
Adrenal Gland
Artery - Aorta
Artery - Coronary
Artery - Tibial
Bladder
Brain - Amygdala
Brain - Anterior cingulate cortex (BA24)
Brain - Caudate (basal ganglia)
Brain - Cerebellar Hemisphere
Brain - Cerebellum
Brain - Cortex
Brain - Frontal Cortex (BA9)
Brain - Hippocampus
Brain - Hypothalamus
Brain - Nucleus accumbens (basal ganglia)
Brain - Putamen (basal ganglia)
Brain - Spinal cord (cervical c-1)
Brain - Substantia nigra
Breast - Mammary Tissue
Cells - Cultured fibroblasts
Cells - EBV-transformed lymphocytes
Cervix - Ectocervix
Cervix - Endocervix
Colon - Sigmoid
Colon - Transverse
Esophagus - Gastroesophageal Junction
Esophagus - Mucosa
Esophagus - Muscularis
Fallopian Tube
Heart - Atrial Appendage
Heart - Left Ventricle
Kidney - Cortex
Kidney - Medulla
Liver
Lung
Minor Salivary Gland
Muscle - Skeletal
Nerve - Tibial
Ovary
Pancreas
Pituitary
Prostate
Skin - Not Sun Exposed (Suprapubic)
Skin - Sun Exposed (Lower leg)
Small Intestine - Terminal Ileum
Spleen
Stomach
Testis
Thyroid
Uterus
Vagina
Whole Blood

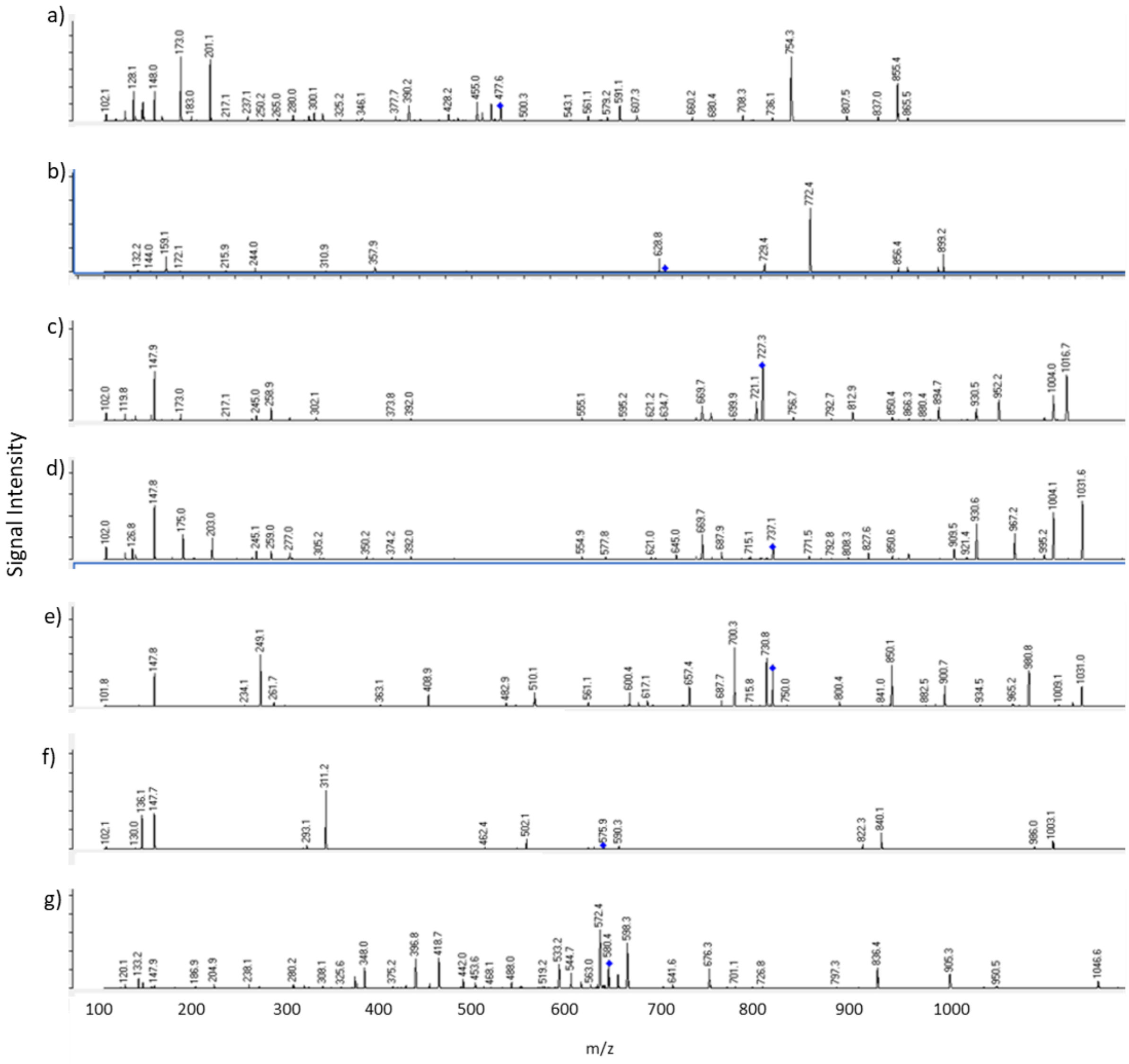

Figure S2. MS/MS fragmentation spectra of all proteotypic peptides used for quantification of FH, FHL-1 and FHR-1 to FHR-5 proteins in human samples.

a) VTYKcFE (FH), b) NGWSPTPRcIRVSFTL (FHL-1), c) ATFcDFPKINHGILYDEE (FHR-1), d) AMFcDFPKINHGILYDEE (FHR-2), e) VAcHPGYGLPKAQTTVTcTE (FHR-3), f) YQcQSYYE (FHR-4), and g) RGWSTPPIcSFTKGE (FHR-5).

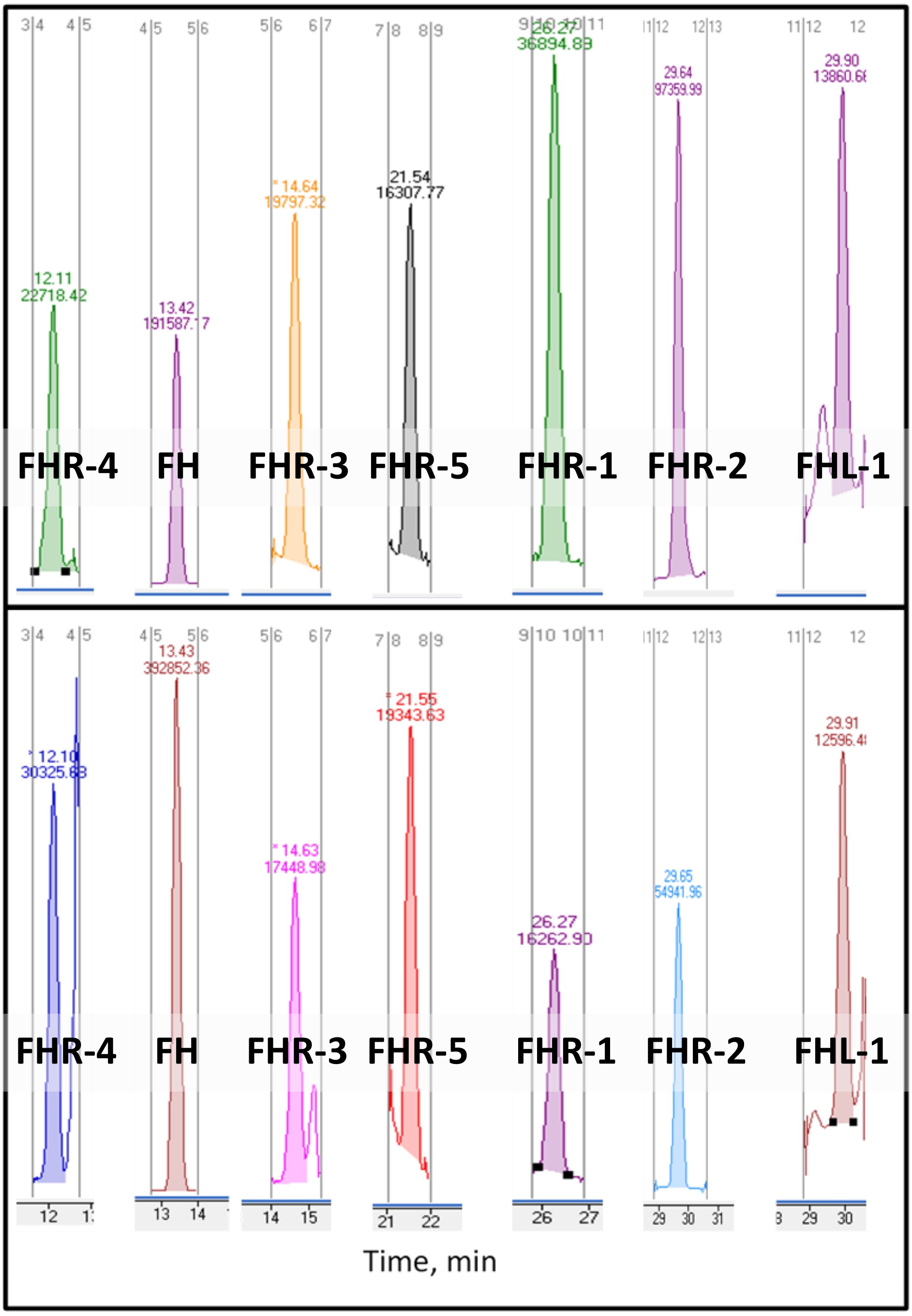

Figure S3. Overlay of endogenous and stable isotope-labelled standard peptide SRM signals.

To confirm assay specificity, stable isotope-labelled peptides were spiked into plasma and the elution profiles of each of the heavy:light pairs was compared to confirm specificity of the individual SRMs for each peptide. Upper panel shows signals from endogenous peptides, while the lower panel shows the equivalent SIS peptide.

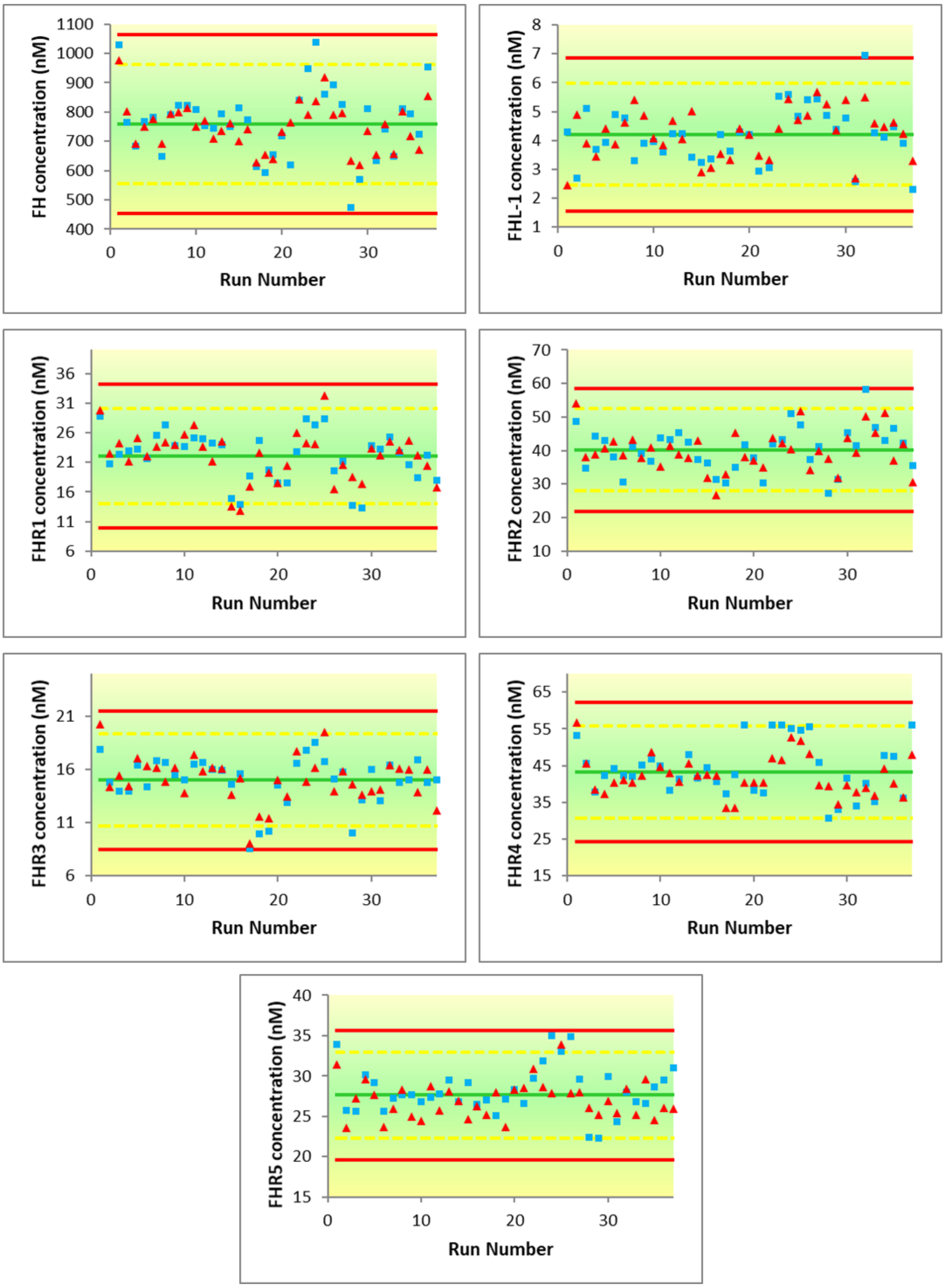

Figure S4. Levy-Jennings graphs to monitor between-batch stability of the whole process across the course of the study.

Measured concentrations for each protein in two replicate analyses of the same sample included in each batch were monitored. Green line = mean concentration, Yellow line = +/- 2x s.d., Red line = +/-3x s.d.

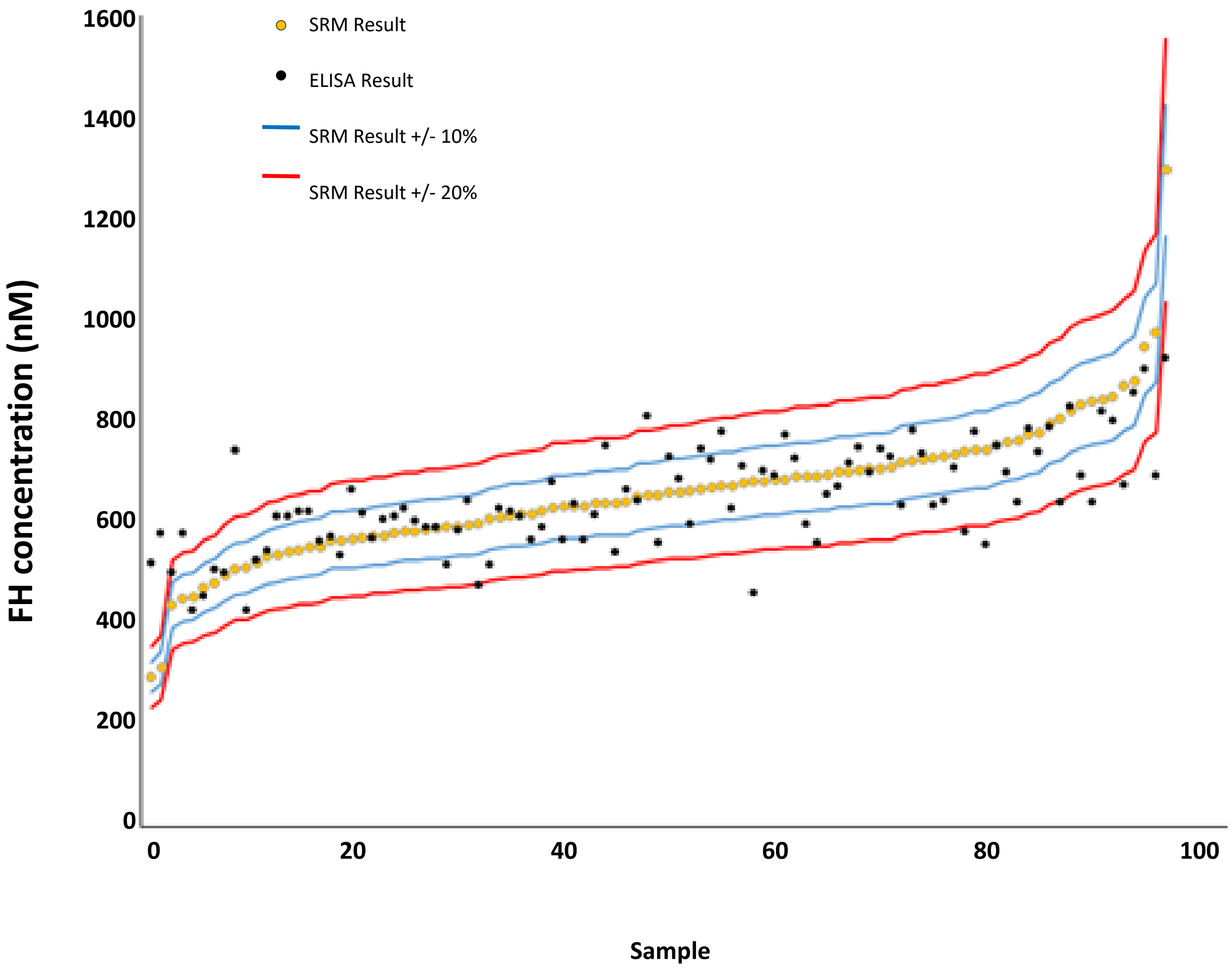

Figure S5. Correlation between measured concentrations of FH using the LS-MS based assay and immunoassay.

Immunoassay-derived concentrations were normalised to match the median concentration calculated by the SRM. In most case ELISA measurements are within 20% of the SRM measurement.

A

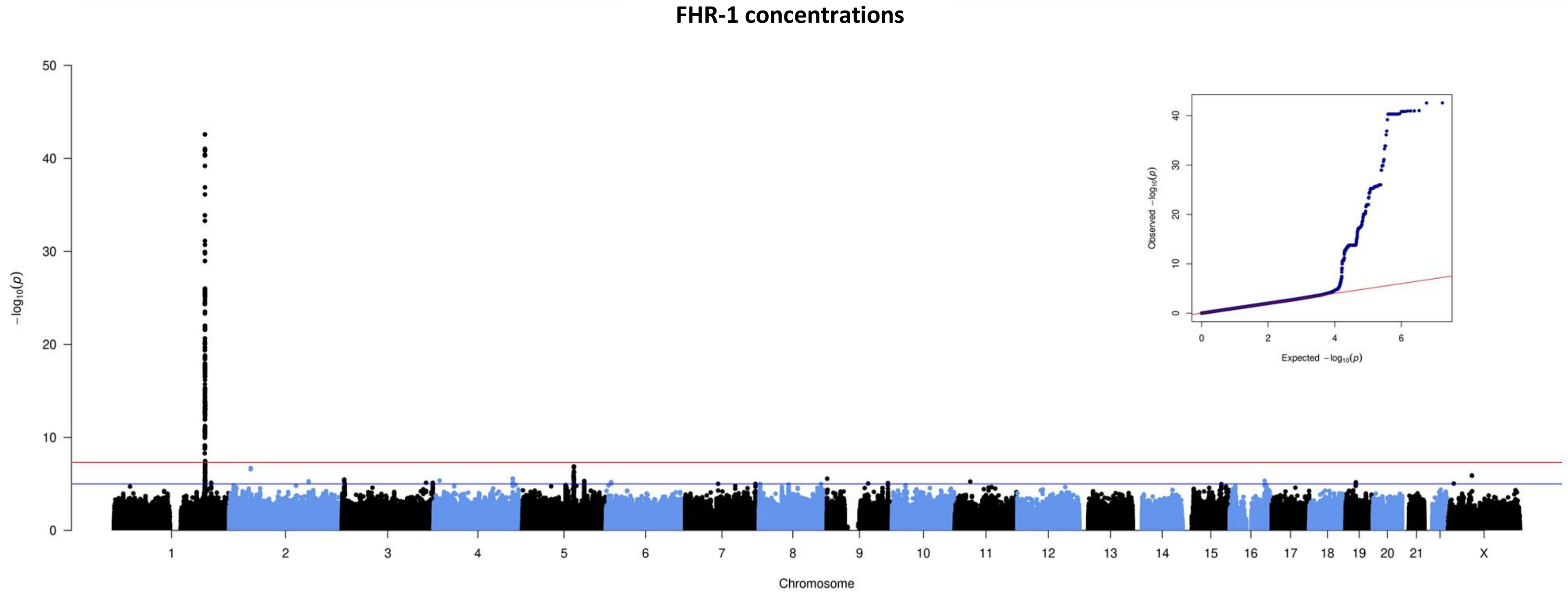

Figure S6. GWASs of circulating FHR-1, FHR-2, FHR-3, FHR-4, FHR-5 protein concentrations in 252 controls from the Cambridge AMD cohort reveal a strong genome-wide significant signal spanning the AMD-associated *CFH* locus on chromosome 1q31.3.

Manhattan plot together with quantile-quantile (QQ) plot (upper right-hand side of each panel) for the GWAS of FHR-1 (A), FHR-2 (B), FHR-3 (C), FHR-4 (D), FHR-5 (E), FH (F) and FHL-1 (G) protein concentrations. Manhattan plots illustrate P-values for each single variant tested for association with the protein concentrations. Observed -$\log_{10}$(P-values) are plotted against the genomic position of each variant on chromosomes 1–22. The horizontal red line indicates the threshold considered for genome-wide significance (P-value $\leq 5 \times 10^{-8}$). QQ plots compare the distribution of the observed test statistics with its expected distribution under the null hypothesis of no association. Genomic control values (λ)

calculated based on the 50th percentile (and 1/10th of a percentile) were equal to 1.010 (1.004), 1.014 (1.026), 0.983 (1.074), 1.012 (1.025), 0.994 (1.014), 0.991 (1.018) and 0.995 (0.998) for FHR-1, FHR-2, FHR-3, FHR-4, FHR-5, FH and FHL-1, respectively.

(continued on the next page)

B

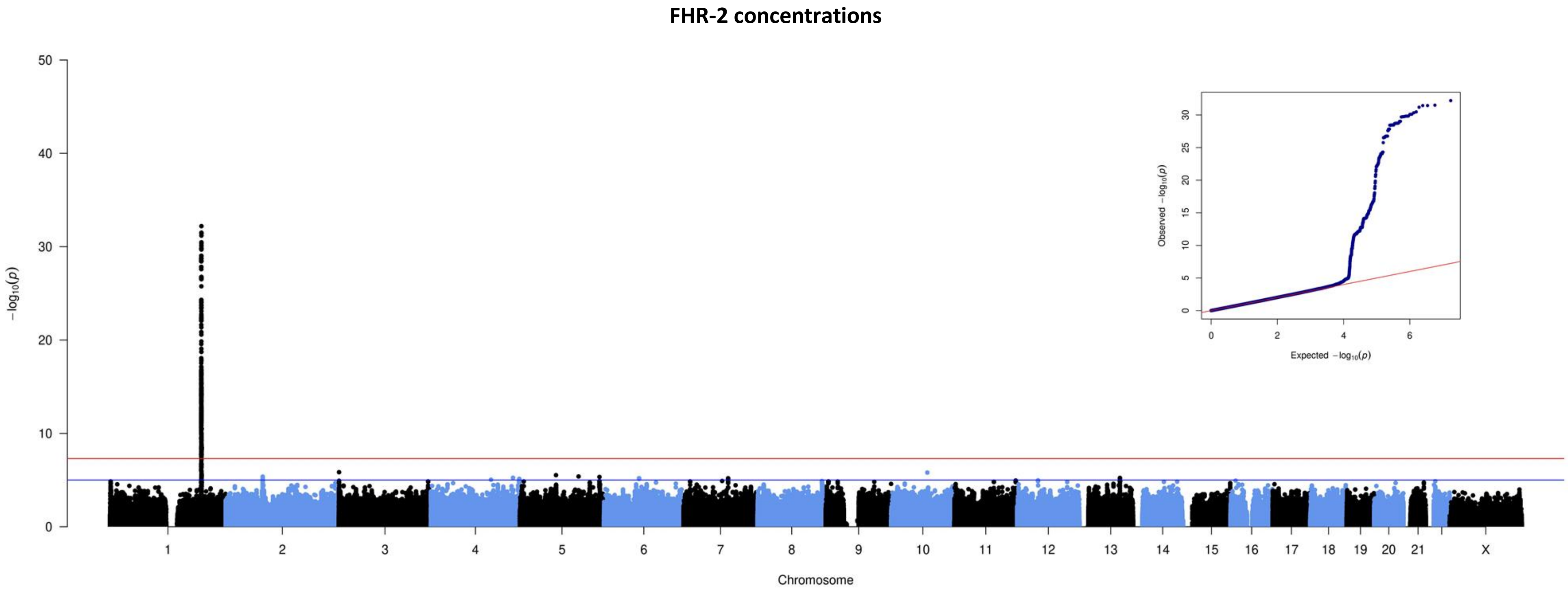

(continued on the next page)

C

FHR-3 concentrations

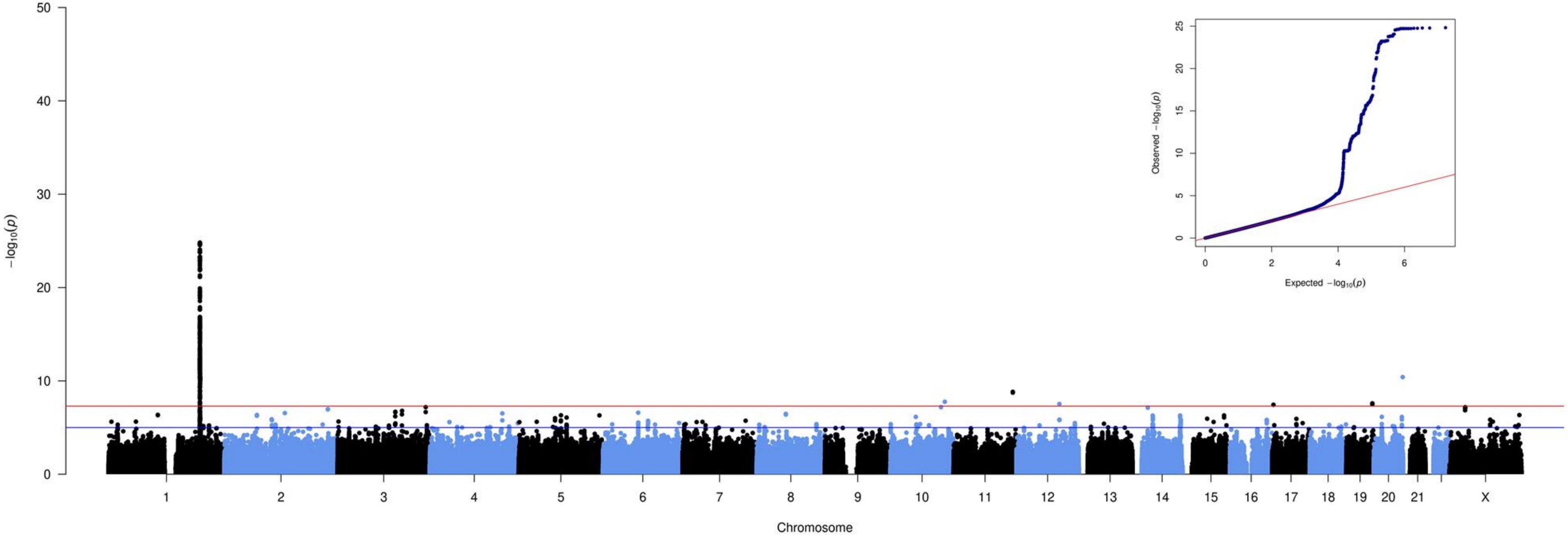

(continued on the next page)

D

FHR-4 concentrations

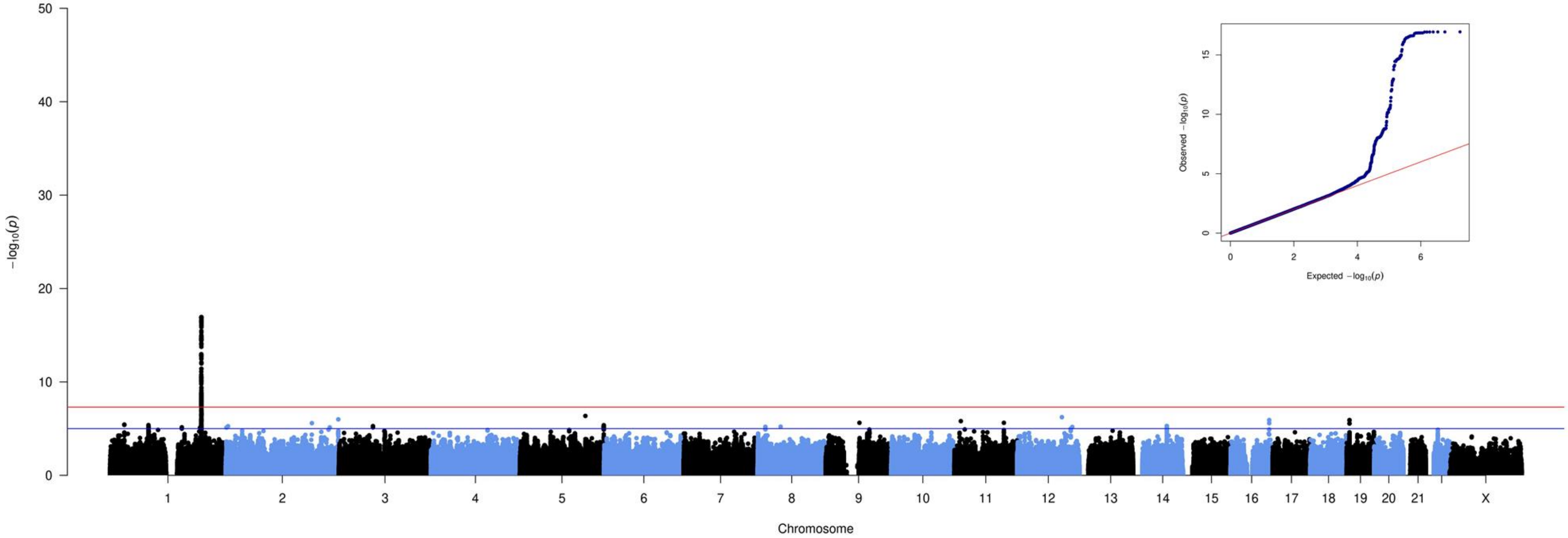

(continued on the next page)

E

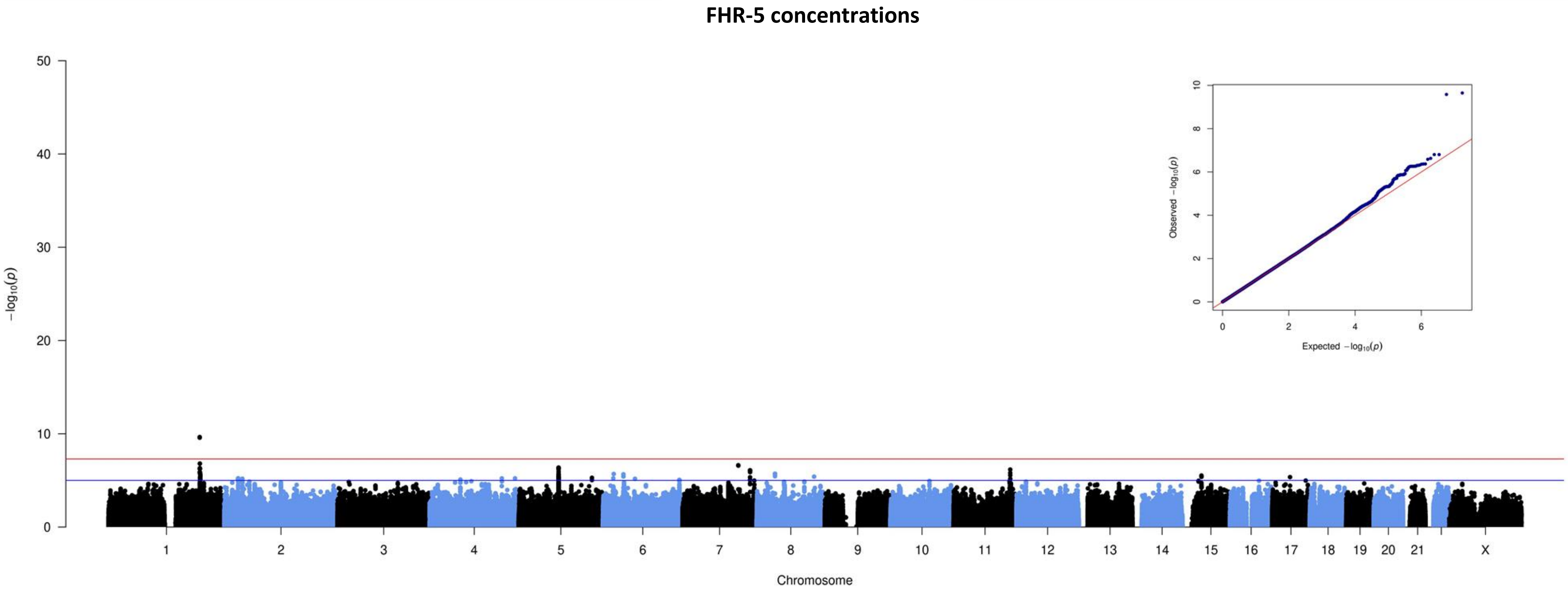

(continued on the next page)

F

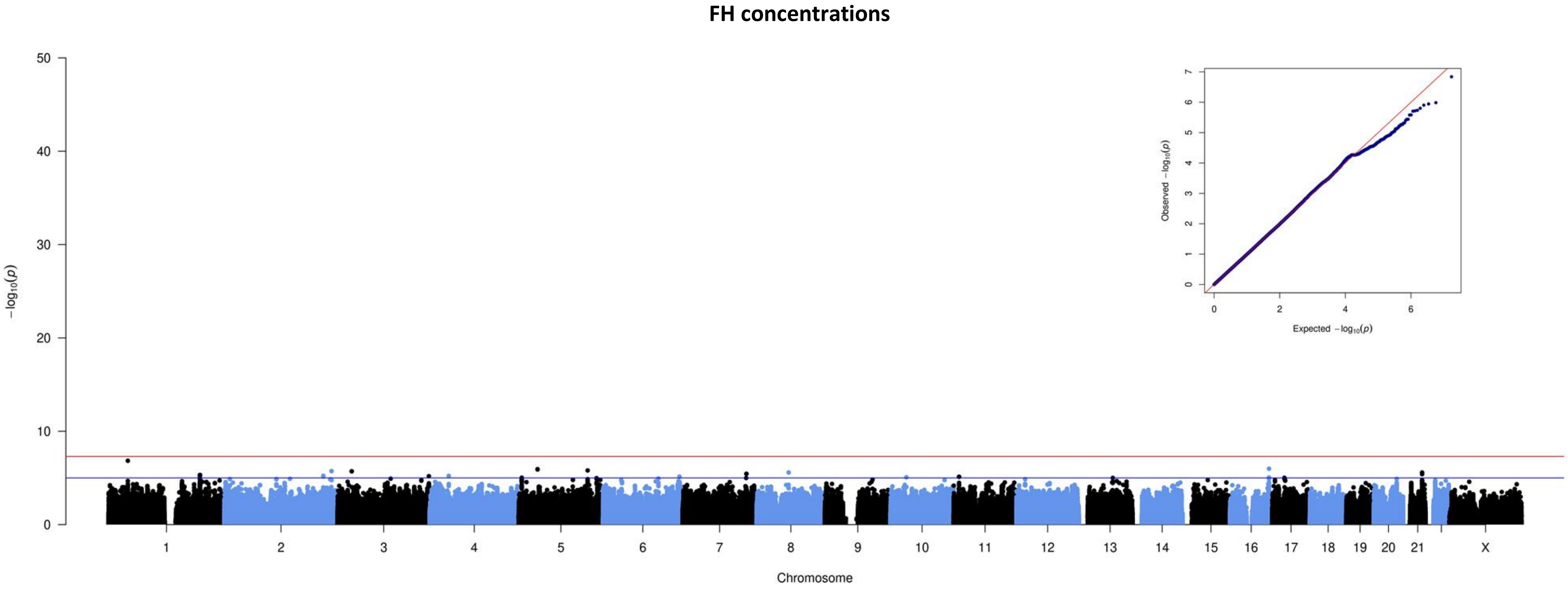

(continued on the next page)

G

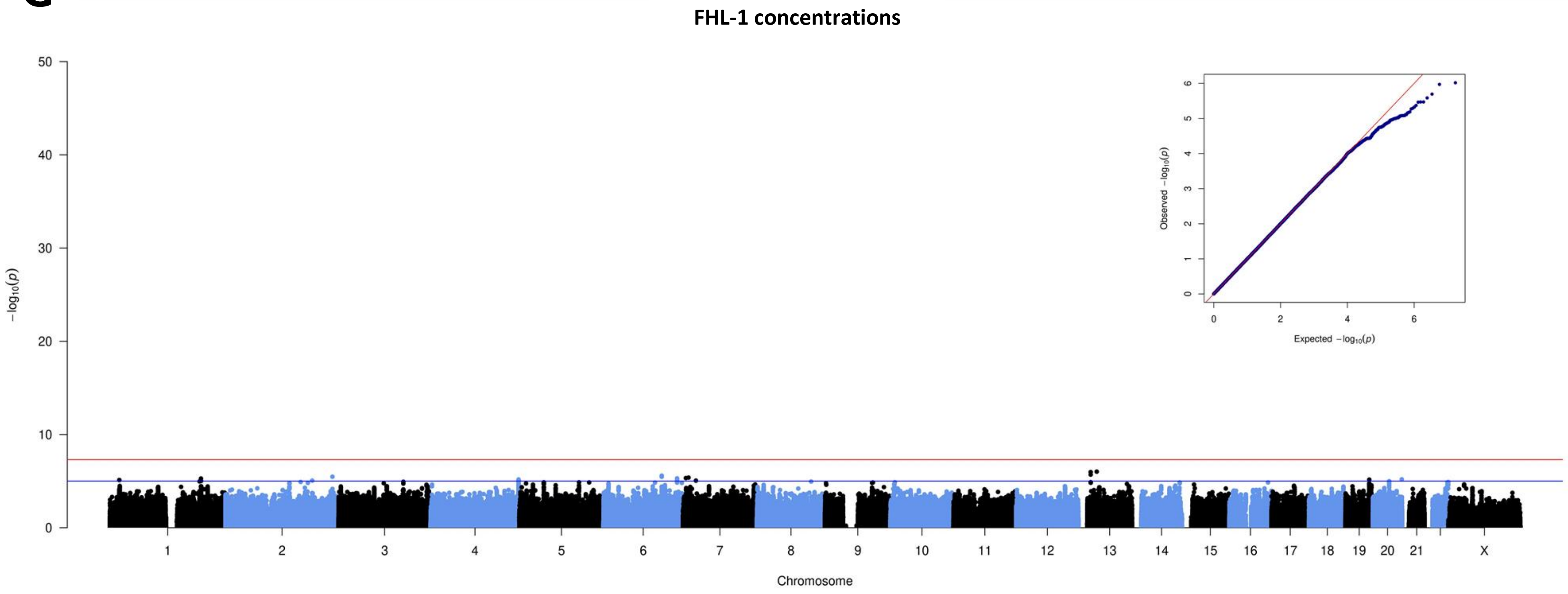
FHL-1 concentrations
50
40
30
20
10
0
−log10(p)
1
2
3
4
5
6
7
8
9
10
11
12
13
14
15
16
17
18
19
20
21
X
Chromosome
Observed −log10(p)
Expected −log10(p)

Parameter	Value
Gas Temp	210 °C
Gas Flow	15 l/min
Nebuliser	30 psi
Sheath Gas Temp	250 °C
Sheath Gas Flow	12 l/min
Capillary Voltage	2650 V
Nozzle Voltage	1000 V
High Pressure RF	200 V
Low Pressure RF	110 V

Table S1. Liquid chromatography-selected reaction monitoring mass spectrometry (LC-SRM-MS) instrument parameters.

Protein	Peptide Sequence	Precursor ion m/z	Product ions m/z	Collision energy, eV	Dwell time, ms
**FH**	VTY**K**cFE (Light)	473.7	583.3, 847.4, 746.3	16, 16, 16	400, 200, 150
	VTY**K**cFE (Heavy)	477.7	591.3, 855.4, 754.3	16, 16, 16	400, 200, 150
**FHL-1**	NGWSPTP**R**cIRVSFTL (Light)	631.2	723.9, 860.5, 767.4	19, 19, 19	150, 200, 100
	NGWSPTP**R**cIRVSFTL (Heavy)	634.3	728.9, 865.5, 772.4	19, 19, 19	150, 200, 100
**FHR-1**	ATFcD**F**PKINHGILYDEE (Light)	724.2	925.6, 1011.9, 947.1	20, 16, 20	400, 200, 200
	ATFcD**F**PKINHGILYDEE (Heavy)	727.2	930.6, 1016.9, 952.1	20, 16, 20	400, 200, 200
**FHR-2**	AMFcD**F**PKINHGILYDEE (Light)	734.0	999.5, 925.9, 1027	18,22,18	150, 125, 100
	AMFcD**F**PKINHGILYDEE (Heavy)	737.3	1004.5, 930.9, 1032	18,22,18	150, 125, 100
**FHR-3**	VAcHPG**Y**GLP**K**AQTTVTcTE (Light)	730.7	1022.4, 971.7	16, 18	350, 400
	VAcHPG**Y**GLP**K**AQTTVTcTE (Heavy)	736.7	1031.4, 980.7	16, 18	350, 400
**FHR-4**	**Y**QcQSYYE (Light)	570.7	830.3, 993.1, 311.1	11, 10, 14	250, 250, 250
	**Y**QcQSYYE (Heavy)	575.7	840.3, 1003.1, 311.1	11, 10, 14	250, 250, 250
**FHR-5**	**R**GWSTPPIcSFT**K**GE (Light)	575.2	828.4, 895.5, 588.3	16, 15, 20	200, 350, 200
	**R**GWSTPPIcSFT**K**GE (Heavy)	581.2	836.4, 905.5, 598.3	16, 15, 20	200, 350, 200

Table S2. SRM transition parameters.

Protein	Instrumental variable (IV) dbSNP ID (Chr:Position)[a] Non effect allele/Effect allele	*cis* / *trans* pQTL	IV strength (R^2)[b]	Association with protein concentrations in 252 Cambridge AMD study[2; 3] controls			Association with AMD in the Cambridge AMD GWAS[2; 3] (845 AMD cases and 419 controls)			Association with AMD in the IAMDGC GWAS[4] (16,144 AMD cases and 17,832 controls)			
				Beta	SE	P-value	Beta	SE	P-value	Beta	SE	P-value	Minor Allele Frequency
**FHR-2**	rs79351096 1:196918741_G/A (*CFHR2* nonsynonymous)	*cis*	0.09	-1.81	0.36	1.2×10^{-6}	-0.37	0.29	0.207	-0.46	0.06	1.0×10^{-13}	0.019
**FHR-3**	rs16840522 1:196710916_T/C (*CFH* intronic)	*cis*	0.35	-1.79	0.16	6.1×10^{-24}	-0.74	0.125	4.6×10^{-9}	-0.86	0.025	5.6×10^{-292}	0.158
**FHR-4**	rs34538561 1:196534406_C/G (*KCNT2* intronic)	*cis*	0.12	-1.63	0.28	2.5×10^{-8}	0.56	0.14	4.0×10^{-5}	0.51	0.03	7.8×10^{-92}	0.132

Table S7. Additional instrumental variables (IVs) for FHR-2, FHR-3 and FHR-4 identified at the *CFH* locus using the GCTA-COJO[5] approach.

[a]Chromosomal position is given according to the NCBI RefSeq hg19 human genome reference assembly; [b]The strength of each IV was evaluated using R^2 as the proportion of the variance of the protein explained by the genetic variant (function *get_r_from_pn* from R package *TwoSampleMR*, version 0.5.5).

The GCTA-COJO[5] approach was applied with default settings; the available individual-level genotype data from the entire control set in the Cambridge AMD study,[2; 3] n = 419, was used as a reference sample to estimate LD among genetic variants.

AMD = Age-Related macular degeneration; GWAS = Genome-wide association study; IAMDGC = International Age-Related Macular Degeneration Genomics Consortium; pQTL = protein quantitative trait locus.

Supplemental Note

List of the International Age-related Macular Degeneration Genomics Consortium (IAMDGC) members

The list reflects the author list of the previous IAMDGC publication by Fritsche *et al.*, 2016.[4]

Lars G Fritsche[1], Wilmar Igl[2], Jessica N Cooke Bailey[3], Felix Grassmann[4], Sebanti Sengupta[1], Jennifer L Bragg-Gresham[1,5], Kathryn P Burdon[6], Scott J Hebbring[7], Cindy Wen[8], Mathias Gorski[2], Ivana K Kim[9], David Cho[10], Donald Zack[11-15], Eric Souied[16], Hendrik P N Scholl[11,17], Elisa Bala[18], Kristine E Lee[19], David J Hunter[20,21], Rebecca J Sardell[22], Paul Mitchell[23], Joanna E Merriam[24], Valentina Cipriani[25,26], Joshua D Hoffman[27], Tina Schick[28], Yara T E Lechanteur[29], Robyn H Guymer[30], Matthew P Johnson[31], Yingda Jiang[32], Chloe M Stanton[33], Gabriëlle H S Buitendijk[34,35], Xiaowei Zhan[1,36,37], Alan M Kwong[1], Alexis Boleda[38], Matthew Brooks[38], Linn Gieser[38], Rinki Ratnapriya[38], Kari E Branham[39], Johanna R Foerster[1], John R Heckenlively[39], Mohammad I Othman[39], Brendan J Vote[6], Helena Hai Liang[30], Emmanuelle Souzeau[40], Ian L McAllister[41], Timothy Isaacs[41], Janette Hall[40], Stewart Lake[40], David A Mackey[6,30,41], Ian J Constable[41], Jamie E Craig[40], Terrie E Kitchner[7], Zhenglin Yang[42,43], Zhiguang Su[44], Hongrong Luo[8], Daniel Chen[8], Hong Ouyang[8], Ken Flagg[8], Danni Lin[8], Guanping Mao[8], Henry Ferreyra[8], Klaus Stark[2], Claudia N von Strachwitz[45], Armin Wolf[46], Caroline Brandl[2,4,47], Guenther Rudolph[46], Matthias Olden[2], Margaux A Morrison[48], Denise J Morgan[48], Matthew Schu[49-53], Jeeyun Ahn[54], Giuliana Silvestri[55], Evangelia E Tsironi[56], Kyu Hyung Park[57], Lindsay A Farrer[49-53], Anton Orlin[58], Alexander Brucker[59], Mingyao Li[60], Christine A Curcio[61], Saddek Mohand-Saïd[62-65], José-Alain Sahel[25,62-67], Isabelle Audo[62-64,68], Mustapha Benchaboune[65], Angela J Cree[69], Christina A Rennie[70], Srinivas V Goverdhan[69], Michelle Grunin[71], Shira Hagbi-Levi[71], Peter Campochiaro[11,13], Nicholas Katsanis[72-74], Frank G Holz[17], Frédéric Blond[62-64], Hélène Blanché[75], Jean-François Deleuze[75,76], Robert P Igo Jr[3], Barbara Truitt[3], Neal S Peachey[18,77], Stacy M Meuer[19], Chelsea E Myers[19], Emily L Moore[19], Ronald Klein[19], Michael A Hauser[78-80], Eric A Postel[78], Monique D Courtenay[22], Stephen G Schwartz[81], Jaclyn L Kovach[81], William K Scott[22], Gerald Liew[23], Ava G Tan[23], Bamini Gopinath[23], John C Merriam[24], R Theodore Smith[24,82], Jane C Khan[41,83,84], Humma Shahid[84,85], Anthony T Moore[25,26,86], J Allie McGrath[27], Reneé Laux[3], Milam A Brantley Jr[87], Anita

Agarwal[87], Lebriz Ersoy[28], Albert Caramoy[28], Thomas Langmann[28], Nicole T M Saksens[29], Eiko K de Jong[29], Carel B Hoyng[29], Melinda S Cain[30], Andrea J Richardson[30], Tammy M Martin[88], John Blangero[31], Daniel E Weeks[32,89], Bal Dhillon[90], Cornelia M van Duijn[35], Kimberly F Doheny[91], Jane Romm[91], Caroline C W Klaver[34,35], Caroline Hayward[33], Michael B Gorin[92,93], Michael L Klein[88], Paul N Baird[30], Anneke I den Hollander[29,94], Sascha Fauser[28], John R W Yates[25,26,84], Rando Allikmets[24,95], Jie Jin Wang[23], Debra A Schaumberg[20,96,97], Barbara E K Klein[19], Stephanie A Hagstrom[77], Itay Chowers[71], Andrew J Lotery[69], Thierry Léveillard[62-64], Kang Zhang[8,44], Murray H Brilliant[7], Alex W Hewitt[6,30,41], Anand Swaroop[38], Emily Y Chew[98], Margaret A Pericak-Vance[22], Margaret DeAngelis[48], Dwight Stambolian[10], Jonathan L Haines[3,99], Sudha K Iyengar[3], Bernhard H F Weber[4], Gonçalo R Abecasis[1] & Iris M Heid[2]

IAMDGC members' affiliations

[1]Center for Statistical Genetics, Department of Biostatistics, University of Michigan, Ann Arbor, Michigan, USA. [2]Department of Genetic Epidemiology, University of Regensburg, Regensburg, Germany. [3]Department of Epidemiology and Biostatistics, Case Western Reserve University School of Medicine, Cleveland, Ohio, USA. [4]Institute of Human Genetics, University of Regensburg, Regensburg, Germany. [5]Kidney Epidemiology and Cost Center, Department of Internal Medicine–Nephrology, University of Michigan, Ann Arbor, Michigan, USA. [6]School of Medicine, Menzies Research Institute Tasmania, University of Tasmania, Hobart, Tasmania, Australia. [7]Center for Human Genetics, Marshfield Clinic Research Foundation, Marshfield, Wisconsin, USA. [8]Department of Ophthalmology, University of California, San Diego and Veterans Affairs San Diego Health System, La Jolla, California, USA. [9]Retina Service, Massachusetts Eye and Ear, Department of Ophthalmology, Harvard Medical School, Boston, Massachusetts, USA. [10]Department of Ophthalmology, Perelman School of Medicine, University of Pennsylvania, Philadelphia, Pennsylvania, USA. [11]Department of Ophthalmology, Wilmer Eye Institute, Johns Hopkins University School of Medicine, Baltimore, Maryland, USA. [12]Department of Molecular Biology and Genetics, Johns Hopkins University School of Medicine, Baltimore, Maryland, USA. [13]Department of Neuroscience, Johns Hopkins University School of Medicine, Baltimore, Maryland, USA. [14]Institute of Genetic Medicine, Johns Hopkins University School of Medicine, Baltimore, Maryland, USA. [15]Institue de la Vision, Université Pierre et Marie Curie, Paris, France. [16]Hôpital Intercommunal de Créteil, Hôpital Henri Mondor, Université Paris Est Créteil,

Créteil, France. [17]Department of Ophthalmology, University of Bonn, Bonn, Germany. [18]Louis Stokes Cleveland Veterans Affairs Medical Center, Cleveland, Ohio, USA. [19]Department of Ophthalmology and Visual Sciences, University of Wisconsin, Madison, Wisconsin, USA. [20]Department of Epidemiology, Harvard School of Public Health, Boston, Massachusetts, USA. [21]Department of Nutrition, Harvard School of Public Health, Boston, Massachusetts, USA. [22]John P. Hussman Institute for Human Genomics, Miller School of Medicine, University of Miami, Miami, Florida, USA. [23]Centre for Vision Research, Department of Ophthalmology and Westmead Millennium Institute for Medical Research, University of Sydney, Sydney, New South Wales, Australia. [24]Department of Ophthalmology, Columbia University, New York, New York, USA. [25]University College London Institute of Ophthalmology, University College London, London, UK. [26]Moorfields Eye Hospital, London, UK. [27]Center for Human Genetics Research, Vanderbilt University Medical Center, Nashville, Tennessee, USA. [28]Department of Ophthalmology, University Hospital of Cologne, Cologne, Germany. [29]Department of Ophthalmology, Radboud University Medical Centre, Nijmegen, the Netherlands. [30]Centre for Eye Research Australia, University of Melbourne, Royal Victorian Eye and Ear Hospital, East Melbourne, Victoria, Australia. [31]South Texas Diabetes and Obesity Institute, School of Medicine, University of Texas Rio Grande Valley, Brownsville, Texas, USA. [32]Department of Biostatistics, Graduate School of Public Health, University of Pittsburgh, Pittsburgh, Pennsylvania, USA. [33]Medical Research Council (MRC) Human Genetics Unit, Institute of Genetics and Molecular Medicine, University of Edinburgh, Edinburgh, UK. [34]Department of Ophthalmology, Erasmus Medical Center, Rotterdam, the Netherlands. [35]Department of Epidemiology, Erasmus Medical Center, Rotterdam, the Netherlands. [36]Quantitative Biomedical Research Center, Department of Clinical Science, University of Texas Southwestern Medical Center, Dallas, Texas, USA. [37]Center for the Genetics of Host Defense, University of Texas Southwestern Medical Center, Dallas, Texas, USA. [38]Neurobiology, Neurodegeneration and Repair Laboratory (N-NRL), National Eye Institute, US National Institutes of Health, Bethesda, Maryland, USA. [39]Department of Ophthalmology and Visual Sciences, University of Michigan, Kellogg Eye Center, Ann Arbor, Michigan, USA. [40]Department of Ophthalmology, Flinders Medical Centre, Flinders University, Adelaide, South Australia, Australia. [41]Centre for Ophthalmology and Visual Science, Lions Eye Institute, University of Western Australia, Perth, Western Australia, Australia. [42]Sichuan Provincial Key Laboratory for Human Disease Gene Study, Hospital of the University of Electronic Science and Technology of China and Sichuan Provincial People's Hospital, Chengdu, China. [43]Sichuan Translational Medicine Hospital, Chinese Academy of

Sciences, Chengdu, China. [44]Molecular Medicine Research Center, State Key Laboratory of Biotherapy, West China Hospital, Sichuan University, Chengdu, China. [45]EyeCentre Southwest, Stuttgart, Germany. [46]University Eye Clinic, Ludwig Maximilians University, Munich, Germany. [47]Department of Ophthalmology, University Hospital Regensburg, Regensburg, Germany. [48]Department of Ophthalmology and Visual Sciences, University of Utah, Salt Lake City, Utah, USA. [49]Department of Medicine (Biomedical Genetics), Boston University Schools of Medicine and Public Health, Boston, Massachusetts, USA. [50]Department of Ophthalmology, Boston University Schools of Medicine and Public Health, Boston, Massachusetts, USA. [51]Department of Neurology, Boston University Schools of Medicine and Public Health, Boston, Massachusetts, USA. [52]Department of Epidemiology, Boston University Schools of Medicine and Public Health, Boston, Massachusetts, USA. [53]Department of Biostatistics, Boston University Schools of Medicine and Public Health, Boston, Massachusetts, USA. [54]Department of Ophthalmology, Seoul Metropolitan Government Seoul National University Boramae Medical Center, Seoul, Republic of Korea. [55]Centre for Experimental Medicine, Queen's University, Belfast, UK. [56]Department of Ophthalmology, University of Thessaly, School of Medicine, Larissa, Greece. [57]Department of Ophthalmology, Seoul National University Bundang Hospital, Seongnam, Republic of Korea. [58]Department of Ophthalmology, Weill Cornell Medical College, New York, New York, USA. [59]Scheie Eye Institute, Department of Ophthalmology, University of Pennsylvania Perelman School of Medicine, Philadelphia, Pennsylvania, USA. [60]Department of Biostatistics and Epidemiology, University of Pennsylvania Perelman School of Medicine, Philadelphia, Pennsylvania, USA. [61]Department of Ophthalmology, University of Alabama at Birmingham, Birmingham, Alabama, USA. [62]INSERM, Paris, France. [63]Institut de la Vision, Department of Genetics, Paris, France. [64]Centre National de la Recherche Scientifique (CNRS), Paris, France. [65]Centre Hospitalier National d'Ophtalmologie des Quinze-Vingts, Paris, France. [66]Fondation Ophtalmologique Adolphe de Rothschild, Paris, France. [67]Académie des Sciences–Institut de France, Paris, France. [68]Department of Molecular Genetics, Institute of Ophthalmology, London, UK. [69]Clinical and Experimental Sciences, Faculty of Medicine, University of Southampton, Southampton, UK. [70]University Hospital Southampton, Southampton, UK. [71]Department of Ophthalmology, Hadassah Hebrew University Medical Center, Jerusalem, Israel. [72]Center for Human Disease Modeling, Duke University, Durham, North Carolina, USA. [73]Department of Cell Biology, Duke University, Durham, North Carolina, USA. [74]Department of Pediatrics, Duke University, Durham, North Carolina, USA. [75]Centre d'Etude du Polymorphisme Humain (CEPH) Fondation Jean Dausset, Paris, France. [76]Commissariat à

l'Energie Atomique et aux Energies Alternatives (CEA), Institut de Génomique, Centre National de Génotypage, Evry, France. [77]Cole Eye Institute, Cleveland Clinic, Cleveland, Ohio, USA. [78]Department of Ophthalmology, Duke University Medical Center, Durham, North Carolina, USA. [79]Department of Medicine, Duke University Medical Center, Durham, North Carolina, USA. [80]Duke Molecular Physiology Institute, Duke University Medical Center, Durham, North Carolina, USA. [81]Bascom Palmer Eye Institute, University of Miami Miller School of Medicine, Naples, Florida, USA. [82]Department of Ophthalmology, New York University School of Medicine, New York, New York, USA. [83]Department of Ophthalmology, Royal Perth Hospital, Perth, Western Australia, Australia. [84]Department of Medical Genetics, Cambridge Institute for Medical Research, University of Cambridge, Cambridge, UK. [85]Department of Ophthalmology, Cambridge University Hospitals National Health Service (NHS) Foundation Trust, Cambridge, UK. [86]Department of Ophthalmology, University of California San Francisco Medical School, San Francisco, California, USA. [87]Department of Ophthalmology and Visual Sciences, Vanderbilt University, Nashville, Tennessee, USA. [88]Casey Eye Institute, Oregon Health and Science University, Portland, Oregon, USA. [89]Department of Human Genetics, Graduate School of Public Health, University of Pittsburgh, Pittsburgh, Pennsylvania, USA. [90]School of Clinical Sciences, University of Edinburgh, Edinburgh, UK. [91]Center for Inherited Disease Research (CIDR) Institute of Genetic Medicine, Johns Hopkins University School of Medicine, Baltimore, Maryland, USA. [92]Department of Ophthalmology, David Geffen School of Medicine, Stein Eye Institute, University of California, Los Angeles, Los Angeles, California, USA. [93]Department of Human Genetics, David Geffen School of Medicine, University of California, Los Angeles, Los Angeles, California, USA. [94]Department of Human Genetics, Radboud University Medical Centre, Nijmegen, the Netherlands. [95]Department of Pathology and Cell Biology, Columbia University, New York, New York, USA. [96]Center for Translational Medicine, Moran Eye Center, University of Utah School of Medicine, Salt Lake City, Utah, USA. [97]Division of Preventive Medicine, Brigham and Women's Hospital, Harvard Medical School, Boston, Massachusetts, USA. [98]Division of Epidemiology and Clinical Applications, Clinical Trials Branch, National Eye Institute, US National Institutes of Health, Bethesda, Maryland, USA. [99]Institute for Computational Biology, Case Western Reserve University School of Medicine, Cleveland, Ohio, USA.

Supplemental References

1. Consortium, G.T. (2015). Human genomics. The Genotype-Tissue Expression (GTEx) pilot analysis: multitissue gene regulation in humans. Science 348, 648-660.
2. Cipriani, V., Leung, H.T., Plagnol, V., Bunce, C., Khan, J.C., Shahid, H., Moore, A.T., Harding, S.P., Bishop, P.N., Hayward, C., et al. (2012). Genome-wide association study of age-related macular degeneration identifies associated variants in the TNXB-FKBPL-NOTCH4 region of chromosome 6p21.3. Hum Mol Genet 21, 4138-4150.
3. Yates, J.R.W., Sepp, T., Matharu, B.K., Khan, J.C., Thurlby, D.A., Shahid, H., Clayton, D.G., Hayward, C., Morgan, J., Wright, A.F., et al. (2007). Complement C3 variant and the risk of age-related macular degeneration. New Engl J Med 357, 553-561.
4. Fritsche, L.G., Igl, W., Bailey, J.N.C., Grassmann, F., Sengupta, S., Bragg-Gresham, J.L., Burdon, K.P., Hebbring, S.J., Wen, C., Gorski, M., et al. (2016). A large genome-wide association study of age-related macular degeneration highlights contributions of rare and common variants. Nature Genetics 48, 134-143.
5. Yang, J., Ferreira, T., Morris, A.P., Medland, S.E., Madden, P.A.F., Heath, A.C., Martin, N.G., Montgomery, G.W., Weedon, M.N., Loos, R.J., et al. (2012). Conditional and joint multiple-SNP analysis of GWAS summary statistics identifies additional variants influencing complex traits. Nature Genetics 44, 369-U170.
